# Melting properties of amino acids and their solubility in water†

**DOI:** 10.1039/d0ra08947h

**Published:** 2020-12-15

**Authors:** Hoang Tam Do, Yeong Zen Chua, Aarti Kumar, Daniel Pabsch, Moritz Hallermann, Dzmitry Zaitsau, Christoph Schick, Christoph Held

**Affiliations:** Laboratory of Thermodynamics, TU Dortmund University Emil-Figge-Str. 70 44227 Dortmund Germany christoph.held@tu-dortmund.de; Institute of Physics, University of Rostock Albert-Einstein-Str. 23-24 18051 Rostock Germany yeong.chua@uni-rostock.de; Competence Centre CALOR, University of Rostock Albert-Einstein-Str. 25 18051 Rostock Germany; Institute of Chemistry, University of Rostock Dr-Lorenz-Weg 2 18051 Rostock Germany; Chemical Institute A. M. Butlerov, Kazan Federal University 18 Kremlyovskaya Street Kazan 420008 Russian Federation

## Abstract

The state-of-the-art unit operation for separation and purification of amino acids is still crystallization, which requires solubility data and melting properties of pure compounds. Since measuring solubility is time-consuming, prediction tools are desired. Further, melting properties are not yet available due to decomposition of amino acids upon slow heating. In this work, melting properties of twenty amino acids (except Met) were measured by Fast Scanning Calorimetry (FSC) with heating rates up to 20 000 K s^−1^. PC-SAFT was used to predict interactions in amino acid + water systems. Additionally, solubility, pH, and PXRD was measured. By combining FSC and PC-SAFT, the solubility of 15 amino acids was successfully predicted in a wide temperature range in good agreement with the experimental data. Thus, this work provides melting properties of amino acids for the first time and highlights the usefulness of such data to predict material properties such as aqueous solubility of amino acids.

## Abstract 2

We report the melting properties of amino acids for the first time and highlight the usefulness of such data to predict material properties such as aqueous solubility of amino acids.

## Graphical abstract

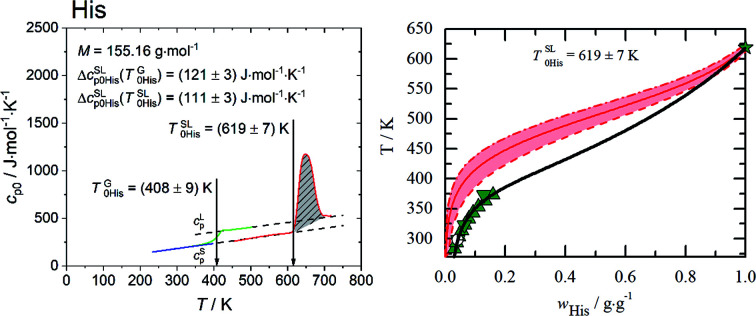

## Introduction

Commonly proteins are represented by the set of twenty “standard” α-amino acids (AA). These either exist as a monomer or they are bound as building blocks in peptides and proteins.^1^ Since their isolation in the 19^th^ century the physical and chemical properties of AA have been widely investigated because of their crucial importance in nature and by relevance for industrial processes.^2,3^ The applied separation unit of fractional crystallization is still state-of-the-art.^4,5^ This requires basic understanding of the melting temperatures as well as the solubility behavior to further develop and optimize the downstream process.^6^

However, consistent melting temperatures are still not available for the amino acids. Further, aqueous AA solubility studies have not been checked for consistency. Such studies were carried out in the early 20^th^ century focusing on AA + water.^7–12^ Many of these works were performed in a narrow temperature range, without pH measurements and analysis of the crystal structure of AA between its pure component and the solid in saturated solutions.

Undoubtedly measuring solubility data is expensive. Hence, prediction of AA solubility in a wide temperature range based on physical properties such as melting properties is highly desired. Unfortunately, conventional methods, *e.g.* Differential Scanning Calorimetry (DSC), are not applicable to determine the melting properties of AA due to thermal decomposition upon slow heating rates.^13^ Experimental melting properties is available in literature only for two AA: glycine, l-alanine^14^ and l-arginine.^15^

In the current study we continue this work and present the melting properties of twenty proteinogenic AA characterized with Fast Scanning Calorimetry (FSC). FSC with scanning rates up to 20 000 K s^−1^ has been established as a reliable device to study the melting thermodynamics of thermally labile biomolecules, *e.g.* bio-polymers,^16,17^ low molecular mass pharmaceuticals^18^ and nucleobases.^19,20^ The experimental melting properties are applied as an input for the thermodynamic framework PC-SAFT to predict the aqueous AA solubility. Additionally, a solid–liquid equilibrium between solid AA and the saturated liquid aqueous phase was applied. Assuming pure solid amino-acid phase the solubility *x*^L^_i_ is determined according to Prausnitz^21^ as:1
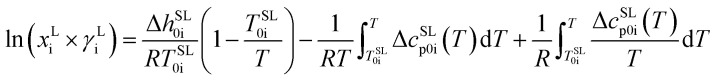
2
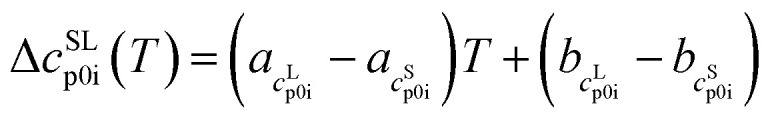
with *γ*^L^_i_ as the activity coefficient of AA, *R* the universal gas constant, Δ*h*^SL^_0i_ the melting enthalpy at melting temperature, *T*^SL^_0i_ the melting temperature, and Δ*c*^SL^_p0i_(*T*) the temperature-dependent difference between the heat capacities in liquid (L) and solid (S) state of a pure AA. In eqn (2), Δ*c*^SL^_p0i_(*T*) was assumed to show a linear temperature dependence with 
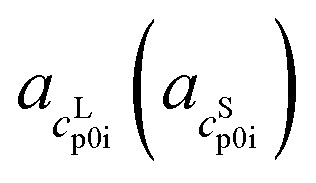
 and 
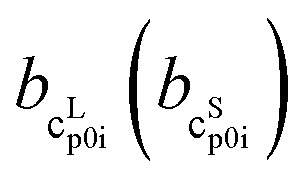
 as the slope and the intercept of the heat capacities, respectively. The solubility increase (decrease) with decrease (increase) of Δ*h*^SL^_0i_, while increase in *T*^SL^_0i_ and Δ*c*^SL^_p0i_(*T*) reduces the slope of the solubility–temperature curve to less temperature-dependency. The influence of the solvent is accounted by *γ*^L^_i_, which describes interactions between studied compound and solvent in the liquid phase. The crystal structure of the AA was measured by Powder X-ray Diffraction (PXRD). Eqn (1) is only valid for the neutral form of the molecule, which was confirmed by pH measurements of the saturated solutions.

Detail workflow of this work is illustrated in ESI Fig. S1.† The abbreviation of AA are in ESI Table S1.†

## Methodology

### Materials and reagents

Twenty proteinogenic AA investigated in this work are listed in ESI Table S1.† All AA were of commercial origin and used without additional purification. The Millipore-Q grade water for the solubility measurements was directly taken in the lab.

### Melting measurements with FSC

Experimental FSC melting properties measurements were carried out by using Flash DSC 1 (Mettler Toledo) with the calorimetric twin chip sensor UFS1.^22,23^ The experimental FSC study of the AA was given in the previous works, where detailed experimental description of FSC method has been presented.^14,24^

All measurements were conducted under inert atmospheres of dry nitrogen (dew point lower than 150 K) with a flow rate of 50 mL min^−1^. The sensors were conditioned according to manufacturer's procedure and the temperature was calibrated with recommended calibration metals (indium, bismuth and tin).

The experimental FSC procedure consists of three measurement stages, as presented in the temperature–time profile in ESI Fig. S2.† The starting temperature is set to 303 K to reduce the measuring time, as starting temperature below 303 K requires a cooler and long system equilibration times.

For the first stage (#1 to #4), the temperature range from 303 K to 473 K and constant scanning rate 2000 K s^−1^ were selected to assure the high reproducibility of the heating and cooling cycles. The reproducibility is indirect proof indicating that sample mass loss due to sublimation and decomposition has not occurred, and that volatile impurities or water were absent. It is also indicating that the sample was measured in its anhydrous form. The sample mass (without silicon oil) is determined in this stage as *m*_0_ = *M*_i_ [g mol^−1^] × *C*^S^_p0i_ [J K^−1^]/*c*^S^_p0i_ [J mol^−1^ K^−1^], where *C*^S^_p0i_ [J K^−1^] is the total heat capacity of the sample from the first FSC stage and *c*^S^_p0i_ [J mol^−1^ K^−1^] is specific heat capacity obtained DSC measurements (Pyris 1, PerkinElmer, USA).^14,16,19,24–26^

In the second stage, the melting properties were determined in heating step #5. To improve the thermal contact between the sample and the sensor, silicon oil can be added to the sample before heating step #5. All the samples used in FSC measurements were relatively small (less than 100 ng) and for such small samples, the surface-to-volume ratio is rather high, what leads to increase in mass loss due to sublimation or evaporation at higher temperature. This effect is especially prominent for small molecules like AA. Therefore silicon oil not only improves the thermal contact but additionally coats the sample surface and suppresses the mass loss of the sample due to sublimation or evaporation. The heating rates of step #5 typically ranged from 2000 K s^−1^ to 10 000 K s^−1^. However for a few extremely thermally labile AA, *e.g.* Ile, Asn, Cys, higher heating rates up to 20 000 K s^−1^ were applied together with silicon oil coating to further minimize the sublimation or evaporation processes. Unfortunately even with these methods, sublimation or evaporation of Met cannot be suppressed enough. The melting and evaporation process were overlapping each other which leads to an unsuccessful determination of melting properties.

**Fig. 1:**
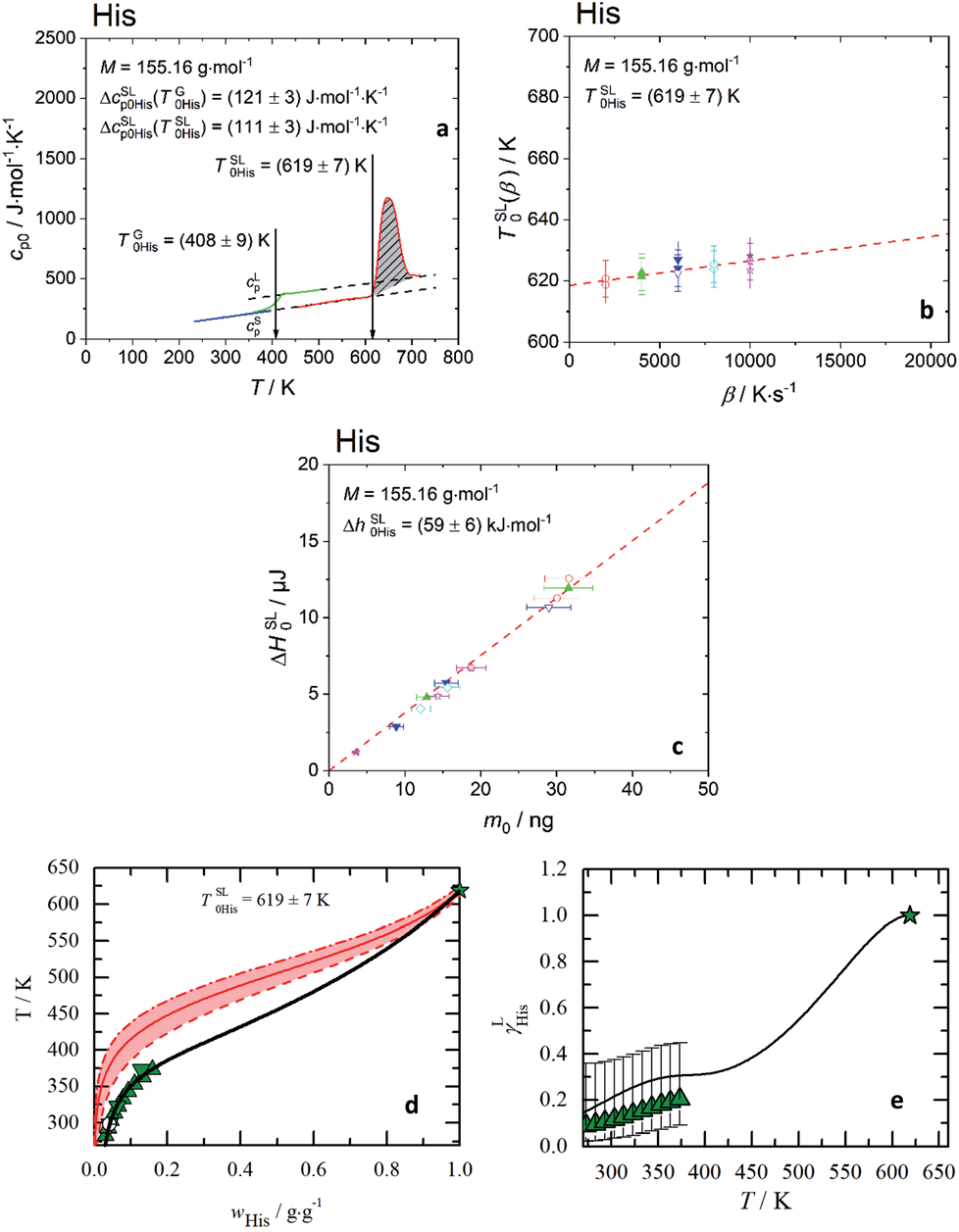
Melting properties of His. (a) Specific heat capacity of His determined experimentally with FSC (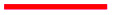) and for glass transition step of ultra-fast quenched melted His (without silicon oil) (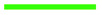) and DSC for heat capacity of solid, *c*^S^_p0i_ (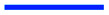). The area under the melting peak (
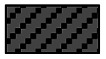
) indicates Δ*h*^SL^_0i_, while onset temperature corresponds to *T*^SL^_0i_. Δ*c*^SL^_p0i_ is determined at glass transition temperature, Δ*c*^SL^_p0i_(*T*^G^_0i_) and adjusted to melting temperature, Δ*c*^SL^_p0i_(*T*^SL^_0i_). (b) Melting temperature *vs.* heating rate diagram. Red line is the linear extrapolation to zero heating rate. The uncertainty is the standard deviation of multiple measurements. (c) Enthalpy, Δ*H*^SL^_0i_, of His with respect to sample mass, *m*_0_, regardless of the scanning rates *β* [K s^−1^]. The slope of the linear fit through zero origin (line) signifies Δ*h*^SL^_0i_. The applied scanning rates were 2000 K s^−1^ (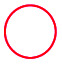), 4000 K s^−1^ (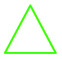 up-triangles), 6000 K s^−1^ (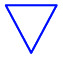), 8000 K s^−1^ (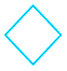) and 10 000 K s^−1^ (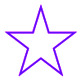). Solid symbols (without silicon oil), empty symbols (with silicon oil). The melting properties of all twenty proteinogenic AA are shown in ESI Fig. S3 and S4.† The *T*^SL^_0i_, Δ*h*^SL^_0i_, Δ*c*^SL^_p0i_(*T*^G^_0i_) and Δ*c*^SL^_p0i_(*T*^SL^_0i_) for each AA are listed in Table 1. (d) His aqueous solubility as temperature *vs.* weight fraction diagram. The red area presents the solubility modeling assuming *γ*^L^_i_ = 1 (eqn (1)) in the range of the uncertainties of the melting properties. 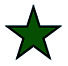: *T*^SL^_0His_ = (619 ± 7) K. Symbols represent literature data (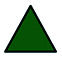: Kustov,^32^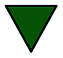: Amend^9^). (e) Activity coefficients *vs.* temperature diagram. (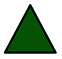: Kustov^32^) uncertainties are based on the uncertainties of the melting enthalpy. <svg xmlns="http://www.w3.org/2000/svg" version="1.0" width="32.333333pt" height="16.000000pt" viewBox="0 0 32.333333 16.000000" preserveAspectRatio="xMidYMid meet"><metadata> Created by potrace 1.16, written by Peter Selinger 2001-2019 </metadata><g transform="translate(1.000000,15.000000) scale(0.014583,-0.014583)" fill="currentColor" stroke="none"><path d="M80 480 l0 -160 960 0 960 0 0 160 0 160 -960 0 -960 0 0 -160z"/></g></svg> : PC-SAFT.

In the heating step #5 the shaded grey area in Fig. 1(a) in the temperature range of the melting peak was designated as the melting enthalpy, Δ*H*^SL^_0i_ [J], while the onset of the melting peak is a scanning rate dependent melting temperature, *T*^SL^_0i_(*β*). The specific melting enthalpy, Δ*h*^SL^_0i_, is defined as a ratio Δ*h*^SL^_0i_ = Δ*H*^SL^_0i_ × *M*/*m*_0_, where *M* is the molar mass of AA and *m*_0_ is the mass of the sample.

After heating step #5, the molten samples without silicon oil were quenched rapidly to retain the sample in the liquid state below the melting temperature without crystallization. During the heating and cooling cycles (#8 to #11) in third stage a step change in specific heat capacity corresponding to glass transition from amorphous solid of AA to liquid (supercooled) state was observed. Due to complications in avoiding sublimation or evaporation mass loss of the samples at high temperatures in the current state of FSC technique, the glass transition can be determined only for half of the 20 proteinogenic AA.

### Measurement of solubility

AA are widely investigated and their aqueous solubility data are readily available in literature. Most of the studies are carried out by using the gravimetric method. However, in some cases a discrepancy between literature data and experimental values is observed. In this work an excess amount of solute is added to water till the saturated solution in equilibrium with the solid solute is formed. The compounds were shaken and equilibrated isothermally (at least 72 h) to ensure the solid–liquid equilibrium is reached. After this a defined amount of the saturated liquid phase is withdrawn and weighed. The sample solution is placed in a drying chamber and a vacuum chamber to ensure total evaporation of the water. The remaining solid was weighed again and thus the solubility determined. Additionally often pH values of the saturated solutions and crystal-structure studies of solid phase are missing, which are important since the crystal structure of the pure compound and the solid compound in equilibrium state is not allowed to change during the solubility determination and solubility model. In order to complete the missing information about crystal structure and pH values, AA solubility (for all 20 AA) was determined gravimetrically at *T* = 298.15 K in three independent unbuffered aqueous solutions. The solutions were mixed for 24 h and left without further shaking for equilibration for 48 h. Then 200 μL of the saturated liquid phase was withdrawn for the solubility determination. The pH measurement of the unbuffered saturated solutions in solid–liquid equilibrium, as well as, the crystal structure of the initial pure AA (from the supplier), and of the solid phase equilibrated with saturated liquid phase were determined using pH meter with a standard uncertainty of ±0.01 and Powder X-ray Diffractometer (PXRD, Miniflex 600, Rigaku, Japan, operating temperature (295.15 K) and pressure (1 atm), speed scan 5° min^−1^ from 2° to 35° in 0.02° steps, voltage 40 kV, current 15 mA, type of radiation Cu Kα anode), respectively. All the pH values of the saturated solutions are listed Table 3 and the PXRD diffractograms are shown in the ESI Fig. S25–S34.†

### PC-SAFT

The successful prediction of AA solubility using eqn (1) requires the corresponding activity coefficient and experimental melting properties. The activity coefficient is the ratio of the fugacity coefficient *φ*^L^_i_ at the solubility mole fraction to the fugacity coefficient *φ*^L^_0i_ of the pure-component. In this work the PC-SAFT (Perturbed-Chain Statistical Associating Fluid Theory) equation of state is used and the fugacity coefficient is expressed as follows3
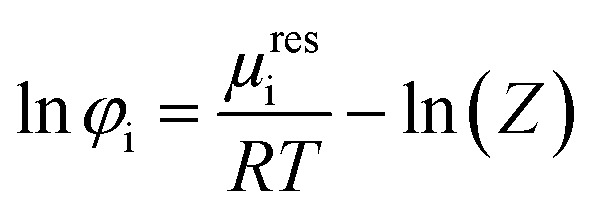
where *μ*^res^_i_ represents the residual chemical potential and *Z* the compressibility. The calculation of *μ*^res^_i_ and *Z* requires the residual Helmholtz energy *a*^res^ which is expressed in this work as

4 *a*^res^ = *a*^hc^ + *a*^disp^ + *a*^assoc^

where *a*^hc^, *a*^disp^ and *a*^assoc^ are the Helmholtz energy contributions “hard chain”, “dispersion” and “association”, respectively. In this work the original PC-SAFT from Gross and Sadowski^26^ is used, where all required contributions have already been implemented. For mixtures (here water + AA), the conventional Berthelot–Lorentz combining rules were applied to describe the interactions between two components i and j5
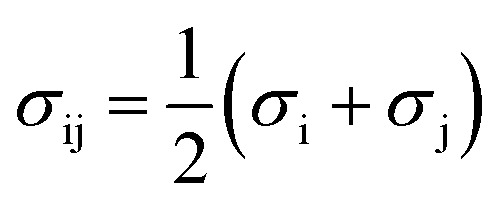
6
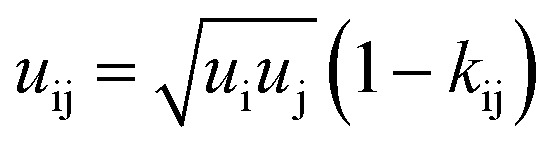
where *k*_ij_ is the binary interaction parameter to describe deviations from the geometric mean of the dispersion-energy parameters of two components i and j (*i.e.*, water and AA). The interaction parameter *k*_ij_ was fitted to osmotic-coefficient data at *T* = 298.15 K. For some AA, a linearly temperature-dependent binary interaction parameter *k*_ij_(*T*) was available in the literature, expressed as:

7 *k*_ij_(*T*) = *k*_ij_298.15 K__ + *k*_ij_*T*__ (*T* − 298.15 K)

In this work *k*_ij_(*T*) was fitted to solubility data at higher temperatures.

In the current work the AA were considered as associating fluids, and each one association site was assigned for the amine group and for the carboxylic group, respectively. In case of specific side chains of the AA, additional association site were added depending on a proton donator (*e.g.* Glu 1 : 2) or proton acceptor (Gln 2 : 1). The PC-SAFT pure-component parameters for most of the AA are already published^11^ and will be utilized in this work, except for Glu and Asp with improved parameters, and for Trp with completely new parameters (listed in Table 2). The pure-component parameters were fitted to osmotic-coefficient data and density data of aqueous solutions at *T* = 298.15 K. For some further AA new experimental data for osmotic coefficients and mixture density was added in this work. The diagrams of the fitted osmotic coefficients and mixture densities are shown in Fig. S5–S24 in the ESI.† Water was modeled with the 2B association scheme with a temperature-dependent segment diameter as it was used already in our previous work.^14^ The PC-SAFT pure-component parameters as well as binary interaction parameters between the AA and water according eqn (6) used in this work are listed in Table 2.

## Results

### Experimental melting properties

The melting properties of 19 proteinogenic AA (except Met) were characterized experimentally with FSC. The FSC experimental results for His as a representation are presented in Fig. 1(a–c), while for all other AA in ESI Fig. S3 and S4.†

Ideally, a direct determination of Δ*c*^SL^_p0i_ at *T*^SL^_0i_ is preferable from the melting curve. However, this is not possible for some AA due to the mass loss caused by sublimation or evaporation after melting. The mass loss of the sample is indicated by a baseline drop below *c*^S^_p0i_ after the melting, even though the sample was cooled down rapidly right after the melting to minimize the mass loss at high temperature. If complete mass loss and crystallization are avoided, a glass transition step at *T*^G^_0i_ from glassy to supercooled liquid AA is shown as solid green line.

For low volatile samples such His or Arg (ESI Fig. S3†), the liquid phase immediately after the melting (solid red line) is in accordance with the *c*^L^_p0i_ above glass transition. This indicates that the linear extrapolation from *c*^L^_p0i_ of the glass transition to *T*^SL^_0i_ is applicable. For consistency reasons this extrapolation was applied for all AA with measured glass transition. For high volatile AA (Gly, Ala, Val, Leu, Ile, Pro, Lys, Phe, Cys) without measurable glass transition, Δ*c*^SL^_p0i_(*T*) was estimated as explained in the discussion.

The *c*^L^_p0i_ of the glass transition was fitted linearly with 
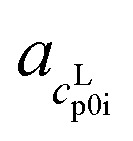
 as slope and 
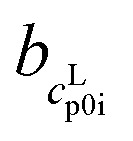
 as intercept, while the *c*^S^_p0i_ determined from DSC as solid blue line is fitted linearly with 
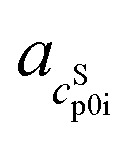
and 
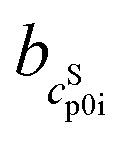
. The heat capacity of crystal and glass are assumed to be equal, especially at temperatures close to *T*^G^. This assumption is commonly accepted, *e.g.* indomethacin,^27^ saccharides,^28^*o*-terphenyl,^29^ selenium,^30^ poly-*p*-dioxanone.^31^ The heat capacity difference between crystal and glass of such components does not differ by more than 5 to 10%. This difference is also within the uncertainty of our investigation. Nevertheless, we have to acknowledge that there is a difference in the heat capacity, which may influence the result of our investigation. Nevertheless, in sum the difference between heat capacity of solid and glass phases are worst-case assumed to be <10%. Thus, heat capacity of solid was set equal to the glass. This allows indirect determination of Δ*c*^SL^_p0i_(*T*^G^_0i_) at glass transition temperature and adjustment to melting temperature, Δ*c*^SL^_p0i_(*T*^SL^_0i_), which is required in eqn (1).^24^

In Fig. 1(b), the melting temperature was determined by extrapolating the onset melting temperature at different scanning rates to zero scanning rate *β*, Δ*T*^SL^_0i_(*β* → 0), considering the thermal lag and possible superheating.^33–35^ The slope of the linear fit through zero origin in Fig. 1(c) signifies the specific melting enthalpy, Δ*h*^SL^_0i_, where the Δ*H*^SL^_0i_ depends linearly on the sample mass, regardless of the scanning rates. Samples were measured with and without encapsulating in silicon oil. The good agreement of melting temperatures and melting enthalpies between both samples indicates the absence of chemical interaction between AA and silicon oil.

The experimental melting properties measured by FSC are listed in Table 1.

**Table 1:** Molar mass (*M*), experimental glass and melting properties determined with FSC in this work (*T*^SL^_0i_, *T*^G^_0i_, Δ*h*^SL^_0i_, Δ*s*^SL^_0i_, Δ*c*^SL^_p0i_(*T*^G^_0i_) and Δ*c*^SL^_p0i_(*T*^SL^_0i_)) of the pure 19 proteinogenic AA. The uncertainties represents the standard deviations of multiple measurements

	*M*/g mol^−1^	*T* ^G^_0i_/K	*T* ^SL^_0i_/K	Δ*h*^SL^_0i_/kJ mol^−1^	Δ*s*^SL^_0i_/kJ mol^−1^	Δ*c*^SL^_p0i_(*T*^G^_0i_)/J mol^−1^ K^−1^	Δ*c*^SL^_p0i_(*T*^SL^_0i_)/J mol^−1^ K^−1^
**AA with non-polar substituents**							
Gly^a^	75.07	—	569 ± 9	22 ± 3	0.038 ± 0.005	—	—
Ala^a^	89.10	—	608 ± 9	23 ± 3	0.038 ± 0.005	—	—
Val	117.15	—	529 ± 7	44 ± 6	0.083 ± 0.011	—	—
Leu	131.18	—	518 ± 8	43 ± 5	0.082 ± 0.011	—	—
Ile	131.18	—	595 ± 7	43 ± 6	0.083 ± 0.011	—	—
Pro	115.14	—	527 ± 7	19 ± 3	0.036 ± 0.005	—	—

**AA with polar substituents**							
Ser	105.10	337 ± 2	519 ± 7	28 ± 3	0.053 ± 0.006	64 ± 3	50 ± 3
Thr	119.12	355 ± 4	587 ± 9	34 ± 5	0.058 ± 0.035	69 ± 1	63 ± 9

**AA with acidic substituents**							
Asp	133.11	386 ± 16	610 ± 7	35 ± 5	0.057 ± 0.006	93 ± 4	42 ± 4
Asn	132.12	466 ± 11	582 ± 7	33 ± 4	0.055 ± 0.007	80 ± 2	52 ± 2
Glu	147.13	330 ± 5	566 ± 7	46 ± 5	0.078 ± 0.006	63 ± 5	25 ± 5
Gln	146.15	323 ± 5	589 ± 7	50 ± 6	0.076 ± 0.010	79 ± 2	80 ± 2

**AA with basic substituents**							
Arg	174.21	362 ± 3	558 ± 7	28 ± 4	0.051 ± 0.007	107 ± 5	35 ± 5
His	155.16	408 ± 9	619 ± 7	59 ± 6	0.095 ± 0.011	120 ± 3	113 ± 3
Lys	146.19	—	529 ± 9	22 ± 3	0.042 ± 0.004	—	—

**AA with aromatic substituents**							
Phe	165.20	—	579 ± 7	58 ± 7	0.099 ± 0.013	—	—
Tyr	181.20	405 ± 3	678 ± 7	47 ± 6	0.069 ± 0.009	65 ± 1	63 ± 1
Trp	204.23	433 ± 3	620 ± 7	60 ± 7	0.097 ± 0.012	99 ± 4	22 ± 4

**AA with sulfuric substituents**							
Cys	121.16	—	604 ± 7	45 ± 8	0.074 ± 0.014	—	—

a Already published in previous work.^14^

## Discussion

First, without any thermodynamic model – the activity coefficient *γ*^L^_i_ is assumed to be one (ideal mixture); this results in direct relation between the melting properties and the solubility according to eqn (1), where low melting temperature and enthalpy correspond to high solubility. The rule is true for the series of basic AA (Arg, His, Lys) as well as for aromatic AA (Phe, Tyr, Trp). However, the rule is not valid for the acidic AA (Asp, Glu) and their amides (Asn, Gln). The amide AA are better soluble in water compared to their acidic pendants despite the fact that the latter have lower melting temperatures and enthalpies. This wrong description of solubility using ideal mixture can only lead to the conclusion that *γ*^L^_i_ has to be taken into account to describe the interactions in the liquid phase. This can be quantified by another example. The solubility of His in an ideal mixture was calculated using melting properties in the range of their uncertainties. The result is shown as red area in Fig. 1(d). The experimental data of His solubility in water are not within the red area, giving the conclusion that *γ*^L^_i_ should be lower than one to match solubility according to eqn (1).

In Fig. 1(e) the PC-SAFT predicted *γ*^L^_His_ of His of the saturated solutions are presented. This is compared with values determined by using experimental FSC melting properties and experimental solubility data solved by eqn (1) to yield *γ*^L^_i_. It can be observed that *γ*^L^_His_ values are far away from being one, and that the results of PC-SAFT prediction agrees with the experimental values within FSC uncertainty. The activity coefficients change with the temperature till it approaches unity at the melting temperature. The PC-SAFT predicted values of *γ*^sat^_298.15 K_ for each AA at *T* = 298.15 K are listed in Table 2.

**Table 2:** PC-SAFT pure-component parameters and binary interaction parameters used to evaluate *k*_ij_ according to eqn (7). Solubility ratio of one AA at two temperatures *w*_323.15 K_/*w*_298.15 K_, ARD between PC-SAFT and experimental solubility for *N*_dp_ data points, activity coefficients in saturated solutions at *T* = 298.15 K, and PXRD transitions

	*m* ^seg^_i_	*σ*_i_/Å	*u*_i_/*k*_B_/K	*ε* ^AiBi^/*k*_B_/K	*κ* ^AiBi^	*N*	*k*_ij,298.15 K_/10^−2^	*k*_ij,*T* K_/10^−4^	*w*_323.15 K_/*w*_298.15 K_	ARD/%	*N*_dp_/ref.	*γ* ^sat^_298.15 K_	PXRD trans.
H_2_O	1.2047	a	353.94	2425.67	0.045	—	—	—	—	—	—	—	—

**AA with non-polar substituents**													
Gly^c^	4.850	2.327	216.960	2598.060	0.039	2	−5.85^c^	—	1.392	3.84	10/36	0.305	—
Ala^c^	5.465	2.522	287.590	3176.600	0.082	2	−6.12^c^	2.91^c^	1.292	1.66	10/11	0.235	—
Val^b^	7.485	2.589	306.410	3183.800	0.039	2	−7.57^b^	3.85^b^	1.223	2.07	7/37	0.059	—
Leu^b^	8.304	2.700	330.000	3600.000	0.020	2	−6.39^b^	5.00^d^	1.245	3.63	19/11	0.129	—
Ile^b^	8.241	2.586	281.884	2207.529	0.001	2	−8.75^b^	2.70^b^	1.199	4.60	8/38	0.043	—
Pro^b^	6.981	2.548	289.720	5527.750	0.036	2	−6.99^b^	—	1.192	—	—	—	*x*

**AA with polar substituents**													
Ser^b^	7.024	2.284	236.920	2671.930	0.039	3	−2.57^b^	4.00^d^	1.526	0.76	5/12	0.193	*x*
Thr^b^	6.329	2.606	325.370	2519.410	0.039	3	−2.78^b^	1.25^d^	1.388	0.44	8/39	0.465	—

**AA with acidic substituents**													
Asp^d^	5.827	2.522	287.625	2544.234	0.041	3	1.45^d^	—	1.889	8.17	16/40	5.825	—
Asn^b^	3.000	3.367	280.000	3265.670	0.044	3	0.00^b^	—	2.879	—	—	—	*x*
Glu^d^	6.831	2.560	227.192	2544.234	0.041	3	−4.45^d^	—	2.501	4.52	23/41	0.324	—
Gln^d^	9.289	2.360	273.555	2637.341	0.020	3	−5.18^d^	—	1.992	3.33	8/42	0.114	—

**AA with basic substituents**													
Arg^b^	9.908	2.657	349.710	2555.450	0.039	4	−1.45^b^	—	1.848	10.1	11/37	0.969	—
His^d^	9.088	2.473	281.954	2640.981	0.078	3	−3.89^d^	0.91^d^	1.517	6.62	11/32	0.205	—
Lys^b^	11.673	2.378	301.210	3787.310	0.033	3	−7.07^b^	—	1.358	—	—		*x*

**AA with aromatic substituents**													
Phe^d^	9.310	2.690	391.827	3206.094	0.010	2	−5.18^d^	—	1.502	14.2	17/7	1.755	—
Tyr^b^	8.139	2.280	289.370	2500.000	0.040	3	0.0227	—	1.934	18.8	11/36	11.17	—
Trp^d^	10.577	2.825	260.641	2563.249	0.024	3	−7.68	1.78^d^	1.493	1.66	11/36	0.021	—

**AA with sulfuric substituents**													
Cys^b^	7.739	2.384	322.910	1964.000	0.010	3	−2.35	—	1.755	—	—	—	*x*
Met^b^	16.026	2.150	220.370	1964.000	0.010	3	−1.43	1.57	1.416	—	—	—	—

a Temperature-dependent segment diameter *σ* = 2.7927 + 10.11 exp(−0.01775*T*) − 1.417 exp(−0.01146*T*).

b Pure-component parameters from Held *et al.*^10^

c Pure-component parameters from Chua *et al.*^14^

d Pure-component parameters from this work.

### PXRD measurements

The hydration of AA has been widely reported in literature.^44–49^ In this work the PXRD measurement lead to further investigations in terms of possible polymorphs or formation of hydrates. Unfortunately, some AA were found to form hydrates (Ser,^12^ Lys,^50^ Asn, Pro), which does not allow the application of eqn (1) since the solid crystal in solution as well as for the melting properties must be the same. All the PXRD measurements were performed for the saturated solutions at *T* = 298.15 K and are shown in ESI Fig. S25–S34.†

### pH measurements

The pH measurement in aqueous solution of AA was conducted in order to ensure that only one neutral species (>99%) was present in the saturated solution. Asp (≈95%), Arg (≈90%), Glu (≈88%) have less neutral species present in the saturated solutions, but this is still sufficient for PC-SAFT modeling, unfortunately not for Lys (≈70%), for which Lys was excluded from the PC-SAFT modeling. The pH values for all AA solutions are listed in Table 3.

**Table 3:** Solubility *w*^sat^_298.15 K_, pH values of saturated solutions under study (uncertainties represents the standard deviations of multiple measurements, isoelectric point (pI) from literature and melting properties used in PC-SAFT: melting temperature *T*^SL^_0i_, melting enthalpy Δ*h*^SL^_0i_ and the slope (
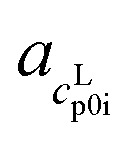
, 
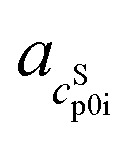
) and interception (
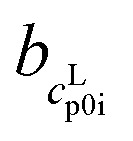
, 
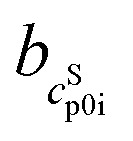
) of the heat capacity of liquid and solid, and difference in the heat capacity at melting temperature Δ*c*^SL^_p0i_(*T*^SL^_0i_)

	*w* ^sat^_298.15 K_/g g^−1^	pH^sat^_298.15 K_	pI^36^	*T* ^SL^_0i_/K	Δ*h*^SL^_0i_/kJ mol^−1^	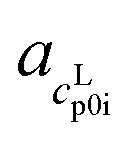 /J mol^−1^ K^−2^	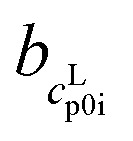 /J mol^−1^ K^−1^	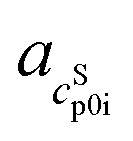 /J mol^−1^ K^−2^	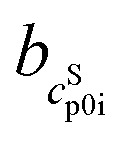 /J mol^−1^ K^−1^	Δ*c*^SL^_p0i_(*T*^SL^_0i_)/J mol^−1^ K^−1^
**AA with non-polar substituents**										
Gly	0.2019 ± 0.0020^b^	6.32 ± 0.04	5.97	569^a^	24.96^a^	0.225	62.681	0.266	21.033	18.59
Ala	0.1415 ± 0.0015^b^	6.33 ± 0.02	6.00	608^a^	25.99^a^	0.267	64.148	0.324	24.225	5.26
Val	0.0553 ± 0.0006^b^	6.08 ± 0.07	5.96	529	46.72	0.351	106.488	0.453	32.573	20.00
Leu	0.0237 ± 0.0003^b^	5.68 ± 0.15	5.98	518	49.09	0.525	71.622	0.577	24.322	10.15
Ile	0.0329 ± 0.0003^b^	6.22 ± 0.14	6.02	595	47.11	0.459	87.228	0.512	35.624	20.39
Pro	0.6365 ± 0.0154^b^	7.26 ± 0.07	6.30	—	—	—	—	—	—	—

**AA with polar substituents**										
Ser^c^	0.2867 ± 0.0123^bb^	6.01 ± 0.02	5.68	519	32.98	0.267	121.318	0.346	31.028	49.38
Thr	0.0894 ± 0.0008^b^	5.87 ± 0.01	5.60	587	36.64	0.379	125.276	0.406	47.019	62.18

**AA with acidic substituents**										
Asp	0.0057 ± 0.0002^b^	3.05 ± 0.01	2.77	610	35.73	0.176	213.341	0.397	37.182	41.37
Asn	0.0267 ± 0.0016^b^	5.13 ± 0.05	5.41	—	—	—	—	—	—	—
Glu	0.0088 ± 0.0003^b^	3.28 ± 0.04	3.22	566	48.24	0.321	147.115	0.481	32.014	24.33
Gln	0.0405 ± 0.0002^b^	5.01 ± 0.04	5.65	589	51.96	0.474	129.528	0.500	34.849	79.19

**AA with basic substituents**										
Arg	0.1639 ± 0.0034^b^	11.45 ± 0.02	10.8	558	28.64	0.326	265.689	0.690	27.698	34.83
His	0.0414 ± 0.0003^b^	7.75 ± 0.05	7.59	619	56.01	0.507	152.902	0.537	21.854	112.80
Lys	0.5197 ± 0.1256^b^	10.66 ± 0.10	9.74	—	—	—	—	—	—	—

**AA with aromatic substituents**										
Phe	0.0291 ± 0.0007^b^	5.99 ± 0.20	5.48	579	60.66	0.496	280.823	0.635	15.731	184.37
Tyr	0.0006 ± 0.0001^b^	5.77 ± 0.34	5.66	678	49.77	0.664	93.511	0.681	19.229	62.74
Trp	0.0138 ± 0.0001^b^	5.08 ± 0.11	5.89	620	65.55	0.351	289.570	0.758	15.771	21.82

**AA with sulfuric substituents**										
Cys	0.1419 ± 0.0060^b^	5.14 ± 0.03	5.74	—	—	—	—	—	—	—
Met	0.0536 ± 0.0014^b^	5.91 ± 0.03	5.07	—	—	—	—	—	—	—

a Published in ref. 14.

b Measured in this work.

c Melting properties of anhydrous Ser.

### Solubility predictions with PC-SAFT

The solubility of all AA was predicted with PC-SAFT based on the experimental melting properties measured with FSC. Prediction means that all PC-SAFT pure-component parameters were fit to non-solubility properties such as osmotic coefficients and mixture densities at *T* = 298.15 K in water. The deviations between PC-SAFT values and the experimental solubility were quantified with the absolute relative deviations (ARD) according to eqn (8)8
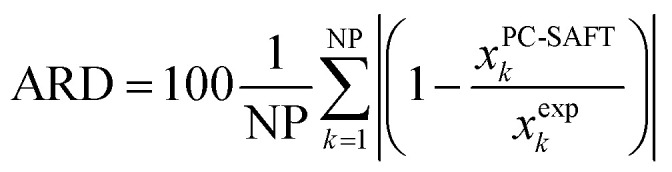
where NP is the number of the available experimental solubility points, *x*^PC-SAFT^_*k*_ and *x*^exp^_*k*_ are the PC-SAFT predicted and the experimental solubility, respectively.

As shown recently^12^ the Δ*h*^SL^_0i_ has the highest influence on the solubility prediction. Unfortunately, Δ*h*^SL^_0i_ values from FSC have rather high uncertainty up to 20%, in comparison to the Δ*c*^SL^_p0i_(*T*^SL^_0i_) (up to 5%) and *T*^SL^_0i_ (up to 2%). Therefore, FSC experimental results of Δ*c*^SL^_p0i_(*T*^SL^_0i_) and *T*^SL^_0i_ were utilized as input for solubility predictions with PC-SAFT directly, *i.e.* without varying within the experimental uncertainty. In contrast, the Δ*h*^SL^_0i_ was adjusted (within the range of uncertainty of the FSC results) to experimental solubility data at 298.15 K. As a result, the FSC data for Δ*h*^SL^_0i_ in (Table 1) and the PC-SAFT fit for Δ*h*^SL^_0i_ (Table 3) are nearly identical, which proves the general suitability of PC-SAFT method for the mixtures considered in the present work, where the predicted PC-SAFT solubility is in good agreement with experimental solubility Table 2.

Most of the PC-SAFT parameters were already available in the literature.^8^ These are listed in Table 2 together with binary interaction parameters between water and AA. The parameter *k*_ij_(*T*) was applied for AA with a rather low temperature dependency of solubility. Therefore, the solubility ratio between *T* = 323.15 K and *T* = 298.15 K should indicate the necessity of a temperature-dependent interaction parameter. Ratio lower than (greater than) 1.5 increases (decreases) the probability of using two such parameters (one parameter).

### AA with non-polar substituents

**Fig. 2:**
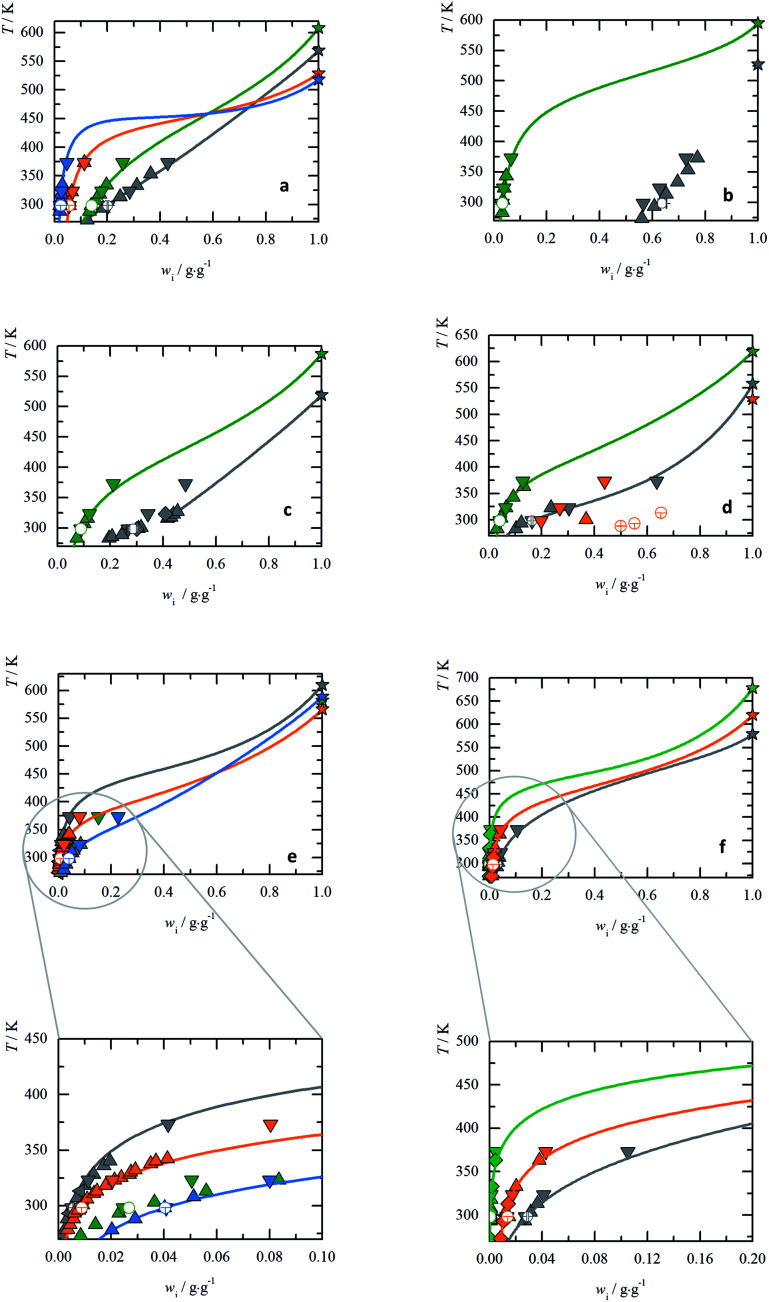
The temperature-dependent solubilities of AA: triangles represent literature data; empty circles represent the solubility measurements in present study; lines represents PC-SAFT predictions. (a) AA with non-polar substituents: **Gly**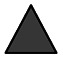: Lundblad,^36^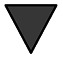 Amend,^9^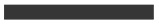: PC-SAFT. **Ala**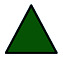: Daldrup^11^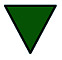: Amend,^9^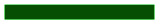: PC-SAFT. **Val**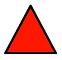: Lundblad,^36^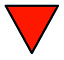 Amend,^9^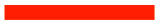: PC-SAFT. **Leu**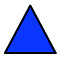: Daldrup,^11^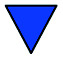: Amend,^9^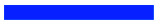: PC-SAFT. (b) AA with non-polar substituents: **Ile**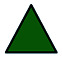: Zumstein,^38^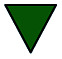: Amend^9^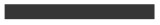: PC-SAFT. **Pro**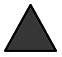: Lundblad,^36^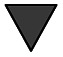: Amend.^9^ No PC-SAFT modeling due to a crystal change (ESI Fig. S26†). (c) AA with polar substituents: **Thr**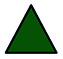: Lundblad,^36^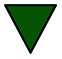: Amend,^9^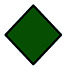: Ferreira,^43^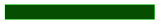: PC-SAFT. **Ser**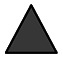: Luk^12^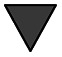: Amend,^9^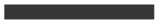: PC-SAFT. (d) AA with basic substituents: **His**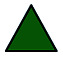: Kustov,^32^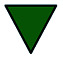: Amend,^9^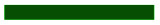: PC-SAFT. **Arg**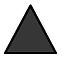: Yalkowsky,^37^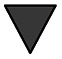: Amend,^9^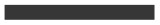: PC-SAFT. **Lys**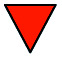: Amend.^9^ No PC-SAFT modeling due to a crystal change (ESI Fig. S32†). (e) AA with acidic substituents: **Asn**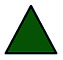: Dalton,^7^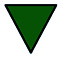: Amend.^9^ No PC-SAFT modeling due to the crystal structure change (ESI Fig. S29†) **Asp**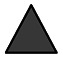: Apelblat,^40^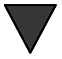: Amend,^9^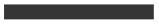: PC-SAFT. **Gln**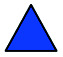: Yu,^42^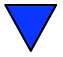: Amend,^9^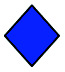: Yalkowsky,^37^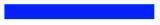: PC-SAFT. **Glu**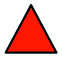: Matsuo,^41^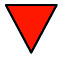: Amend,^9^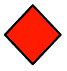: Yalkowsky,^37^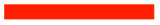: PC-SAFT. (f) AA with aromatic substituents: **Phe**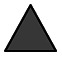: Dalton,^7^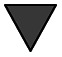: Amend,^9^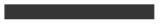: PC-SAFT. **Tyr**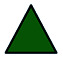: Yalkowsky,^37^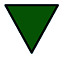: Amend,^9^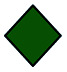: Lundblad,^36^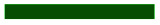: PC-SAFT. **Trp**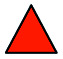: Lundblnd,^36^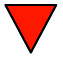: Amend,^9^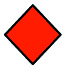: Dalton,^7^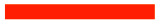: PC-SAFT.

From Fig. 2(a) it was observed that the solubility decreases in the following order Gly > Ala > Val > Leu for *T* < 450 K. However at higher temperatures, this order is disarranged. This new finding becomes possible only due to the availability of the new experimental melting data from FSC in this work. All non-polar aliphatic AA show a high tendency for sublimation/evaporation after the melting, so no glass transition step could be measured. However, even small values for differences of heat capacities moderately influence the slope of the solubility line. Therefore, heat capacity differences were fit to experimental solubility-temperature curves.

The aqueous AA solubility of Ile and Pro are shown in Fig. 2(b). Apparently Pro is most soluble in water among the twenty proteinogenic AA. In this case the PXRD results from the present work showed a change in the crystal structure which was referenced to the formation of a hydrate. The exact hydration is at least below *T*_Hydration_ ≤ 298.15. As the melting properties belong to the anhydrous form, eqn (1) cannot be applied.

### AA with polar substituents

Fig. 2(c) shows that solubility of Ser is higher than of Thr. For Thr the melting properties were taken as measured and the solubility prediction is in good agreement with the literature. For Ser a crystal change was found during the solubility measurement. The crystal change can be referenced to Luk,^12^ which shows the formation of a hydrate (*T*_Hydration_ < 312.15 K). At higher temperatures the anhydrous Ser was formed (confirmed by PXRD), which allows the application of eqn (1). This might explain the slight kink in the solubility curve observed for Ser in Fig. 2(c). The melting properties can only be determined for the anhydrous form. For the PC-SAFT predictions melting temperature and difference in heat capacity was taken from the FSC measurements. The melting enthalpy was adjusted within FSC uncertainty to the only available experimental solubility value at *T* = 315.15 K. A good agreement between PC-SAFT and experimental solubility-temperature data supports the proposed procedure.

### AA with acidic substituents

The acid AA (Asp, Glu) are characterized by a carboxyl group in the side chain and the amides (Asn, Gln) have a primary amide group. These additional polar groups also affect the pH value of the saturated solutions, which corresponds to their isoelectric points (pI). In general, these four AA show very low solubility in water, the amide AA are slightly more soluble than their acidic pendants at their pI (Fig. 2(e)). However, for Asn a hydrate has formed upon equilibration in water.^7,9^ Unfortunately, solubility literature data of the anhydrous Asn was not available. Hence eqn (1) could not be applied for temperatures below *T*_Hydration_ ≤ 298.15 and Asn solubility cannot be predicted. For Glu, Gln and Asp eqn (1) was applied and the results are in good agreement with the literature.

### AA with basic substituents

His, Arg and Lys and increase the pH value in unbuffered aqueous solution, resulting in high pI values (Table 3). For Lys the experimental solubility re-measured in this work was higher than the only available literature data,^9^ see Fig. 2(d). The PXRD diffractograms might hint Lys-hydrate formation in aqueous solutions. Williams *et al.*,^50^ observed Lys monohydrate, depending on the relative humidity level to which Lys was exposed. The anhydrous form can only be attained after vacuum drying. Thus, eqn (1) was not applied to Lys. The surprisingly low values for the melting properties (*T*^SL^_0,Lys_ = 529 ± 9 K, Δ*h*^SL^_0,Lys_ = 22 ± 3 K) indicate possible high solubility of Lys.

For His and Arg no change in crystal structure was detected and the conventional approach was applied. The solubility prediction is in good agreement with the literature data.

### AA with aromatic substituents

The aqueous solubility of the aromatic AA are very low (Fig. 2(f)) with order of Phe > Trp > Tyr. The solubility measurements from this work are in good agreement with the literature data. No crystal structure change is detected in the PXRD in aqueous solutions, which allows modeling by application of eqn (1).

Due to high sublimation/evaporation, the glass transition of Phe was unattainable, subsequently the heat capacity difference could not be determined. The heat capacity was estimated to be Δ*c*^SL^_p0i_(*T*^SL^_0i_) = 184.37 J mol^−1^ K^−1^ in order to maintain the FSC determined Δ*h*^SL^_0i_ within its experimental uncertainty. Modeling solubility without taking into account of Δ*c*^SL^_p0i_ would predict a very low Δ*h*^SL^_0i_, which is inconsistent with FSC data. This shows that the heat capacity difference is a very important property, which is unfortunately often neglected in thermodynamic modeling.

For Trp and Tyr the experimental melting properties applied in PC-SAFT are within the uncertainties of the FSC measurement. The predicted solubility of Phe, Trp and Tyr are in good agreement with the experimental solubility data.

### AA with sulfuric substituents

The solubility order for sulfuric AA is Cys > Met (ESI Fig. S35†). A crystal structure change for Cys during the measurement was observed. Hence, solubility modeling with eqn (1) was not performed.

The experimental solubility for Met is consistent with the literature data.^51^ Unfortunately no melting properties could be measured using FSC. Thus solubility modeling is also not possible. No crystal change was observed for both Cys and Met.

### Comparison to literature

The classical way of thermodynamic solubility model for components with inaccessible experimental melting properties are performed as follows: different *g*^E^ models or equations of state were used to calculate the activity coefficients for eqn (1), while simultaneously fitting the melting properties to experimental solubility data. This procedure is still state-of-the art in the literature; however, the results of this approach differ strongly from the FSC-determined melting properties. Additionally, often applied in the literature solubility model differs from the eqn (1) used in this work. For example, the modified Apelblat equation9
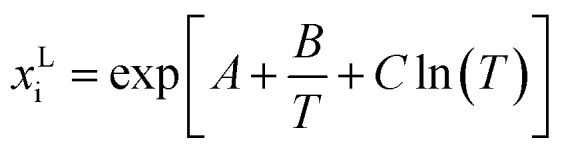
which fits the solubility with three independent parameters *A*, *B* and *C*. In this case it is not possible to distinguish the proper melting properties and therefore the comparison to the FSC melting properties is not possible. For this reason the “right side” of each solubility model can be treated as the solubility product *K*_SP_, which consist of the solubility *x*^L^_i_ and activity coefficient *γ*^L^_i_.

10 *x*^L^_i_ × *γ*^L^_i_ = *K*_SP_ = *f*(*T*)

**Fig. 3:**
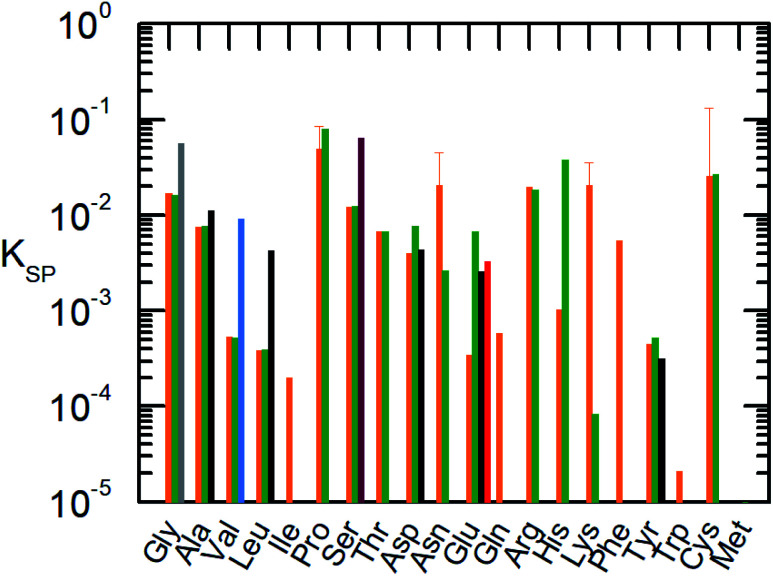
Solubility product of the AA at 298.15 K. 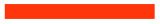: this work using eqn (1) (orange). 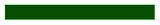: Held, 2011 (ref. 10) using eqn (1). 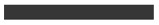: Cao *et al.*, 2013 (ref. 52) using Apelblat eqn (9). 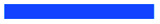: Zhang, 2014 (ref. 53) using Apelblat eqn (9), 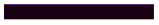: Fan, 2019:^54^ using Apelblat eqn (9), 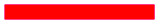: Marrero and Gani, 2001:^55^ using group contribution. For Pro, Asn, Lys and Cys the uncertainty at *T* = 298.15 K based on the FSC measurements of melting properties is shown since a crystal change was detected during the solubility measurements.

*K*_SP_ depends only on the absolute temperature *T*. This allows the comparison of different solubility models without accounting for the fitting to physically meaningful melting properties or purely adjustable fitting parameters (Fig. 3).

In Fig. 3 the solubility product of each AA is shown at *T* = 298.15 K and *T* = 323.15 K. In some literature studies the melting properties were calculated by using group contribution methods without further applying it on solubility modeling.^37,55^ In this case, we applied eqn (1). However, regardless of how the melting properties/adjustable parameter was achieved, it is clear that the literature data differ to the solubility product determined in the current work. The predicted solubility based on the experimental melting properties is in good agreement with the experimental solubility, therefore the solubility product is more precise in comparison to other models in literature.

## Conclusions

In this work nineteen proteinogenic AA (except Met) were characterized using FSC and the melting properties were successfully determined. It was shown that the experimentally determined melting properties are indispensable parts of solubility predictions using PC-SAFT. The access to the melting properties not only allows solubility prediction but also the quantification of the activity coefficients, which will give access to future model validation. The combination of FSC and PC-SAFT opens the door to predict solubility of solid compounds that decompose before melting.

## Funding

The authors acknowledge funding from German Research Foundation (DFG) with Grants HE 7165/6-1 and CH 1922/1-1.

## Conflicts of interest

There are no conflicts to declare.

## Supplementary Material

RA-010-D0RA08947H-s001
