# Supplementary material for: Melting properties of amino acids and their solubility in water
Source: RSC Adv. 2020 Dec 15;10(72):44205–15. doi: 10.1039/d0ra08947h (PMC9058464; doi:10.1039/d0ra08947h)
Supplement: RA-010-D0RA08947H-s001 [file RA-010-D0RA08947H-s001.pdf]

Please do not adjust margins

ARTICLE

Melting properties of amino acids and their solubility in water

Hoang Tam Do[a‡], Yeong Zen Chua[b,c‡], Aarti Kumar[a], Daniel Pabsch[a], Moritz Hallermann[a], Dzmitry Zaitsau[c,d], Christoph Schick[b,c,e], Christoph Held[a]

Received 00th January 20xx,
Accepted 00th January 20xx

DOI: 10.1039/x0xx00000x

The state-of-the-art unit operation for separation and purification of amino acids is still the crystallization, which requires solubility data and melting properties of pure compounds. Since measuring solubility is time-consuming, prediction tools are desired. Further, melting properties are not yet available due to decomposition of amino acids upon slow heating. In this work, melting properties of twenty amino acids (except Met) were measured by Fast Scanning Calorimetry (FSC) with heating rates up to 20,000 K·s-1. PC-SAFT was used to predict interactions in amino acid + water systems. Additionally, solubility, pH, and PXRD was measured. By combining FSC and PC-SAFT, solubility of 15 amino acids was successfully predicted in a wide temperature range in good agreement to the experimental data. Thus, this work provides melting properties of amino acids for the first time and highlights the usefulness of such data to predict material properties such as aqueous solubility of amino acids.

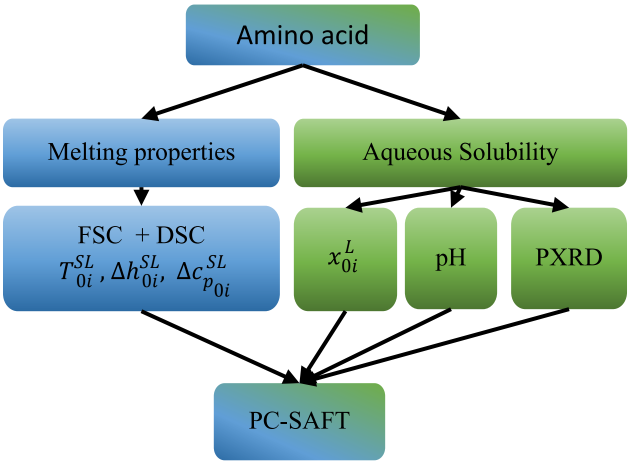

Figure S1 Working flowsheet of this work in the cooperation of University of Rostock (blue) and TU Dortmund (green).

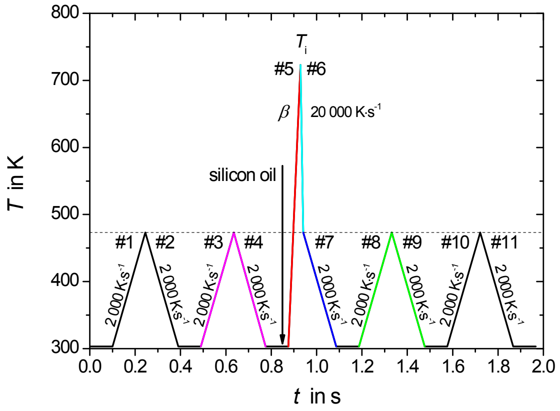

Figure S2 Temperature-time profile for determination of melting properties with fast scanning calorimetry. In heating step #5, the scanning rate, β, varied from 1 000 K·s^{-1} to 20 000 K·s^{-1}.

[a]Laboratory of Thermodynamics, TU Dortmund University, Emil-Figge-Str. 70, 44227 Dortmund, Germany. E-mail: christoph.held@tu-dortmund.de

[b]Institute of Physics, University of Rostock, Albert-Einstein-Str. 23-24, 18051 Rostock, Germany. E-mail: yeong.chua@uni-rostock.de

[c]Competence Centre CALOR, University of Rostock, Albert-Einstein-Str. 25, 18051 Rostock, Germany

[b]Institute of Chemistry, University of Rostock, Dr-Lorenz-Weg 2, 18051 Rostock, Germany

[b]Chemical Institute A. M. Butlerov, Kazan Federal University, 18 Kremlyovskaya Street, Kazan 420008, Russian Federation

[†] Electronic supplementary information (ESI) available. See DOI:

[‡] Shared first authors.

[*] E-mail: christoph.held@tu-dortmund.de (solubility)
E-mail: yeong.chua@uni-rostock.de (FSC)

Please do not adjust margins

Please do not adjust margins

Heat capacity of pure amino acids

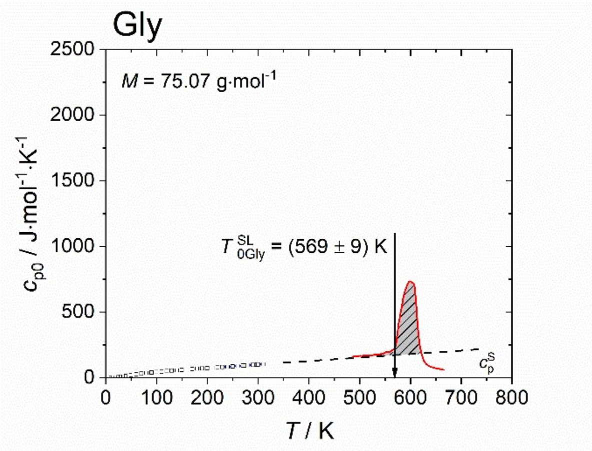

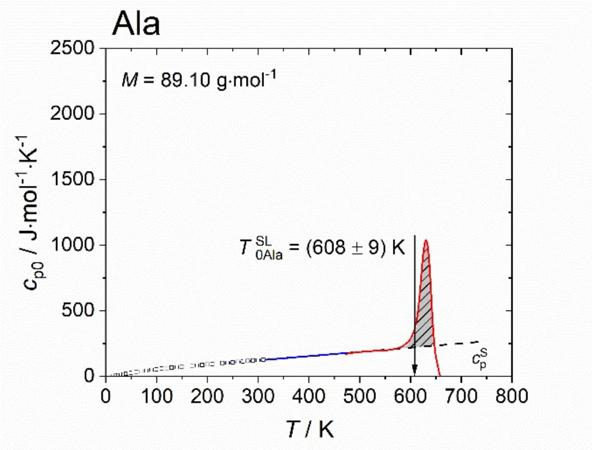

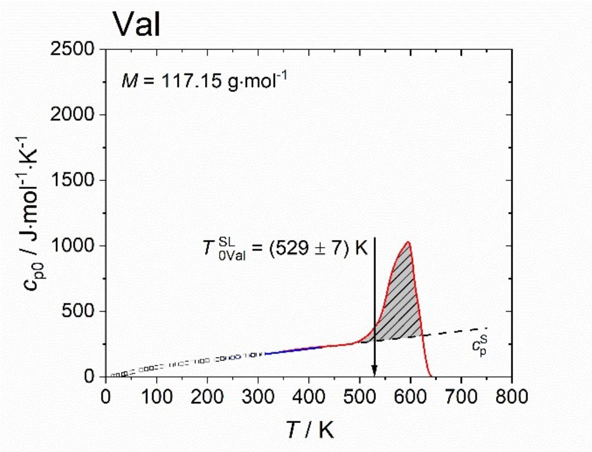

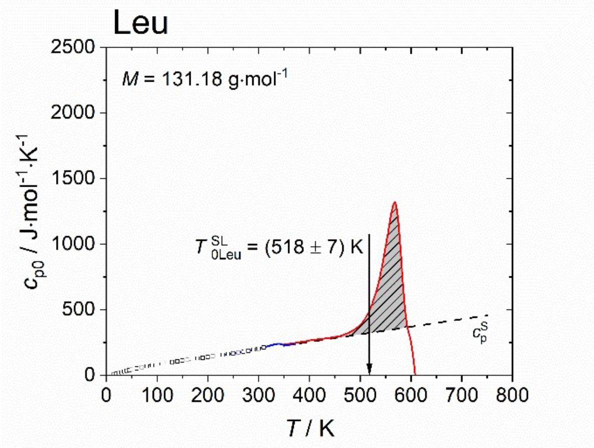

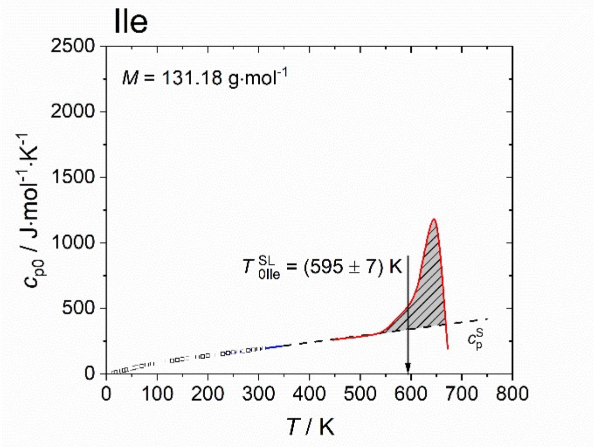

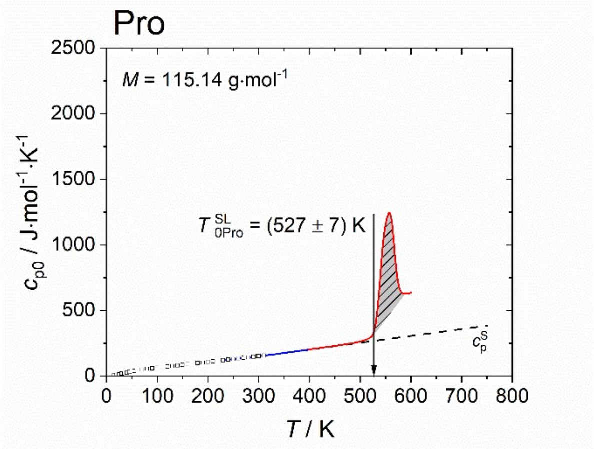

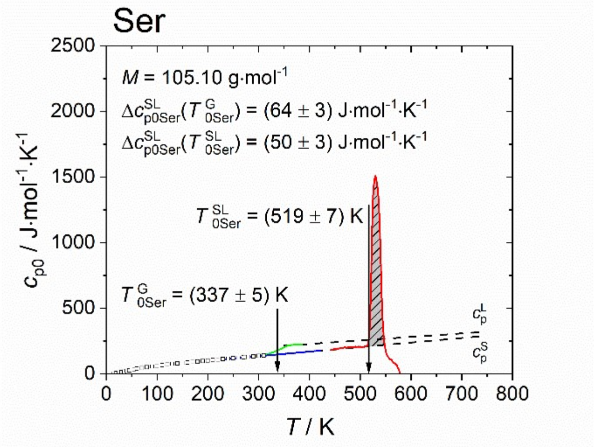

Please do not adjust margins

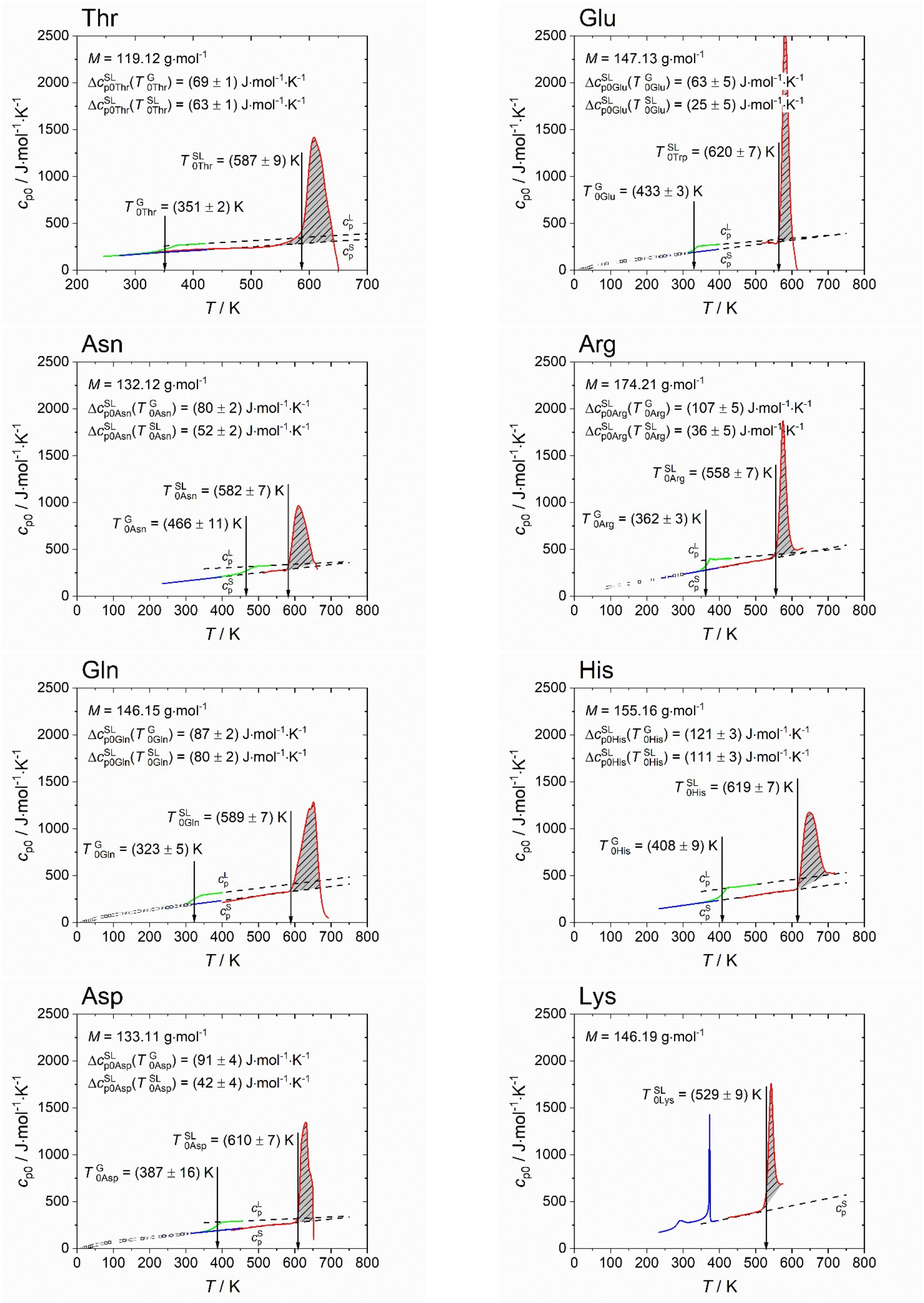
Thr
M = 119.12 g·mol-1
ΔcSLp0Thr(T G0Thr) = (69 ± 1) J·mol-1·K-1
ΔcSLp0Thr(T SL0Thr) = (63 ± 1) J·mol-1·K-1
T SL0Thr = (587 ± 9) K
T G0Thr = (351 ± 2) K
cLp
cSp
cp0 / J·mol-1·K-1
T / K
Glu
M = 147.13 g·mol-1
ΔcSLp0Glu(T G0Glu) = (63 ± 5) J·mol-1·K-1
ΔcSLp0Glu(T SL0Glu) = (25 ± 5) J·mol-1·K-1
T SL0Trp = (620 ± 7) K
T G0Glu = (433 ± 3) K
Asn
M = 132.12 g·mol-1
ΔcSLp0Asn(T G0Asn) = (80 ± 2) J·mol-1·K-1
ΔcSLp0Asn(T SL0Asn) = (52 ± 2) J·mol-1·K-1
T SL0Asn = (582 ± 7) K
T G0Asn = (466 ± 11) K
Arg
M = 174.21 g·mol-1
ΔcSLp0Arg(T G0Arg) = (107 ± 5) J·mol-1·K-1
ΔcSLp0Arg(T SL0Arg) = (36 ± 5) J·mol-1·K-1
T SL0Arg = (558 ± 7) K
T G0Arg = (362 ± 3) K
Gln
M = 146.15 g·mol-1
ΔcSLp0Gln(T G0Gln) = (87 ± 2) J·mol-1·K-1
ΔcSLp0Gln(T SL0Gln) = (80 ± 2) J·mol-1·K-1
T SL0Gln = (589 ± 7) K
T G0Gln = (323 ± 5) K
His
M = 155.16 g·mol-1
ΔcSLp0His(T G0His) = (121 ± 3) J·mol-1·K-1
ΔcSLp0His(T SL0His) = (111 ± 3) J·mol-1·K-1
T SL0His = (619 ± 7) K
T G0His = (408 ± 9) K
Asp
M = 133.11 g·mol-1
ΔcSLp0Asp(T G0Asp) = (91 ± 4) J·mol-1·K-1
ΔcSLp0Asp(T SL0Asp) = (42 ± 4) J·mol-1·K-1
T SL0Asp = (610 ± 7) K
T G0Asp = (387 ± 16) K
Lys
M = 146.19 g·mol-1
T SL0Lys = (529 ± 9) K

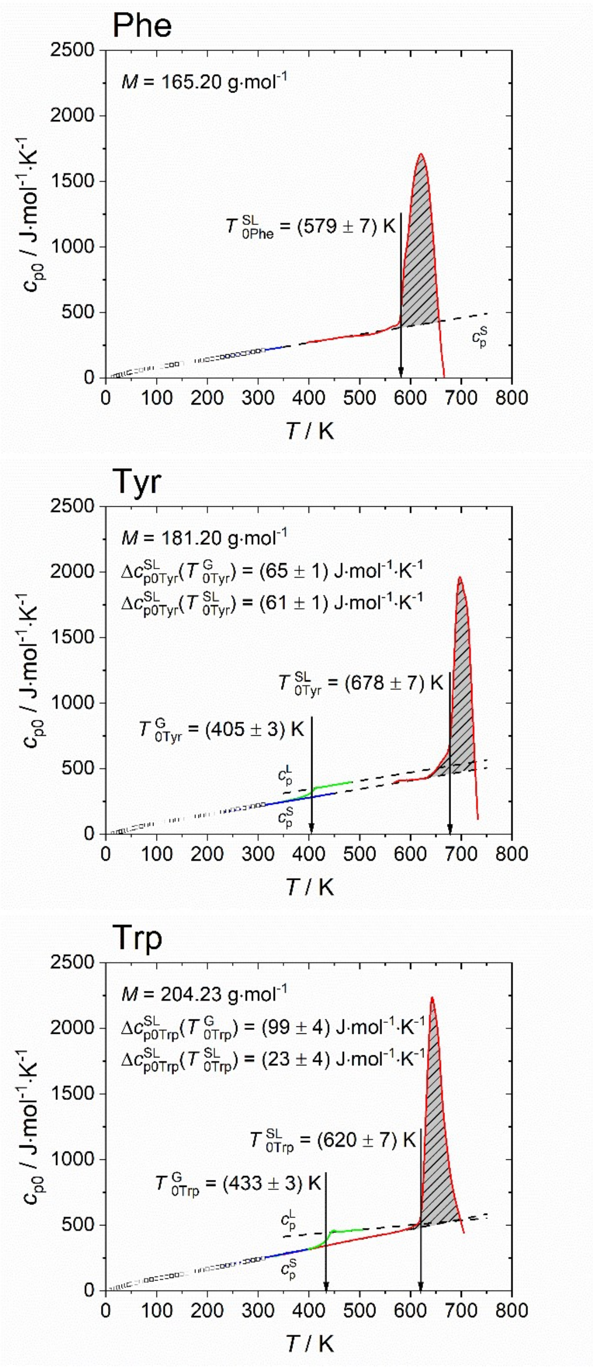

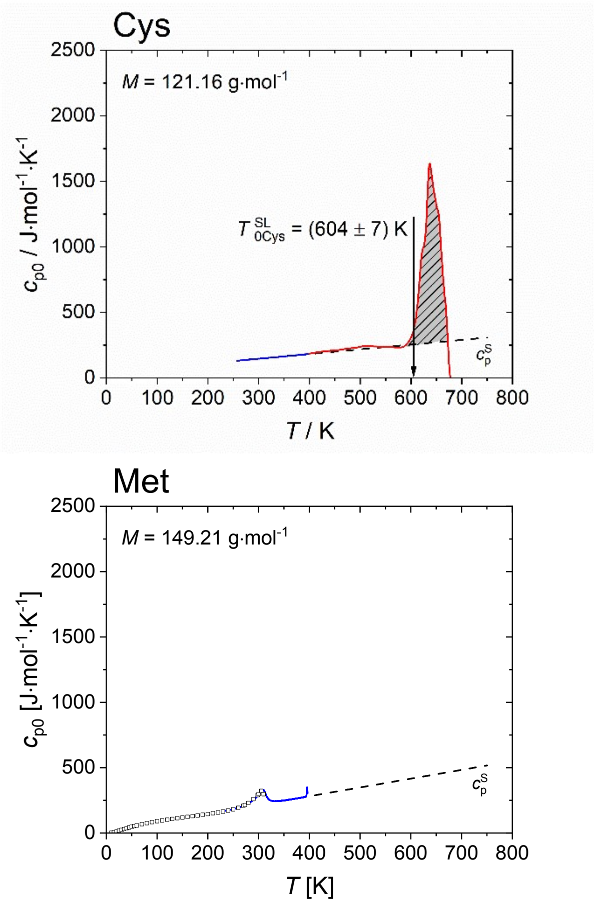

Figure S3. Specific heat capacity of 20 proteinogenic amino acids determined experimentally with FSC (solid red lines for melting peak in heating step #5 and solid green lines for glass transition step of ultra-fast quenched melted amino acids without silicon oil) and standard DSC (solid blue lines for heat capacity of solid, c_{p0i}^{S}). The melting temperature is determined as the onset temperature of the melting peak while the melting enthalpy as area under the melting peak. The heat capacity of solid c_{p0i}^{S} and liquid c_{p0i}^{L} (dashed lines respectively) were linearly fitted to T_{0i}^{SL}. The heat capacity difference between liquid and solid phase were determined at glass transition temperature, $\Delta c_{p0i}^{SL}(T_{0i}^{G})$ and extrapolation to melting temperature, $\Delta c_{p0i}^{SL}(T_{0i}^{SL})$. The empty black squares are specific heat capacity of solid from literatures (Gly,[1] Ala,[1] Val,[2] Leu,[2] Ile,[2] Pro,[3] Ser,[4] Thr, Asn, Gln,[5] Asp,[5] Glu,[5] Arg,[6] His, Lys, Phe,[3] Tyr,[3] Trp,[3] Cys, Met[7]).

Please do not adjust margins

Melting temperature and melting enthalpy of pure amino acids

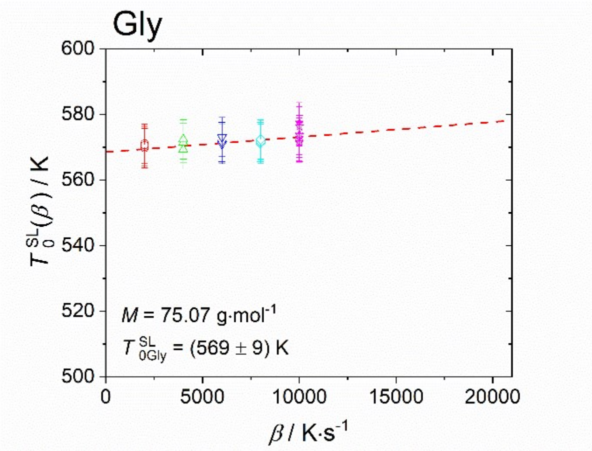

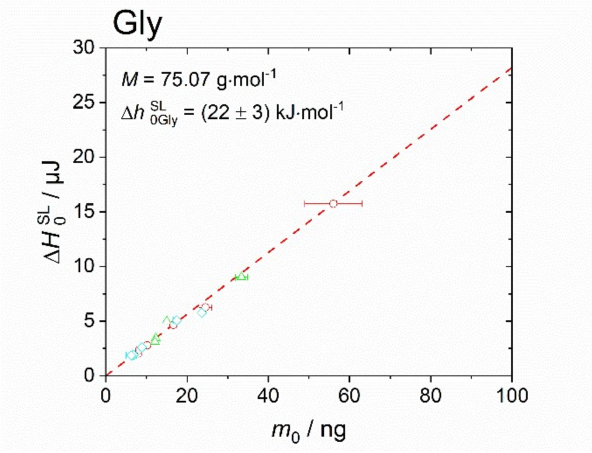

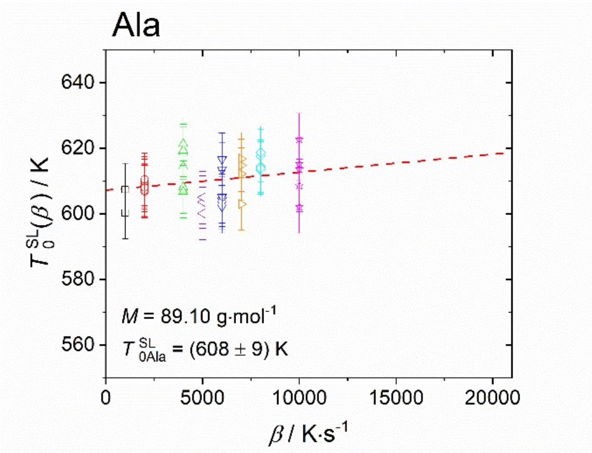

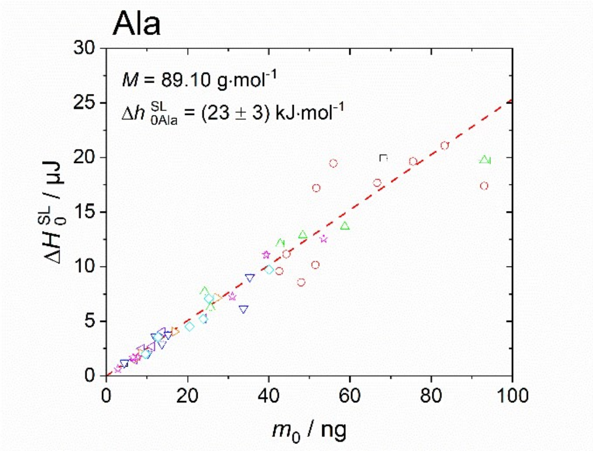

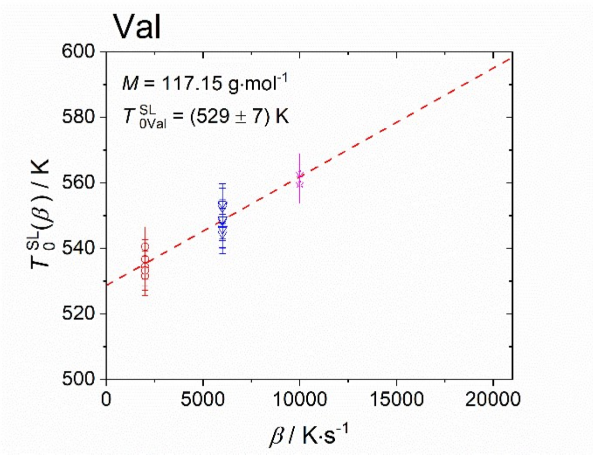

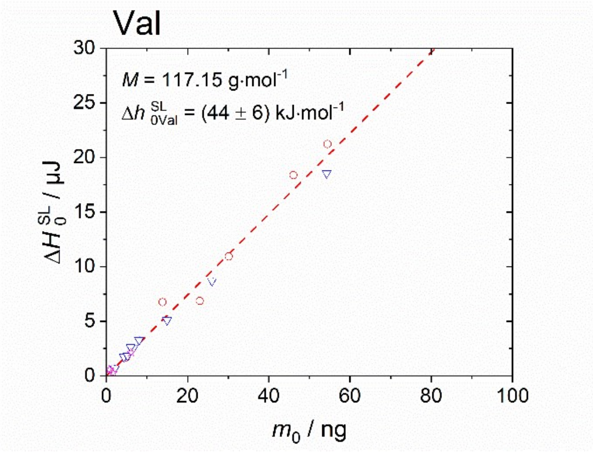

This journal is © The Royal Society of Chemistry 20xx

Please do not adjust margins

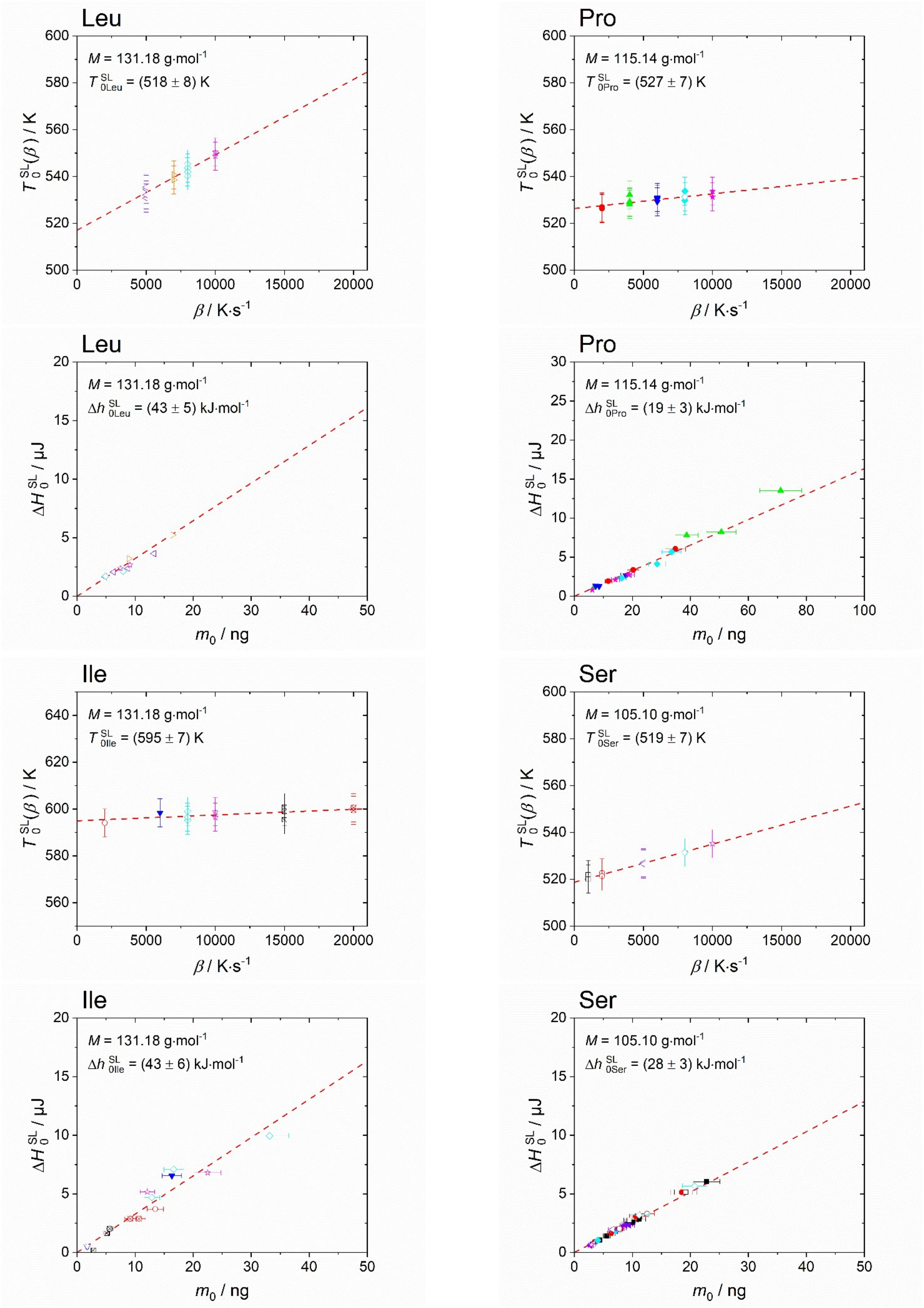
Leu
M = 131.18 g·mol-1
T SL 0Leu = (518 ± 8) K
T SL 0(β) / K
β / K·s-1
Pro
M = 115.14 g·mol-1
T SL 0Pro = (527 ± 7) K
T SL 0(β) / K
β / K·s-1
Leu
M = 131.18 g·mol-1
Δh SL 0Leu = (43 ± 5) kJ·mol-1
ΔH SL 0 / µJ
m0 / ng
Pro
M = 115.14 g·mol-1
Δh SL 0Pro = (19 ± 3) kJ·mol-1
ΔH SL 0 / µJ
m0 / ng
Ile
M = 131.18 g·mol-1
T SL 0Ile = (595 ± 7) K
T SL 0(β) / K
β / K·s-1
Ser
M = 105.10 g·mol-1
T SL 0Ser = (519 ± 7) K
T SL 0(β) / K
β / K·s-1
Ile
M = 131.18 g·mol-1
Δh SL 0Ile = (43 ± 6) kJ·mol-1
ΔH SL 0 / µJ
m0 / ng
Ser
M = 105.10 g·mol-1
Δh SL 0Ser = (28 ± 3) kJ·mol-1
ΔH SL 0 / µJ
m0 / ng

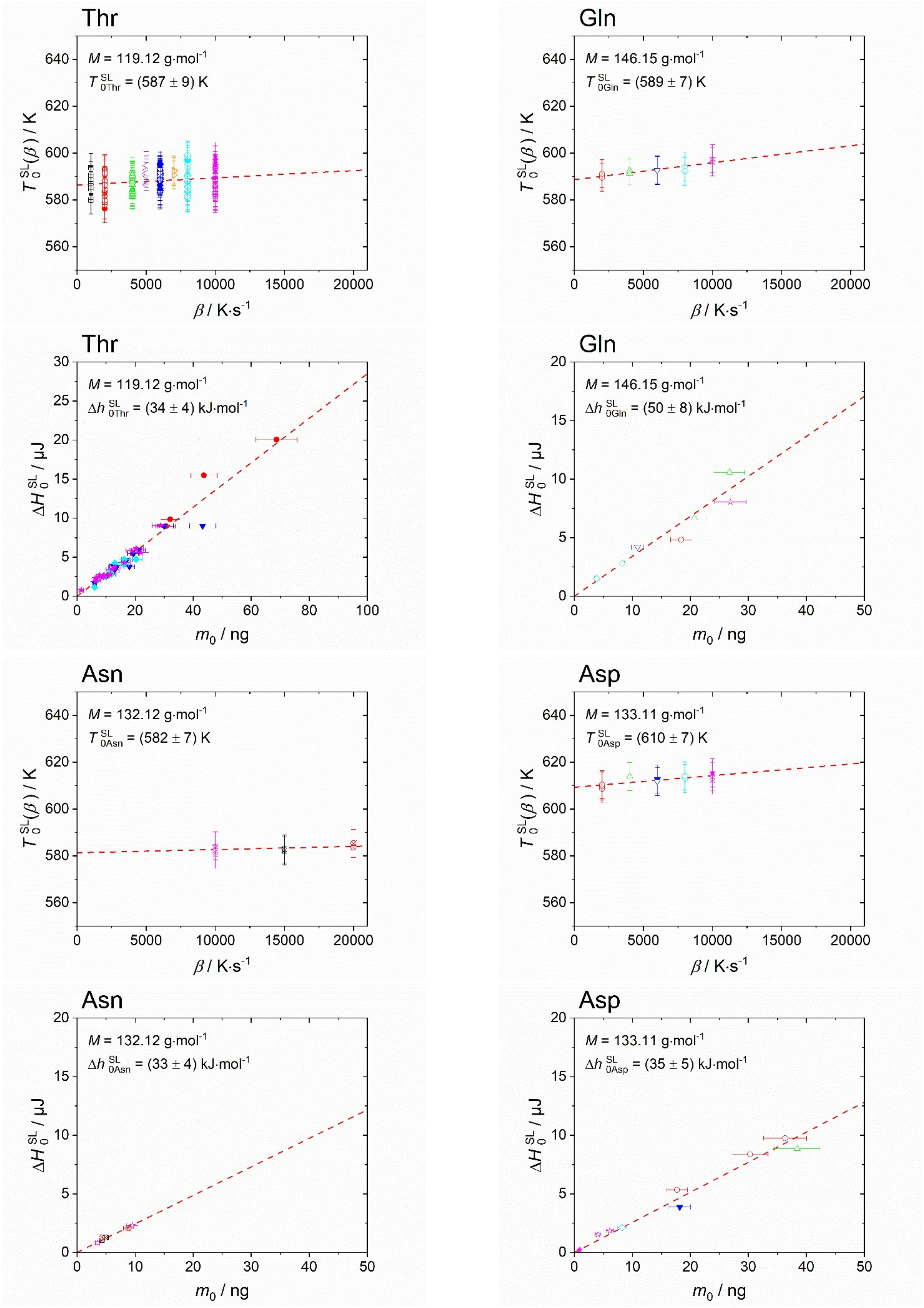
Thr
M = 119.12 g·mol-1
T SL 0Thr = (587 ± 9) K
T SL 0(β) / K
β / K·s-1
Gln
M = 146.15 g·mol-1
T SL 0Gln = (589 ± 7) K
T SL 0(β) / K
β / K·s-1
Thr
M = 119.12 g·mol-1
Δh SL 0Thr = (34 ± 4) kJ·mol-1
ΔH SL 0 / μJ
m0 / ng
Gln
M = 146.15 g·mol-1
Δh SL 0Gln = (50 ± 8) kJ·mol-1
ΔH SL 0 / μJ
m0 / ng
Asn
M = 132.12 g·mol-1
T SL 0Asn = (582 ± 7) K
T SL 0(β) / K
β / K·s-1
Asp
M = 133.11 g·mol-1
T SL 0Asp = (610 ± 7) K
T SL 0(β) / K
β / K·s-1
Asn
M = 132.12 g·mol-1
Δh SL 0Asn = (33 ± 4) kJ·mol-1
ΔH SL 0 / μJ
m0 / ng
Asp
M = 133.11 g·mol-1
Δh SL 0Asp = (35 ± 5) kJ·mol-1
ΔH SL 0 / μJ
m0 / ng

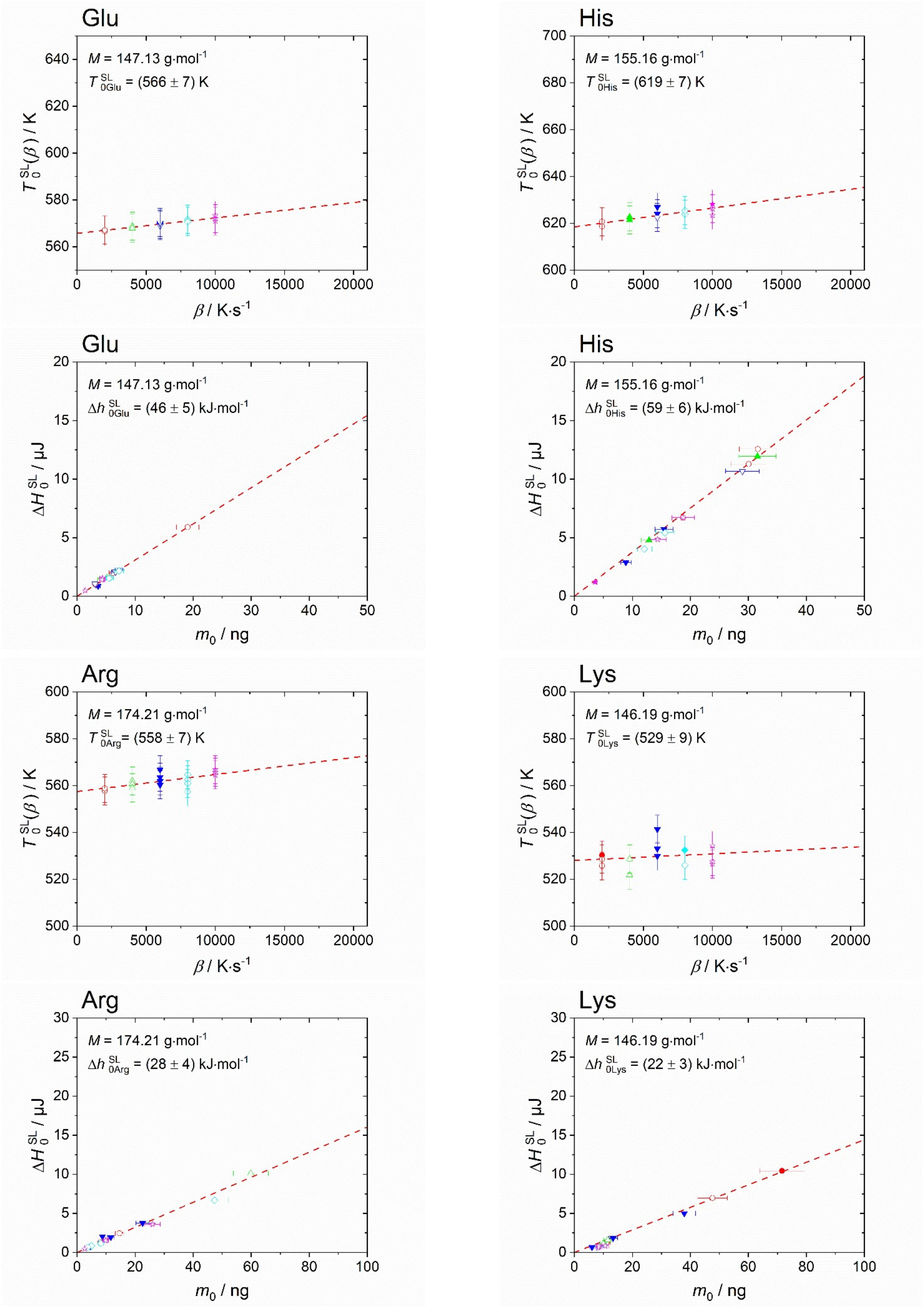
Glu
M = 147.13 g·mol-1
T SL 0Glu = (566 ± 7) K
T SL 0(β) / K
β / K·s-1
Glu
M = 147.13 g·mol-1
Δh SL 0Glu = (46 ± 5) kJ·mol-1
ΔH SL 0 / μJ
m0 / ng
His
M = 155.16 g·mol-1
T SL 0His = (619 ± 7) K
T SL 0(β) / K
β / K·s-1
His
M = 155.16 g·mol-1
Δh SL 0His = (59 ± 6) kJ·mol-1
ΔH SL 0 / μJ
m0 / ng
Arg
M = 174.21 g·mol-1
T SL 0Arg = (558 ± 7) K
T SL 0(β) / K
β / K·s-1
Arg
M = 174.21 g·mol-1
Δh SL 0Arg = (28 ± 4) kJ·mol-1
ΔH SL 0 / μJ
m0 / ng
Lys
M = 146.19 g·mol-1
T SL 0Lys = (529 ± 9) K
T SL 0(β) / K
β / K·s-1
Lys
M = 146.19 g·mol-1
Δh SL 0Lys = (22 ± 3) kJ·mol-1
ΔH SL 0 / μJ
m0 / ng

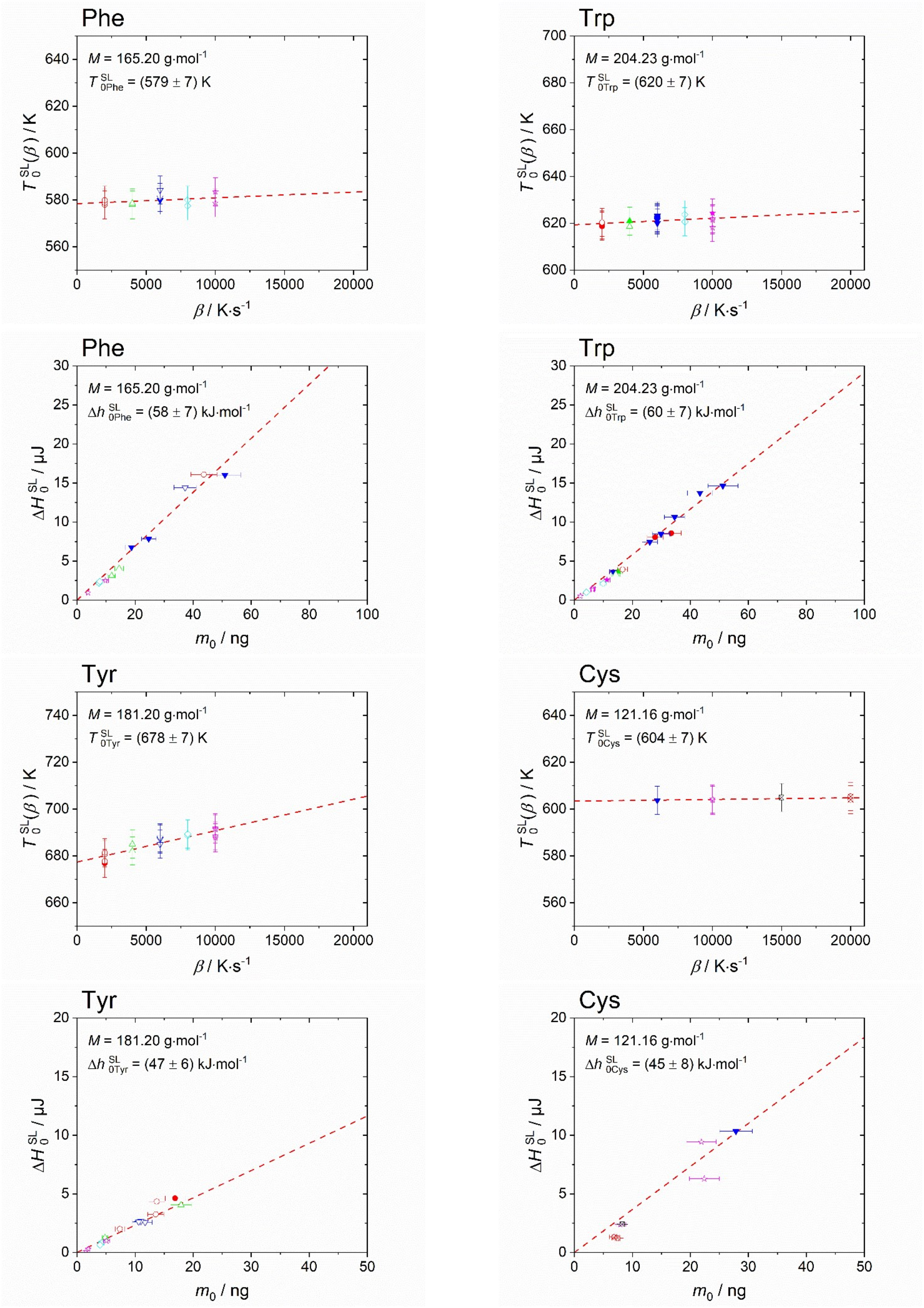
Phe
M = 165.20 g·mol^{-1}
T^{SL}_{0Phe} = (579 ± 7) K
$T^{SL}_0(\beta)$ / K
β / K·s^{-1}
Trp
M = 204.23 g·mol^{-1}
T^{SL}_{0Trp} = (620 ± 7) K
$T^{SL}_0(\beta)$ / K
β / K·s^{-1}
Phe
M = 165.20 g·mol^{-1}
Δh^{SL}_{0Phe} = (58 ± 7) kJ·mol^{-1}
ΔH^{SL}_0 / µJ
m_0 / ng
Trp
M = 204.23 g·mol^{-1}
Δh^{SL}_{0Trp} = (60 ± 7) kJ·mol^{-1}
ΔH^{SL}_0 / µJ
m_0 / ng
Tyr
M = 181.20 g·mol^{-1}
T^{SL}_{0Tyr} = (678 ± 7) K
$T^{SL}_0(\beta)$ / K
β / K·s^{-1}
Cys
M = 121.16 g·mol^{-1}
T^{SL}_{0Cys} = (604 ± 7) K
$T^{SL}_0(\beta)$ / K
β / K·s^{-1}
Tyr
M = 181.20 g·mol^{-1}
Δh^{SL}_{0Tyr} = (47 ± 6) kJ·mol^{-1}
ΔH^{SL}_0 / µJ
m_0 / ng
Cys
M = 121.16 g·mol^{-1}
Δh^{SL}_{0Cys} = (45 ± 8) kJ·mol^{-1}
ΔH^{SL}_0 / µJ
m_0 / ng

Please do not adjust margins

Figure S4 Melting properties of 19 proteinogenic amino acids (except for Met) determined from Figure S3. **Left**: Onset temperature of the melting peak of amino acids in heating step #5, as function of scanning rates. The dashed line denotes linear extrapolation of melting temperatures to zero scanning rates $\Delta T_{0i}^{SL}(\beta \to 0)$ The scanning rates used were 1000 K·s^{-1} (black squares), 2000 K·s^{-1} (red circles), 4000 K·s^{-1} (green up-triangles), 5000 K·s^{-1} (violet left-triangles), 6000 K·s^{-1} (blue down-triangles), 7000 K·s^{-1} (orange right-triangles), 8000 K·s^{-1} (cyan diamonds), 10000 K·s^{-1} (magenta stars), 15000 K·s^{-1} (black cross squares) and 20000 K·s^{-1} (red cross circles). Solid symbols represent measurements without silicon oil, while empty symbols for measurements with silicon oil. **Right:** Enthalpy, ΔH_{0i}^{SL}, of amino acids in respect to sample mass, m_0, regardless of the scanning rates. The slope of the linear fit through zero origin (dashed red line) signifies the melting enthalpy, Δh_{0i}^{SL}. The T_{0i}^{SL}, Δh_{0i}^{SL}, $\Delta c_{p0i}^{SL}(T_{0i}^{G})$ and $\Delta c_{p0i}^{SL}(T_{0i}^{SL})$ for each amino acids are listed in Table 2.

This journal is © The Royal Society of Chemistry 20xx

Please do not adjust margins

Please do not adjust margins

PC-SAFT parameters of pure amino acids

In this section, the results of the pure-component parameter fitting are presented. In case no literature data was already available, the experimental data was determined in this work. The methods used to determine the mixture densities as well as the osmotic coefficients of amino acids in water are already described in previous work from Held, 2011[8]

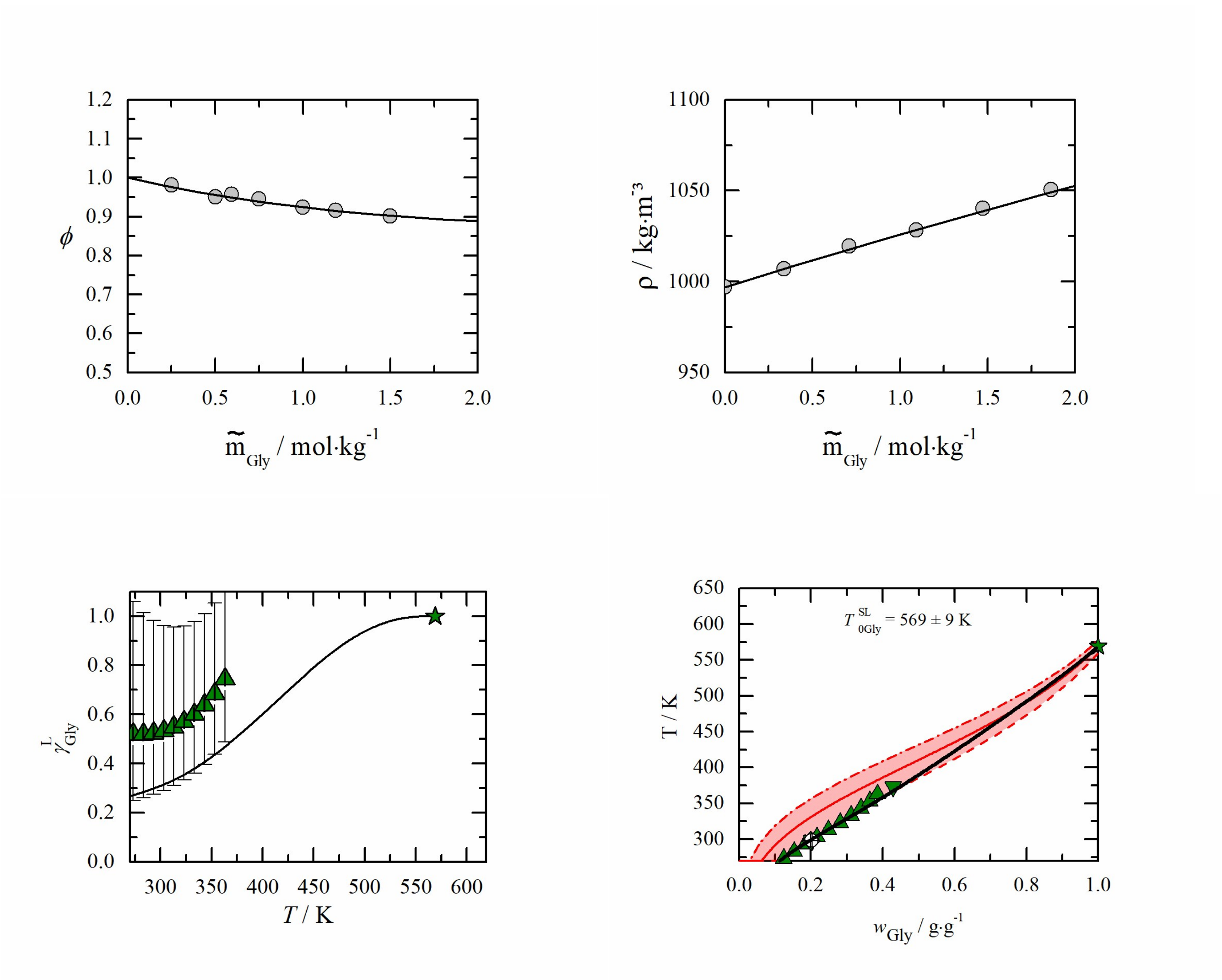

Figure S5 **Left top:** Densities of Gly + water solutions vs. molality at T = 298.15 K and p = 1 atm. Solid symbols represent experimental data Yan, 1999[9] **Right top:** Osmotic coefficients of Gly + water solutions at T = 298.15 K and p = 1 atm. Solid symbols are experimental data Held, 2011[8]. The lines are the respective PC-SAFT modeling results with parameters from Table 2. **Left bottom:** Activity coefficients vs. temperature diagram. Uncertainties are based on the uncertainties of the melting properties. Solid symbols represent experimental data**.** ▲ : Lundbland[10] **Right bottom:** Aqueous solubility as temperature vs. weight fraction diagram. The red area presents the solubility modeling assuming $\gamma_i^L = 1$ (eq. (1)) in the range of the uncertainties of the melting properties. Solid symbols represent experimental data**.** ▲ : Lundbland[10], ▼: Amend[11], The lines are the respective PC-SAFT modeling using the parameters from Table 2 and the melting properties from Table 3

Please do not adjust margins

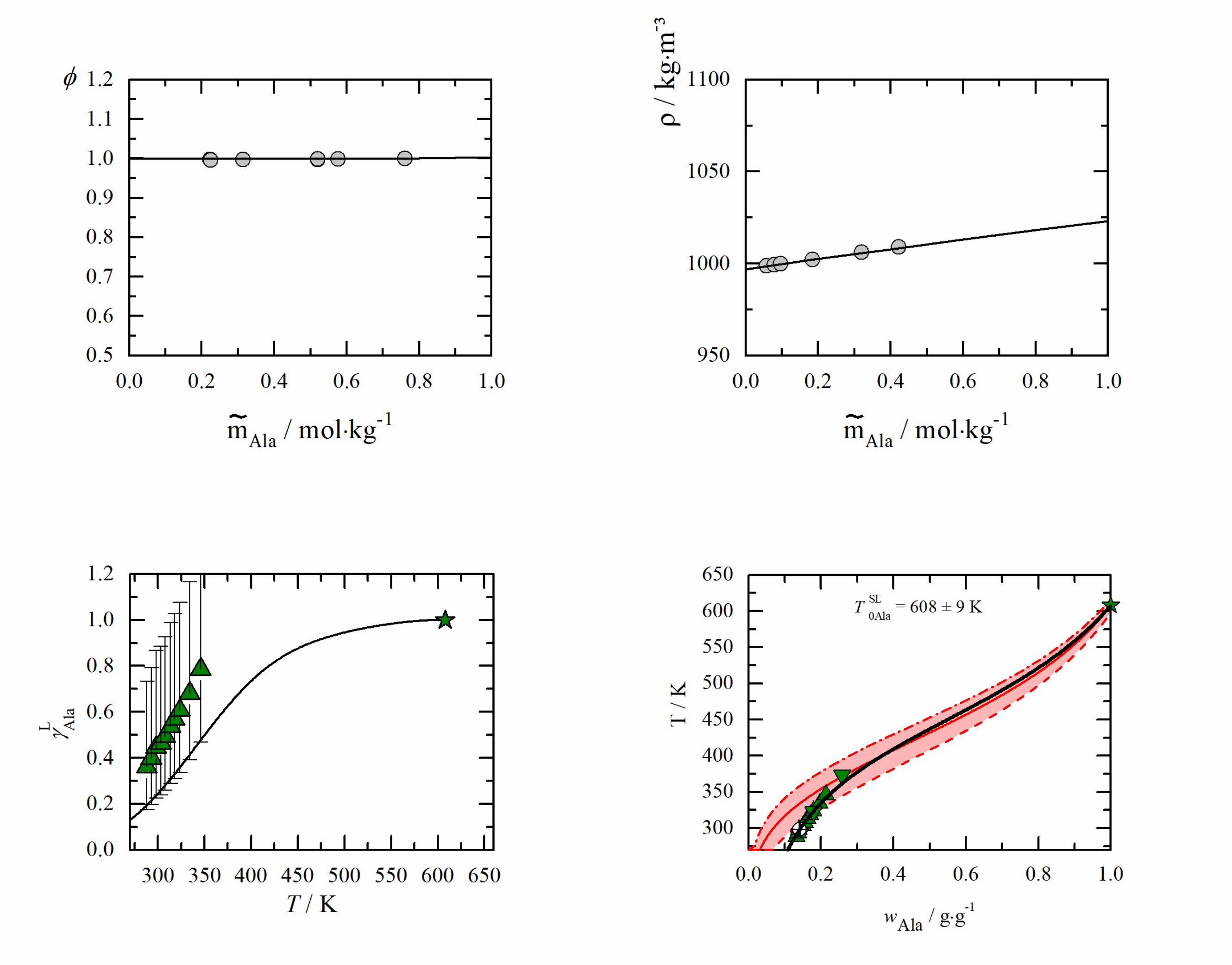

Figure S6 **Left top:** Densities of Ala + water solutions vs. molality at T = 298.15 K and p = 1 atm. Solid symbols represent experimental data Yan, 1999[9]. **Right top:** Osmotic coefficients of Ala + water solutions at T = 298.15 K and p = 1 atm. Solid symbols are experimental data Romero, 2006[12]. The lines are the respective PC-SAFT modeling results with parameters from Table 2. **Left bottom:** Activity coefficients vs. temperature diagram. Uncertainties are based on the uncertainties of the melting properties. Solid symbols represent experimental data**.** ▲ Daldrup[13] **Right bottom:** Aqueous solubility as temperature vs. weight fraction diagram. The red area presents the solubility modeling assuming $\gamma_i^L = 1$ (eq. (1)) in the range of the uncertainties of the melting properties. Solid symbols represent experimental data **Ala.** ▲: Daldrup[13] ▼: Amend[11], The lines are the respective PC-SAFT modeling using the parameters from Table 2 and the melting properties from Table 3

 This journal is © The Royal Society of Chemistry 20xx

Please do not adjust margins

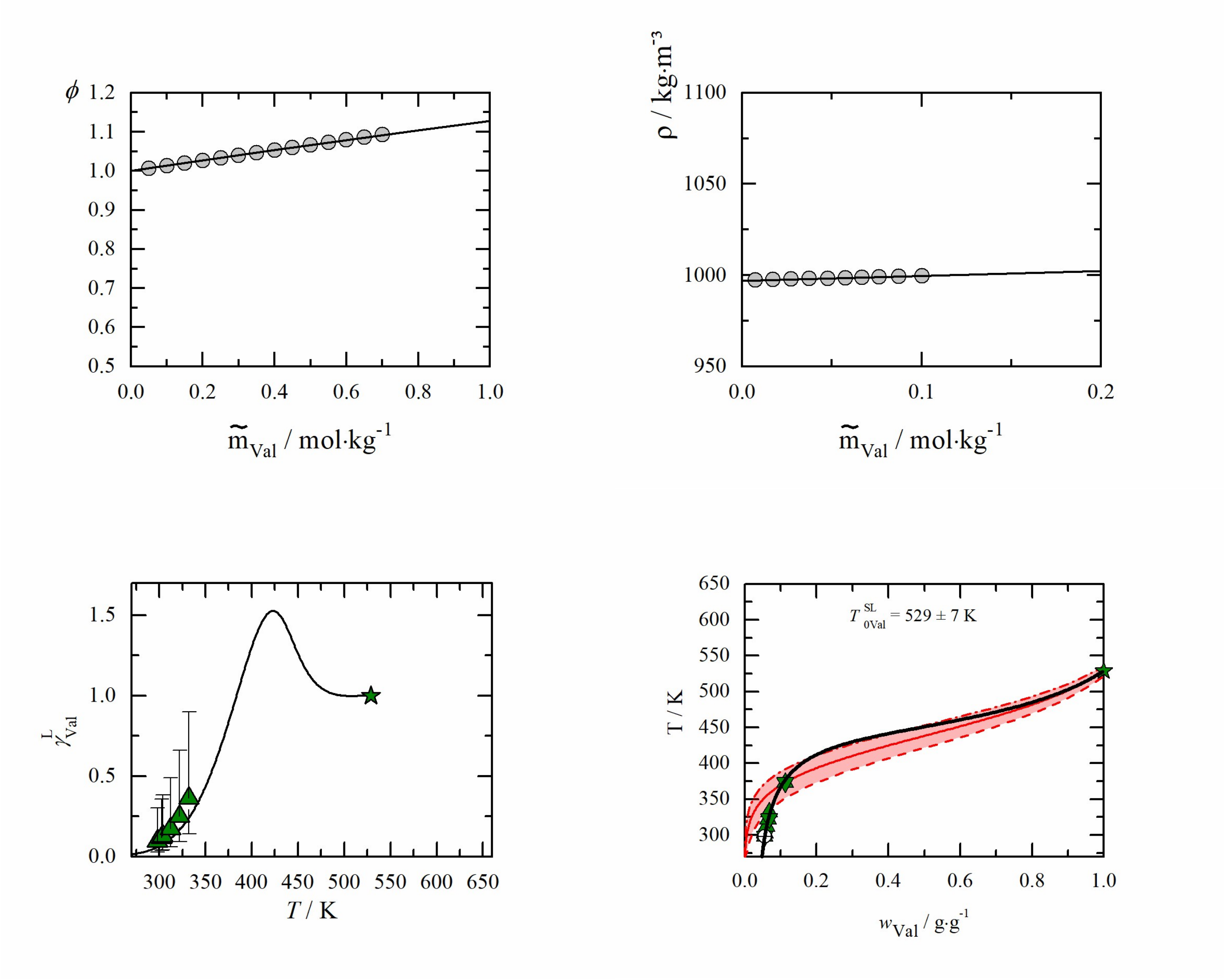

Figure S7 **Left top:** Densities of Val + water solutions vs. molality at T = 298.15 K and p = 1 atm. Solid symbols represent experimental data Kikuchi, 1995[14] **Right top:** Osmotic coefficients of Val + water solutions at T = 298.15 K and p = 1 atm. Solid symbols are experimental data Smith, 1937[15]. The lines are the respective PC-SAFT modeling results with parameters from Table 2. **Left bottom:** Activity coefficients vs. temperature diagram. Uncertainties are based on the uncertainties of the melting properties. Solid symbols represent experimental data**.** ▲: Lundbland[10], **Right bottom:** Aqueous solubility as temperature vs. weight fraction diagram. The red area presents the solubility modeling assuming $\gamma_i^L = 1$ (eq. (1)) in the range of the uncertainties of the melting properties. Solid symbols represent experimental data**.** ▲: Lundbland[10], ▼: Amend[11] The lines are the respective PC-SAFT modeling using the parameters from Table 2 and the melting properties from Table 3

Please do not adjust margins

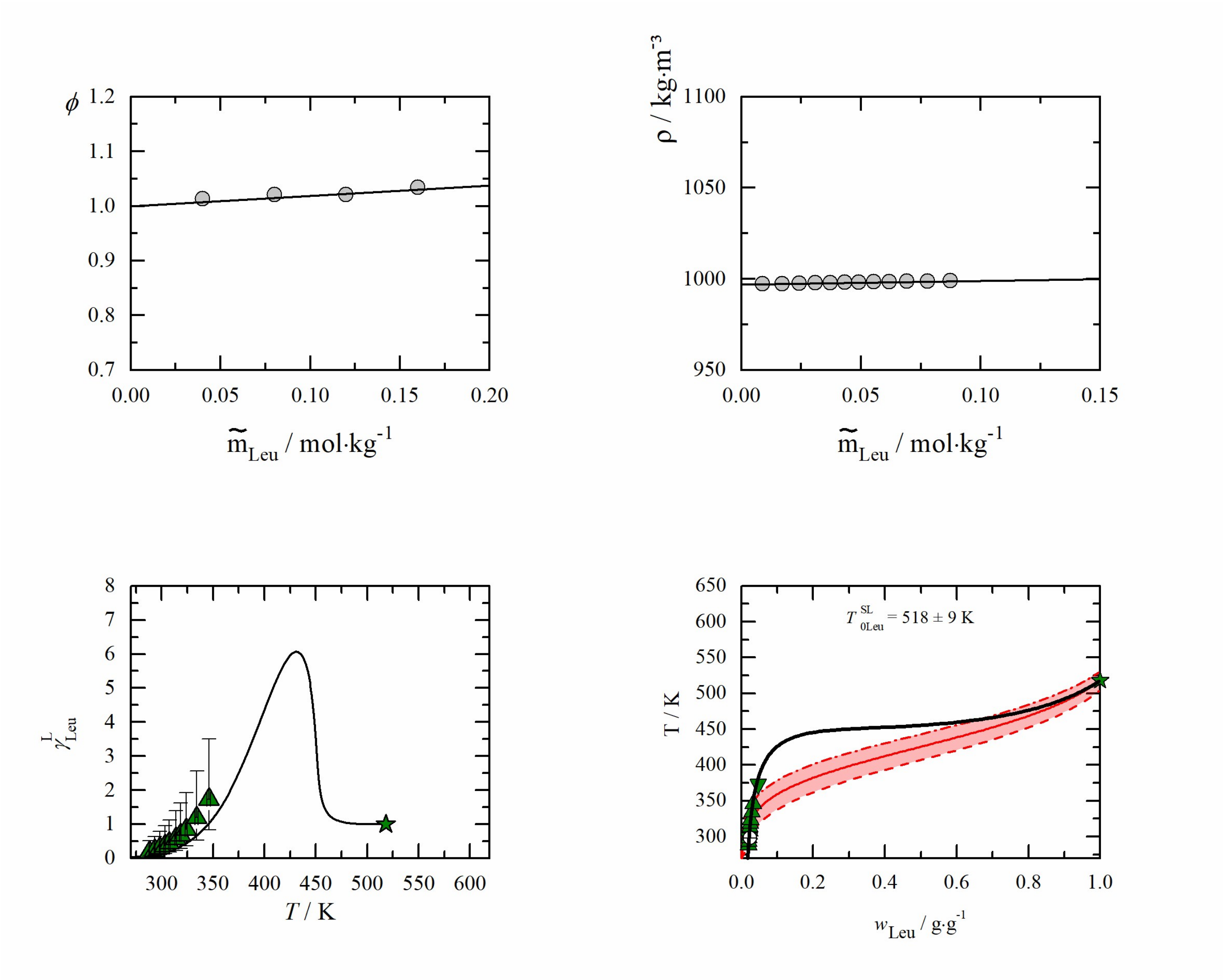

Figure S8 **Left top:** Densities of Leu + water solutions vs. molality at T = 298.15 K and p = 1 atm. Solid symbols represent experimental data Yan, 1999[9]. **Right top:** Osmotic coefficients of Leu + water solutions at T = 298.15 K and p = 1 atm. Solid symbols are experimental data Held, 2011[8]. The lines are the respective PC-SAFT modeling results with parameters from Table 2. **Left bottom:** Activity coefficients vs. temperature diagram. Uncertainties are based on the uncertainties of the melting properties. Solid symbols represent experimental data**.** ▲ Daldrup[13], **Right bottom:** Aqueous solubility as temperature vs. weight fraction diagram. The red area presents the solubility modeling assuming $\gamma_i^L = 1$ (eq. (1)) in the range of the uncertainties of the melting properties. Solid symbols represent experimental data**.** ▲ Daldrup[13], ▼: Amend[11], The lines are the respective PC-SAFT modeling using the parameters from Table 2 and the melting properties from Table 3

 This journal is © The Royal Society of Chemistry 20xx

Please do not adjust margins

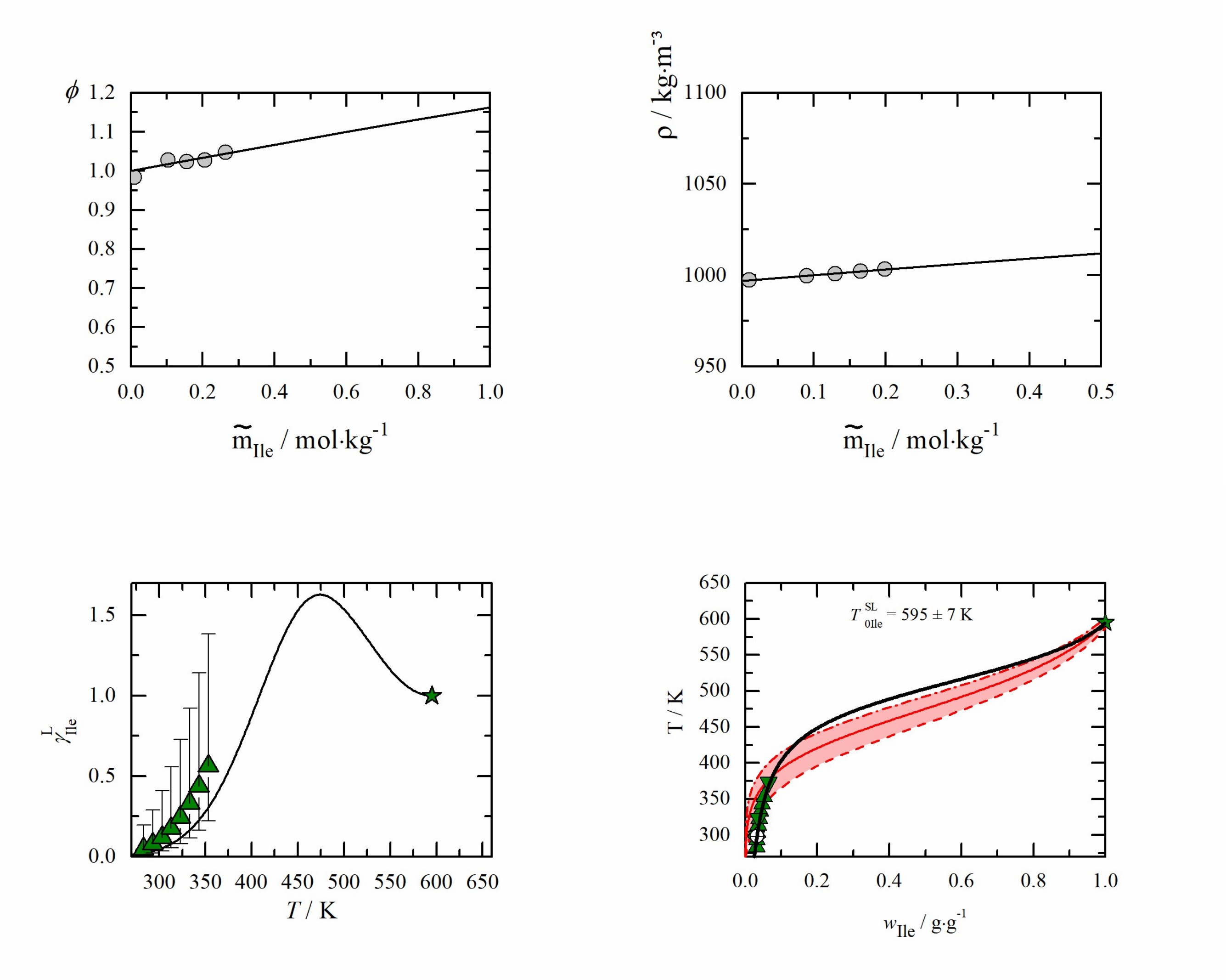

Figure S9 **Left top:** Densities of Ile + water solutions vs. molality at T = 298.15 K and p = 1 atm. Solid symbols represent experimental data Dalton, 1933[16] **Right top:** Osmotic coefficients of Ile + water solutions at T = 298.15 K and p = 1 atm. Solid symbols are experimental data from this work. The lines are the respective PC-SAFT modeling results with parameters from Table 2. **Left bottom:** Activity coefficients vs. temperature diagram. Uncertainties are based on the uncertainties of the melting properties. Solid symbols represent experimental data. ▲: Zumstein[17], **Right bottom:** Aqueous solubility as temperature vs. weight fraction diagram. The red area presents the solubility modeling assuming $\gamma_i^L = 1$ (eq. (1)) in the range of the uncertainties of the melting properties. Solid symbols represent experimental data. ▲ : Zumstein[17], ▼: Amend[11], The lines are the respective PC-SAFT modeling using the parameters from Table 2 and the melting properties from Table 3

Please do not adjust margins

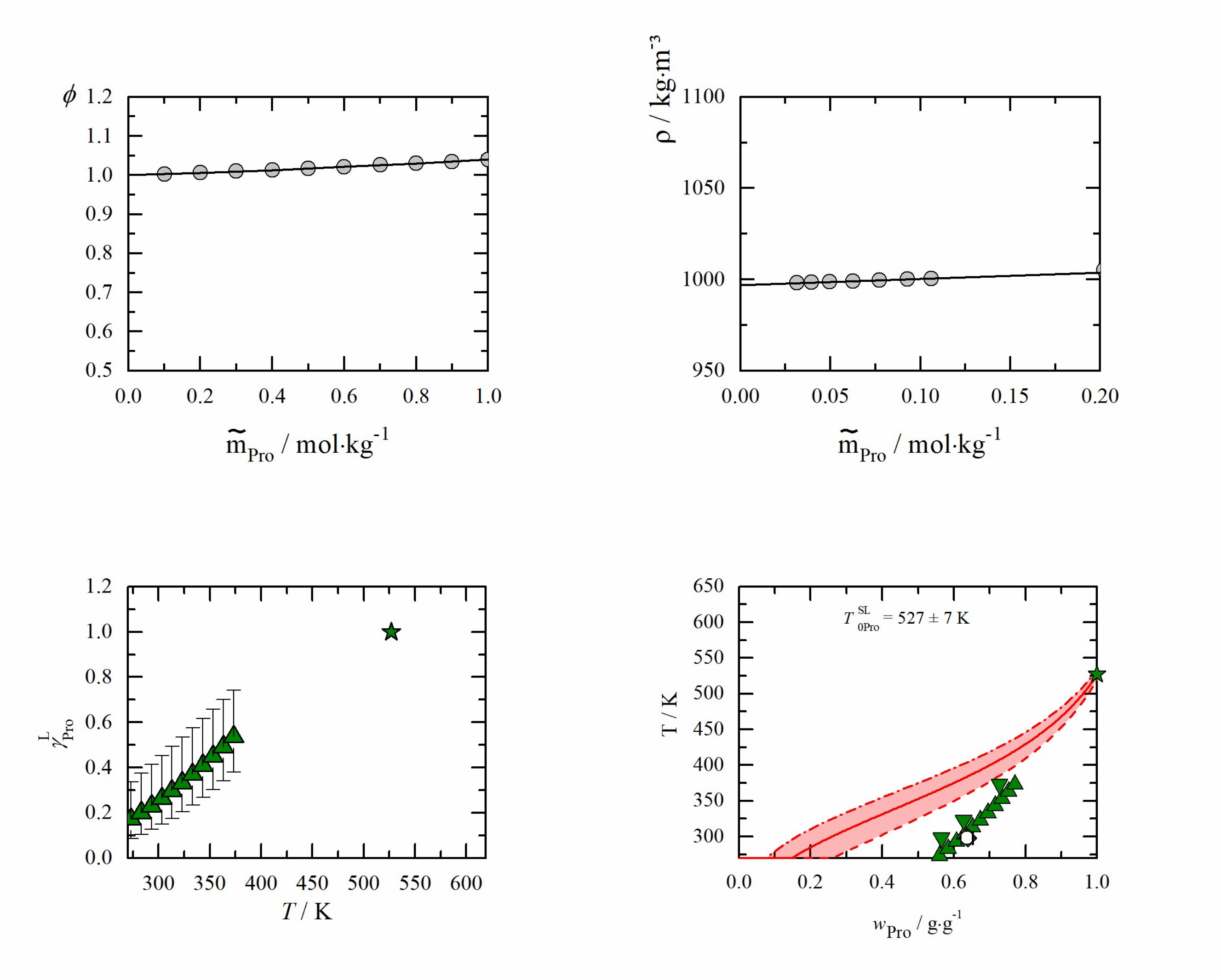

Figure S10 **Left top:** Densities of pro + water solutions vs. molality at T = 298.15 K and p = 1 atm. Solid symbols represent experimental data Ninni, 2001[18]. **Right top:** Osmotic coefficients pro + water solutions at T = 298.15 K and p = 1 atm. Solid symbols are experimental data Barrett, 1998[19]. The lines are the respective PC-SAFT modeling results with parameters from Table 2. **Left bottom:** Activity coefficients vs. temperature diagram. Uncertainties are based on the uncertainties of the melting properties. Solid symbols represent experimental data. ▲ : Lundbland[10] **Right bottom:** Aqueous solubility as temperature vs. weight fraction diagram. The red area presents the solubility modeling assuming $\gamma_i^L = 1$ (eq. (1)) in the range of the uncertainties of the melting properties. Solid symbols represent experimental data. ▲ : Lundbland[10], ▼: Amend[11], The lines are the respective PC-SAFT modeling using the parameters from Table 2 and the melting properties from Table 3

This journal is © The Royal Society of Chemistry 20xx

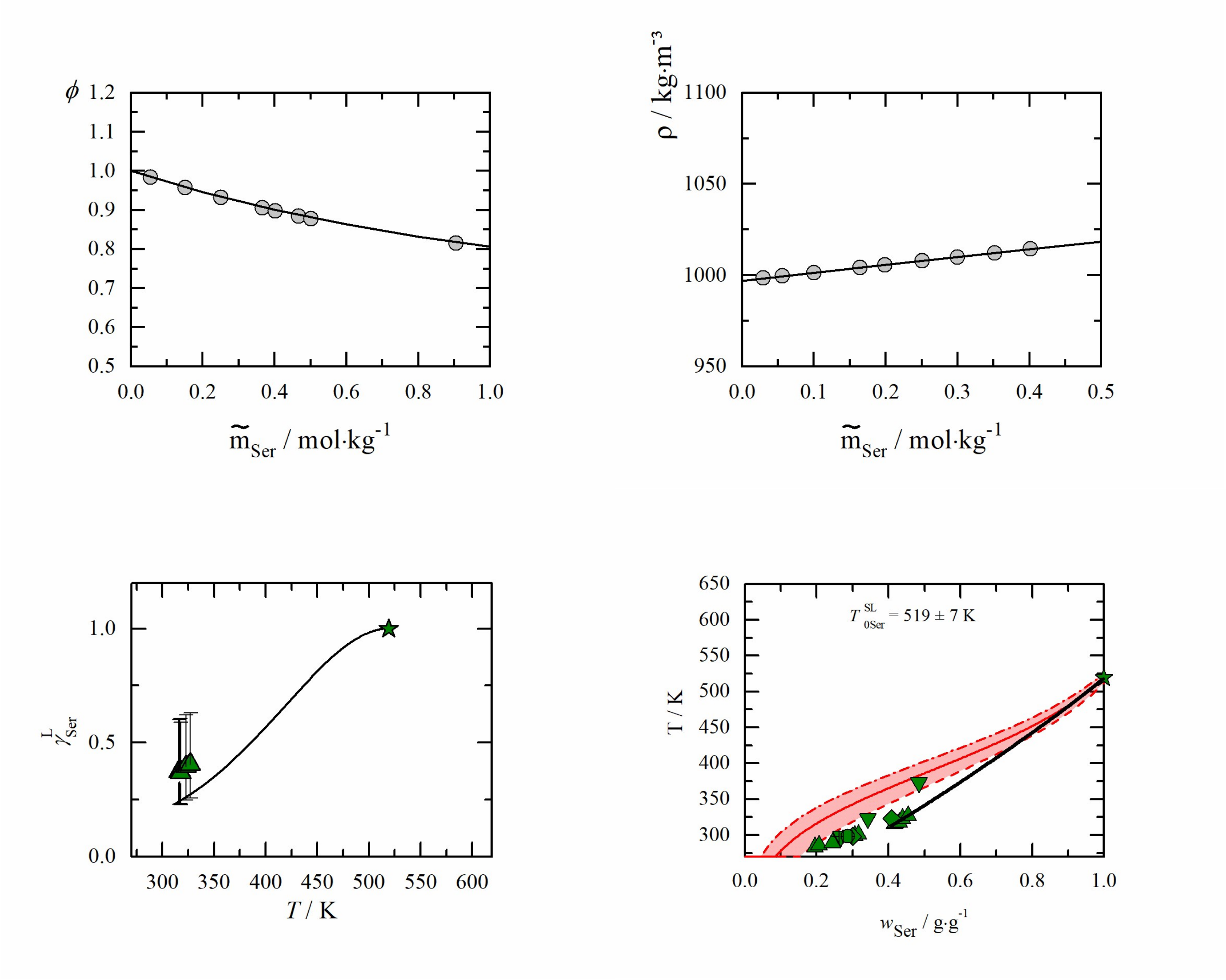

Figure S11 **Left top:** Densities of Ser + water solutions vs. molality at T = 298.15 K and p = 1 atm. Solid symbols represent experimental data Yan, 1999[9]. **Right top:** Osmotic coefficients of Ser + water solutions at T = 298.15 K and p = 1 atm. Solid symbols are experimental data Held, 2011[8]. The lines are the respective PC-SAFT modeling results with parameters from Table 2. **Left bottom:** Activity coefficients vs. temperature diagram. Uncertainties are based on the uncertainties of the melting properties. Solid symbols represent experimental data. ▲ Luk[20] **Right bottom:** Aqueous solubility as temperature vs. weight fraction diagram. The red area presents the solubility modeling assuming $\gamma_i^L = 1$ (eq. (1)) in the range of the uncertainties of the melting properties. Solid symbols represent experimental data. ▲ Luk[20], ▼: Amend[11]. The lines are the respective PC-SAFT modeling using the parameters from Table 2 and the melting properties from Table 3

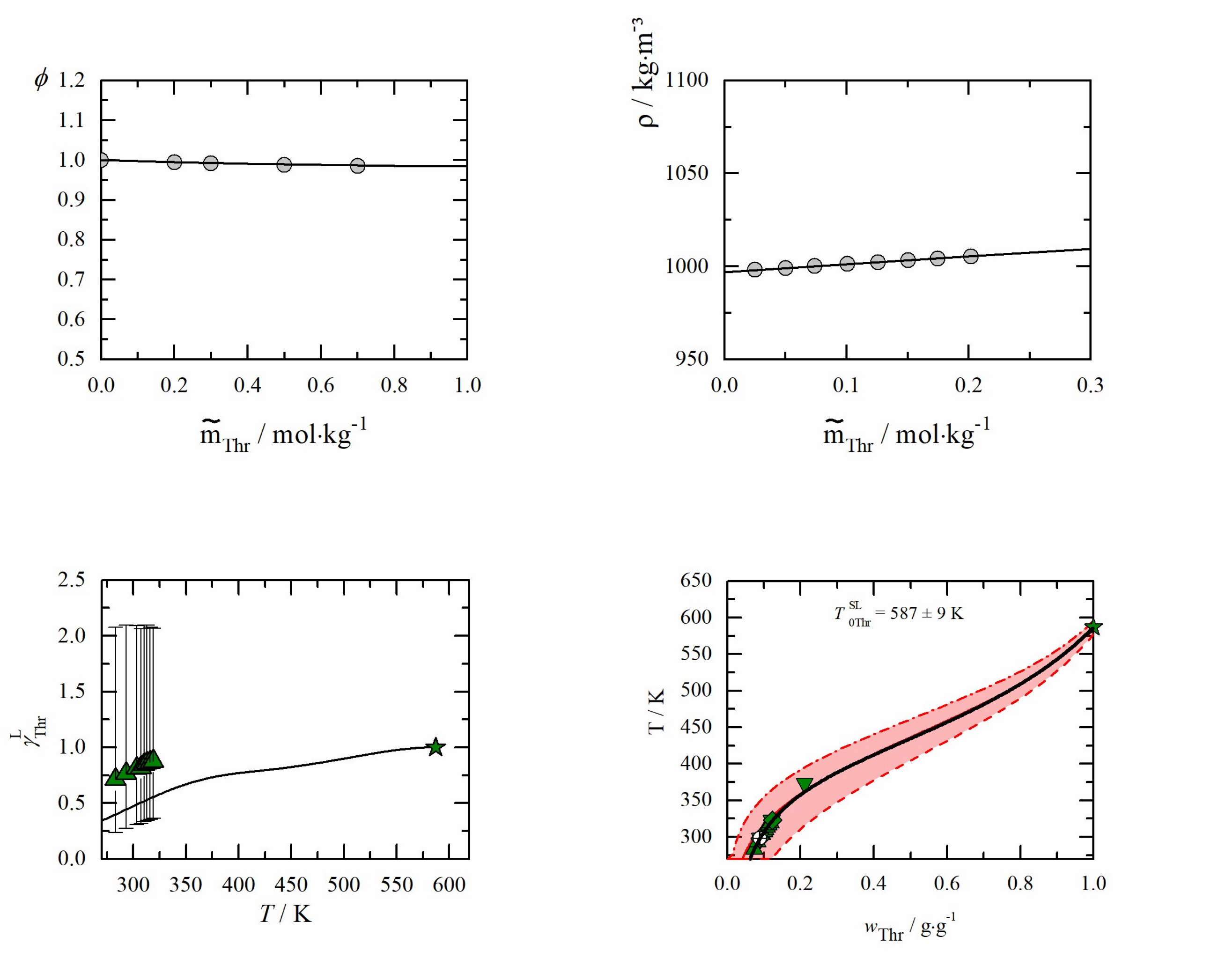

Figure S12 **Left top:** Densities of Thr + water solutions vs. molality at T = 298.15 K and p = 1 atm. Solid symbols represent experimental data Yan, 1999[9]. **Right top:** Osmotic coefficients of Thr + water solutions at T = 298.15 K and p = 1 atm. Solid symbols are experimental data Smith, 1940b[21]. The lines are the respective PC-SAFT modeling results with parameters from Table 2. **Left bottom:** Activity coefficients vs. temperature diagram. Uncertainties are based on the uncertainties of the melting properties. Solid symbols represent experimental data. ▲ : Lundbland[10] **Right bottom:** Aqueous solubility as temperature vs. weight fraction diagram. The red area presents the solubility modeling assuming $\gamma_i^L = 1$ (eq. (1)) in the range of the uncertainties of the melting properties. Solid symbols represent experimental data. ▲ : Lundbland[10], ▼: Amend[11], ◆: Ferreira[22]. The lines are the respective PC-SAFT modeling using the parameters from Table 2 and the melting properties from Table 3

 This journal is © The Royal Society of Chemistry 20xx

Please do not adjust margins

Please do not adjust margins

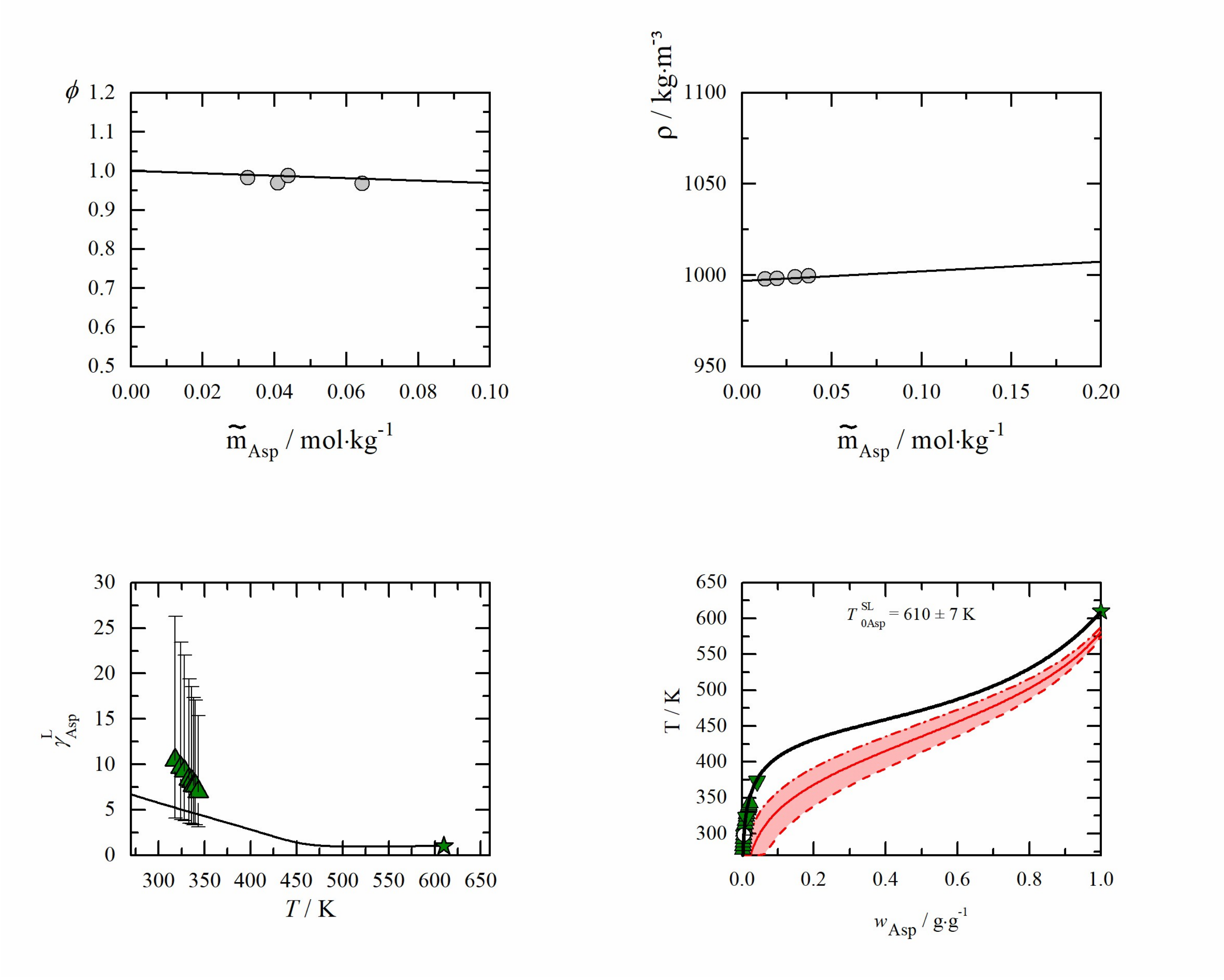

Figure S13 **Left top:** Densities of Asp + water solutions vs. molality at T = 298.15 K and p = 1 atm. Solid symbols represent experimental data from this work. **Right top:** Osmotic coefficients of Asp + water solutions at T = 298.15 K and p = 1 atm. Solid symbols are experimental data from this work. The lines are the respective PC-SAFT modeling results with parameters from Table 2. **Left bottom:** Activity coefficients vs. temperature diagram. Uncertainties are based on the uncertainties of the melting properties. Solid symbols represent experimental data. ▲ Apelblat[23], **Right bottom:** Aqueous solubility as temperature vs. weight fraction diagram. The red area presents the solubility modeling assuming $\gamma_i^L = 1$ (eq. (1)) in the range of the uncertainties of the melting properties. Solid symbols represent experimental data. ▲ Apelblat[23], ▼: Amend[11], The lines are the respective PC-SAFT modeling using the parameters from Table 2 and the melting properties from Table 3

Please do not adjust margins

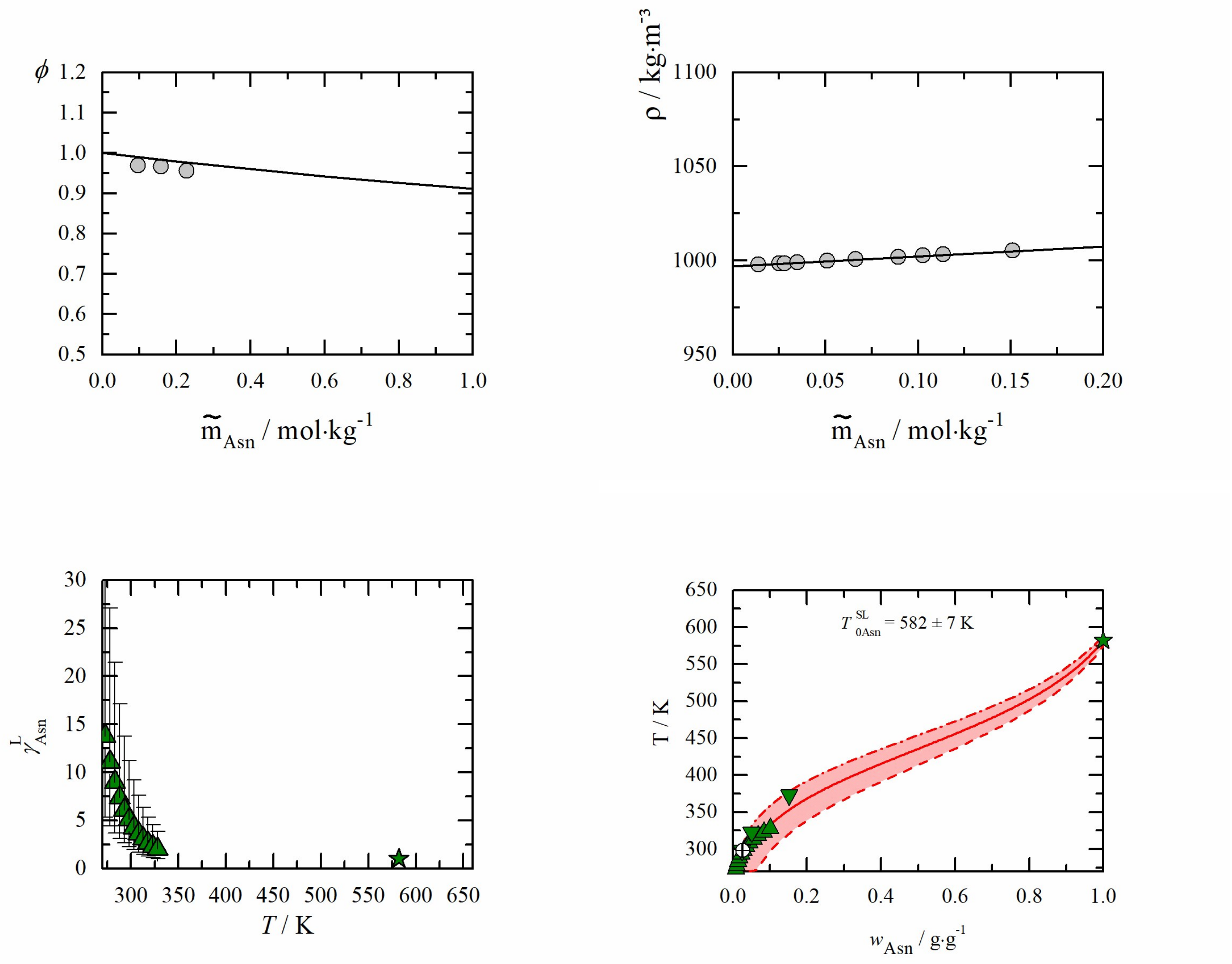

Figure S14 **Left top:** Densities of Asn + water solutions vs. molality at T = 298.15 K and p = 1 atm. Solid symbols represent experimental data Hakin, 1995[24]. **Right top:** Osmotic coefficients of Asn + water solutions at T = 298.15 K and p = 1 atm. Solid symbols are experimental data from this work. The lines are the respective PC-SAFT modeling results with parameters from Table 2. **Left bottom:** Activity coefficients vs. temperature diagram. Uncertainties are based on the uncertainties of the melting properties. Solid symbols represent experimental data. ▲ Dalton[16], **Right bottom:** Aqueous solubility as temperature vs. weight fraction diagram. The red area presents the solubility modeling assuming $\gamma_i^L = 1$ (eq. (1)) in the range of the uncertainties of the melting properties. Solid symbols represent experimental data. ▲ Dalton[16], ▼: Amend[11], The lines are the respective PC-SAFT modeling using the parameters from Table 2 and the melting properties from Table 3

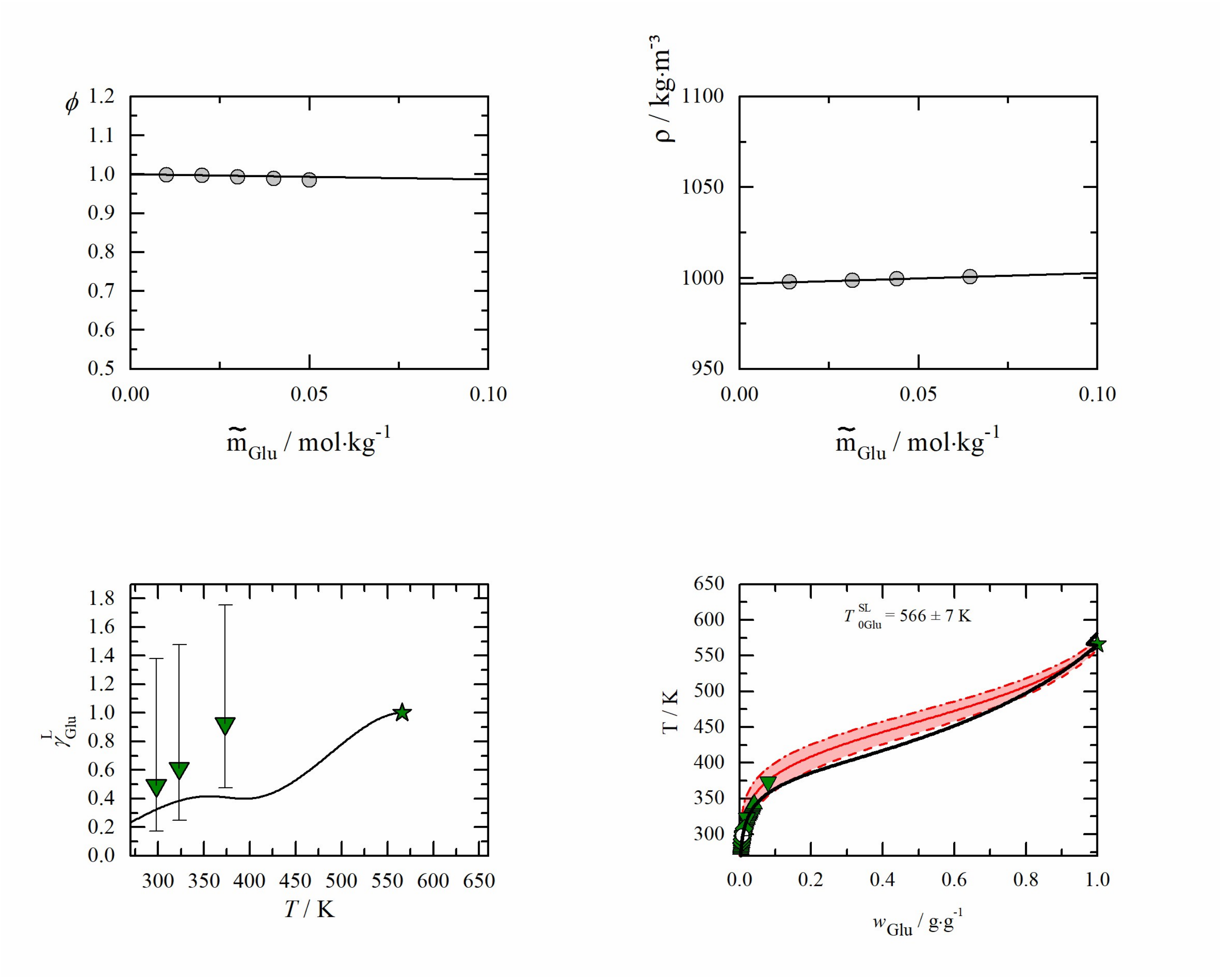

Figure S15 **Left top:** Densities of Glu + water solutions vs. molality at T = 298.15 K and p = 1 atm. Solid symbols represent experimental data from this work. **Right top:** Osmotic coefficients of Glu + water solutions at T = 298.15 K and p = 1 atm. Solid symbols are experimental data from this work. The lines are the respective PC-SAFT modeling results with parameters from Table 2. **Left bottom:** Activity coefficients vs. temperature diagram. Uncertainties are based on the uncertainties of the melting properties. Solid symbols represent experimental data**.** ▲ Matsuo[25], **Right bottom:** Aqueous solubility as temperature vs. weight fraction diagram. The red area presents the solubility modeling assuming $\gamma_i^L = 1$ (eq. (1)) in the range of the uncertainties of the melting properties. Solid symbols represent experimental data**.** ▲ Matsuo[25], ▼: Amend[11], ◆: Yalkowsky[26]. The lines are the respective PC-SAFT modeling using the parameters from Table 2 and the melting properties from Table 3

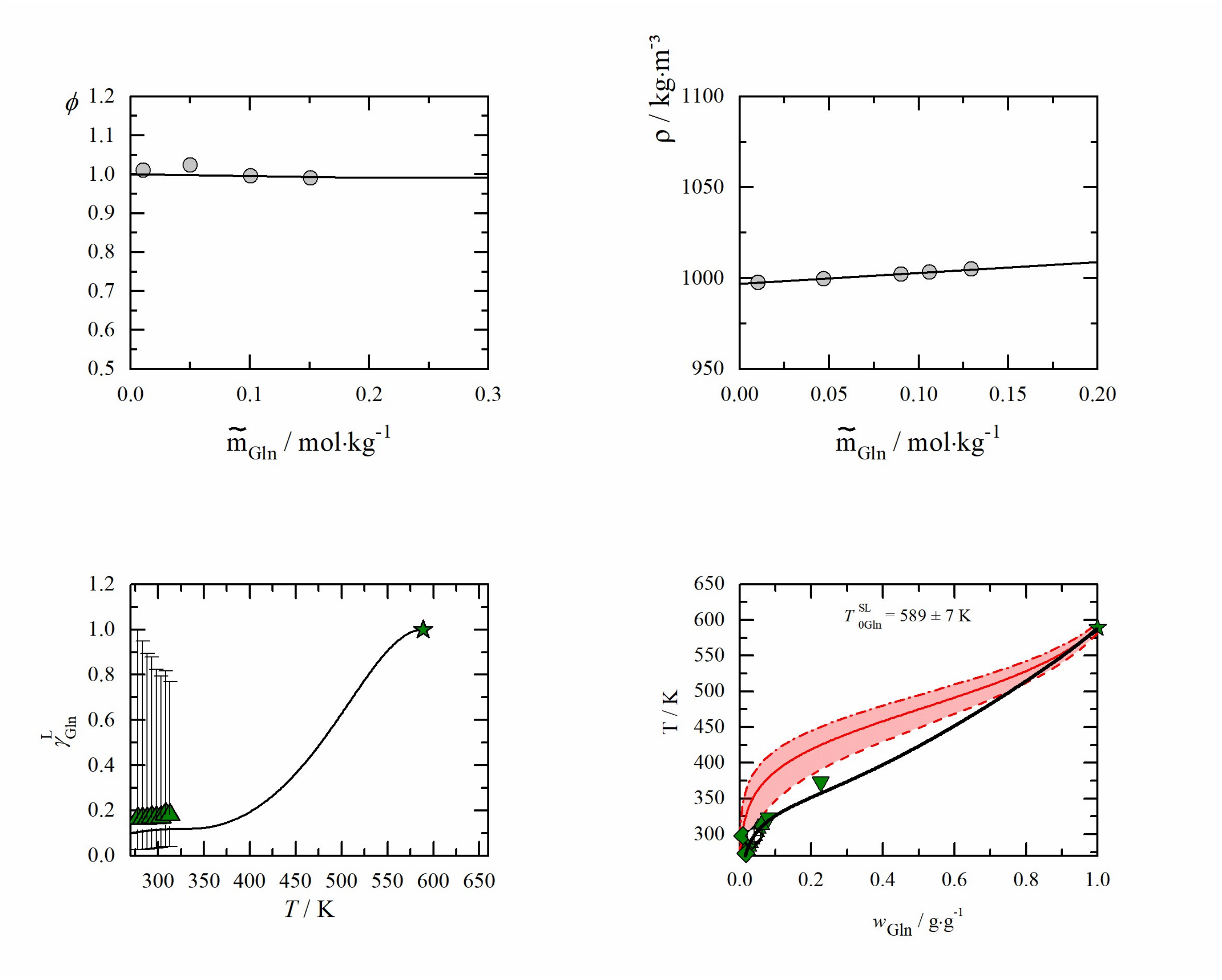

Figure S16 **Left top:** Densities of Gln + water solutions vs. molality at T = 298.15 K and p = 1 atm. Solid symbols represent experimental data from this work. **Right top:** Osmotic coefficients of Gln + water solutions at T = 298.15 K and p = 1 atm. Solid symbols are experimental data from this work. The lines are the respective PC-SAFT modeling results with parameters from Table 2. **Left bottom:** Activity coefficients vs. temperature diagram. Uncertainties are based on the uncertainties of the melting properties. Solid symbols represent experimental data. ▲ Yu[27], **Right bottom:** Aqueous solubility as temperature vs. weight fraction diagram. The red area presents the solubility modeling assuming $\gamma_i^L = 1$ (eq. (1)) in the range of the uncertainties of the melting properties. Solid symbols represent experimental data. ▲ Yu[27], ▼: Amend[11], ◆: Yalkowsky[26]. The lines are the respective PC-SAFT modeling using the parameters from Table 2 and the melting properties from Table 3

This journal is © The Royal Society of Chemistry 20xx
Please do not adjust margins

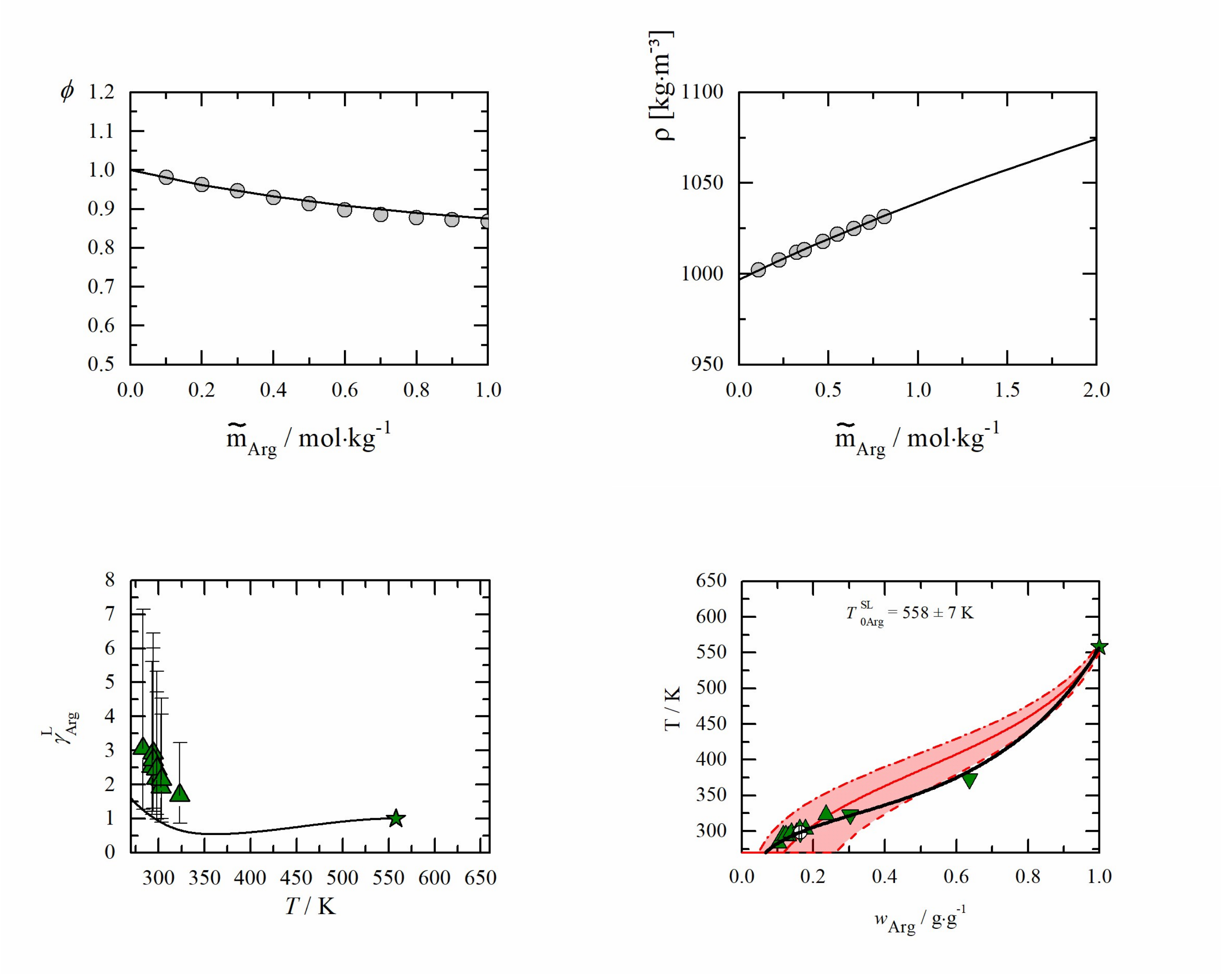

Figure S17 **Left top:** Densities of Arg + water solutions vs. molality at T = 298.15 K and p = 1 atm. Solid symbols represent experimental data Ninni, 2001,[18] **Right top:** Osmotic coefficients of Arg + water solutions at T = 298.15 K and p = 1 atm. Solid symbols are experimental data Bonner, 1982[28].The lines are the respective PC-SAFT modeling results with parameters from Table 2. **Left bottom:** Activity coefficients vs. temperature diagram. Uncertainties are based on the uncertainties of the melting properties. Solid symbols represent experimental data**.** ▲Yalkowsky[26], **Right bottom:** Aqueous solubility as temperature vs. weight fraction diagram. The red area presents the solubility modeling assuming $\gamma_i^L = 1$ (eq. (1)) in the range of the uncertainties of the melting properties. Solid symbols represent experimental data**.** ▲Yalkowsky[26], ▼: Amend[11], The lines are the respective PC-SAFT modeling using the parameters from Table 2 and the melting properties from Table 3

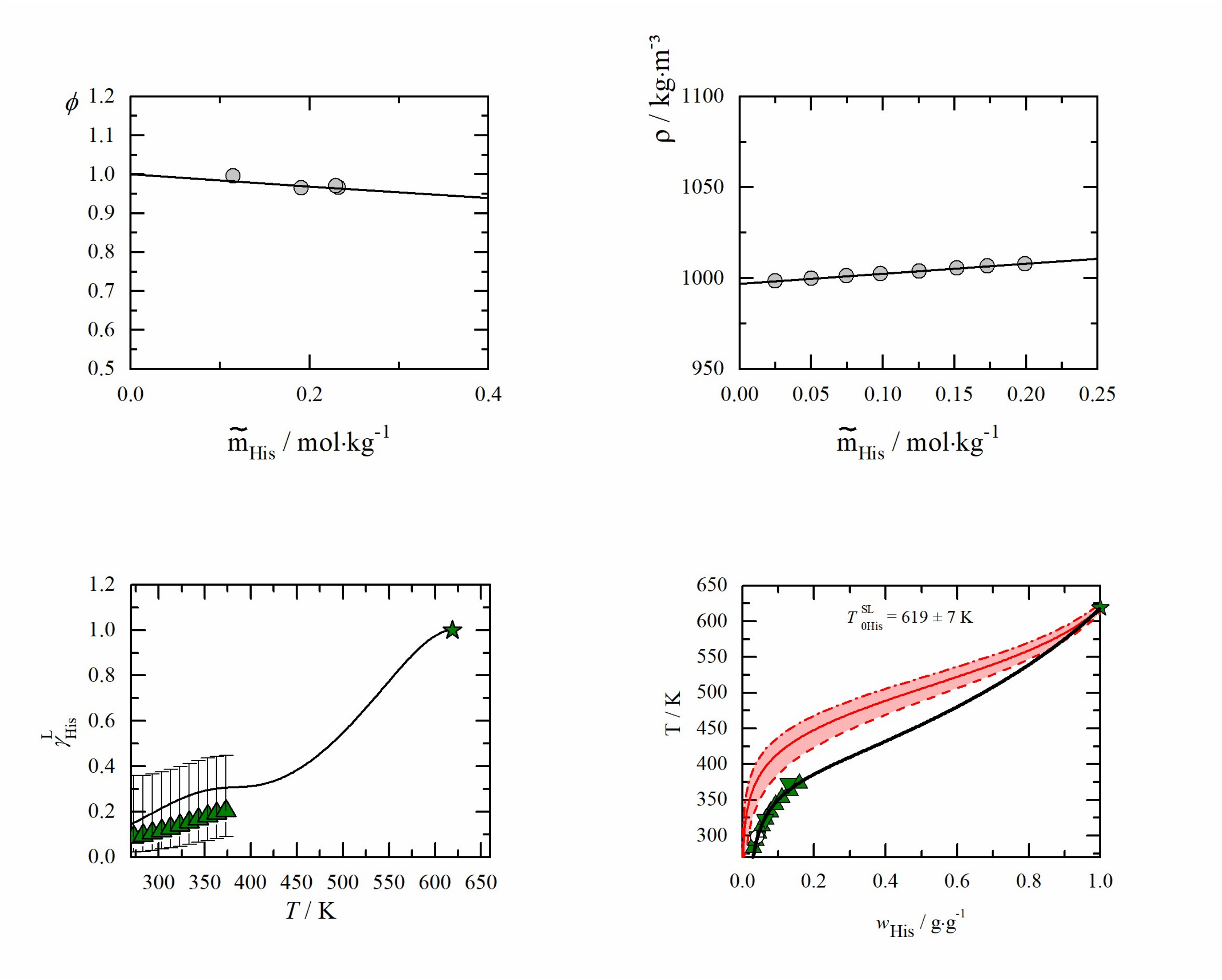

Figure S18 **Left top:** Densities of His + water solutions vs. molality at T = 298.15 K and p = 1 atm. Solid symbols represent experimental data Yan, 1999[9] **Right top:** Osmotic coefficients of His + water solutions at T = 298.15 K and p = 1 atm. Solid symbols are experimental data Tsurko, 2007[29]. The lines are the respective PC-SAFT modeling results with parameters from Table 2. **Left bottom:** Activity coefficients vs. temperature diagram. Uncertainties are based on the uncertainties of the melting properties. Solid symbols represent experimental data. ▲ Kustov[30], **Right bottom:** Aqueous solubility as temperature vs. weight fraction diagram. The red area presents the solubility modeling assuming $\gamma_i^L = 1$ (eq. (1)) in the range of the uncertainties of the melting properties. Solid symbols represent experimental data. ▲ Kustov[30], ▼: Amend[11], The lines are the respective PC-SAFT modeling using the parameters from Table 2 and the melting properties from Table 3

This journal is © The Royal Society of Chemistry 20xx

Please do not adjust margins

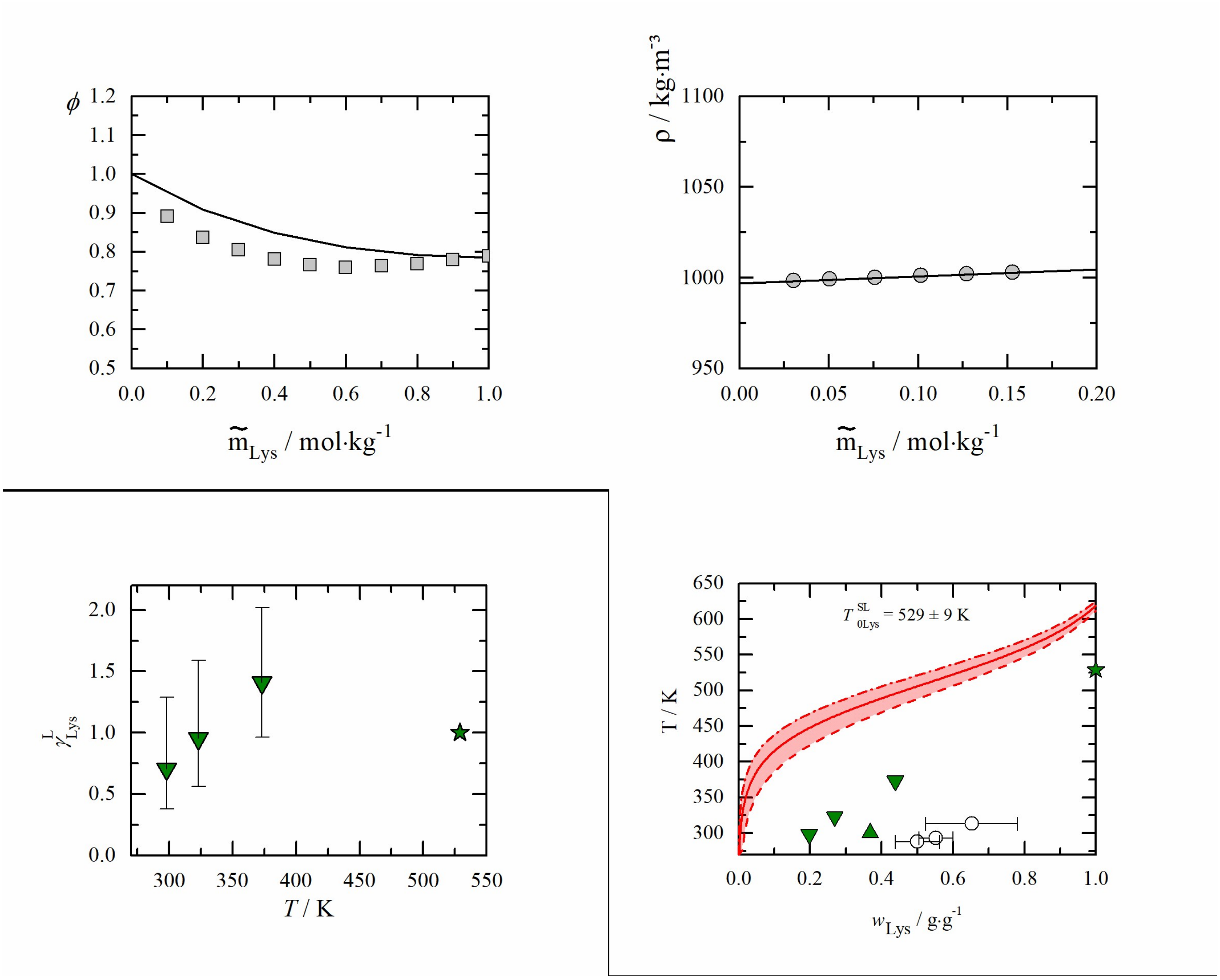

Figure S19 **Left top:** Densities of Lys + water solutions vs. molality at T = 298.15 K and p = 1 atm. Solid symbols represent experimental data Jolicoeur, 1986[31] **Right top:** Osmotic coefficients of Lys + water solutions at T = 298.15 K and p = 1 atm. Solid symbols are experimental data Bonner, 1982[28]. The lines are the respective PC-SAFT modeling results with parameters from Table 2. **Left bottom:** Activity coefficients vs. temperature diagram. Uncertainties are based on the uncertainties of the melting properties. Solid symbols represent experimental data**.** ▼ Amend[11] **Right bottom:** Aqueous solubility as temperature vs. weight fraction diagram. The red area presents the solubility modeling assuming $\gamma_i^L = 1$ (eq. (1)) in the range of the uncertainties of the melting properties. Solid symbols represent experimental data**.** ▲ Yalkowsky[26] ▼: Amend[11], The lines are the respective PC-SAFT modeling using the parameters from Table 2 and the melting properties from Table 3

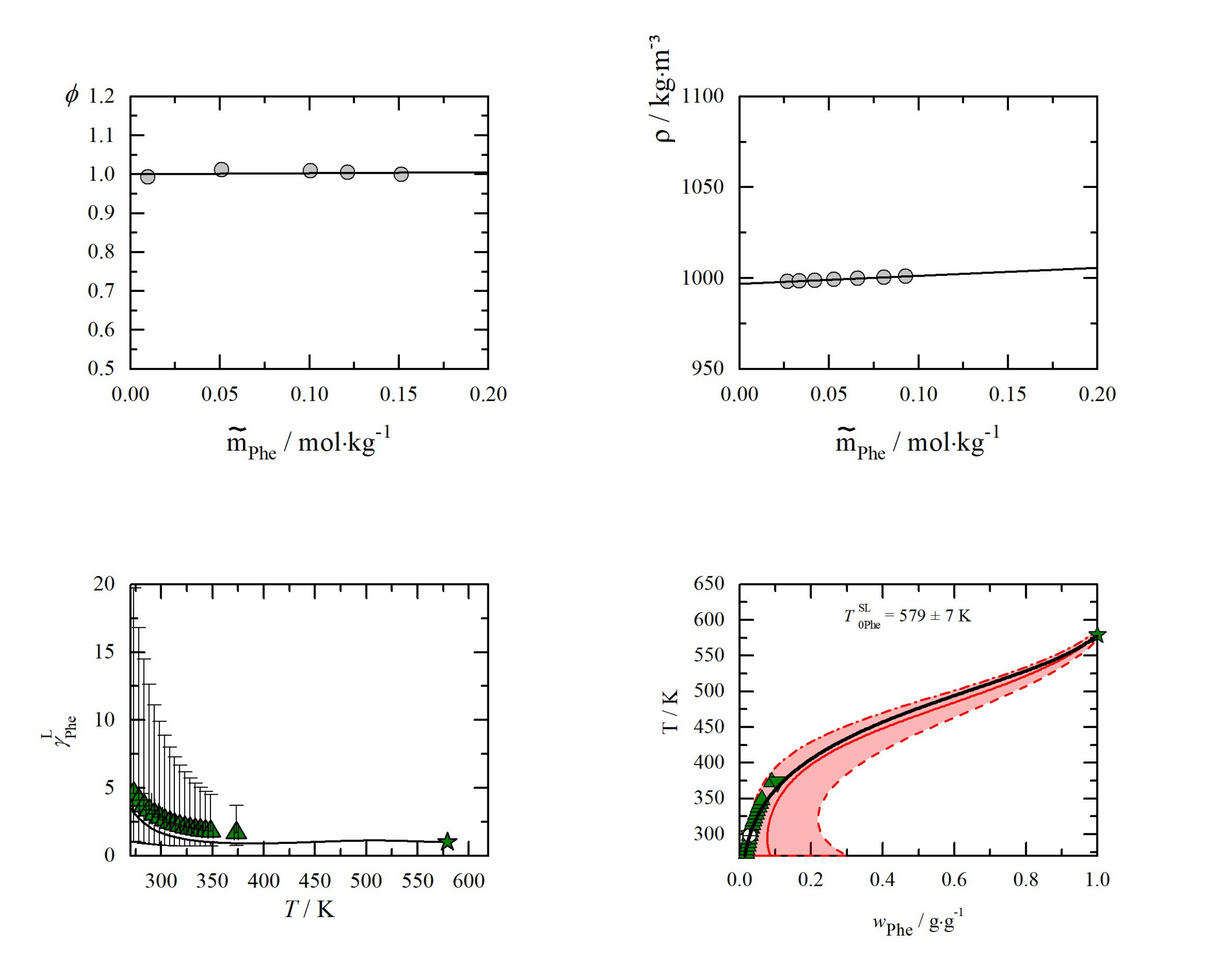

Figure S20 **Left top:** Densities of Phe + water solutions vs. molality at T = 298.15 K and p = 1 atm. Solid symbols represent experimental data Yan, 1999[9]. **Right top:** Osmotic coefficients of Phe + water solutions at T = 298.15 K and p = 1 atm. Solid symbols are experimental data from this work. The lines are the respective PC-SAFT modeling results with parameters from Table 2. **Left bottom:** Activity coefficients vs. temperature diagram. Uncertainties are based on the uncertainties of the melting properties. Solid symbols represent experimental data. ▲ Dalton[16], **Right bottom:** Aqueous solubility as temperature vs. weight fraction diagram. The red area presents the solubility modeling assuming $\gamma_i^L = 1$ (eq. (1)) in the range of the uncertainties of the melting properties. Solid symbols represent experimental data. ▲Dalton[16], ▼: Amend[11], The lines are the respective PC-SAFT modeling using the parameters from Table 2 and the melting properties from Table 3

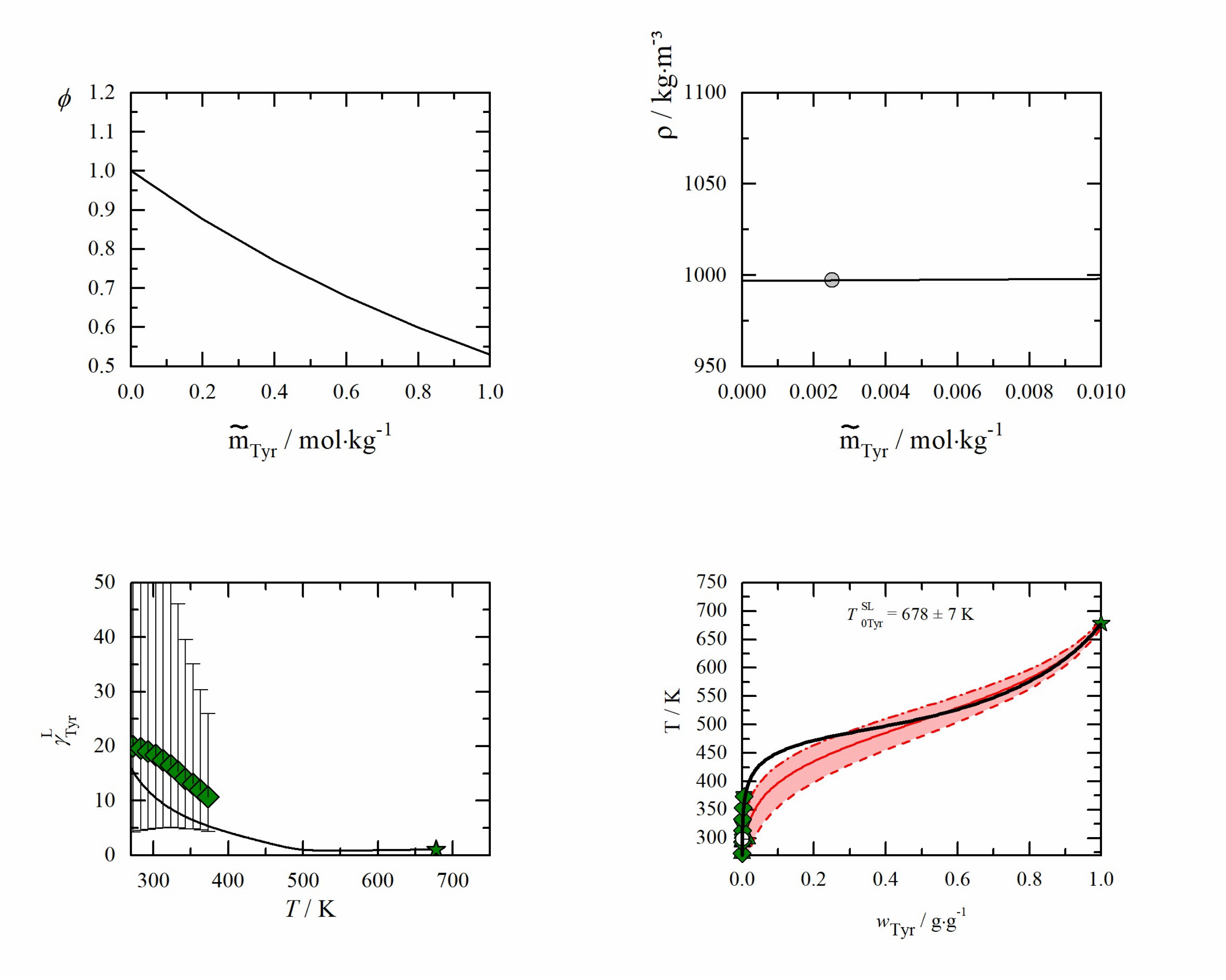

Figure S21 **Left top:** Densities of Tyr + water solutions vs. molality at T = 298.15 K and p = 1 atm. Solid symbols represent experimental data Carter, 1996[32] **Right top:** Osmotic coefficients of Tyr + water solutions at T = 298.15 K and p = 1 atm. The lines are the respective PC-SAFT modeling results with parameters from Table 2. **Left bottom:** Activity coefficients vs. temperature diagram. Uncertainties are based on the uncertainties of the melting properties. Solid symbols represent experimental data. ◆ : Lundbland[10] **Right bottom:** Aqueous solubility as temperature vs. weight fraction diagram. The red area presents the solubility modeling assuming $\gamma_i^L = 1$ (eq. (1)) in the range of the uncertainties of the melting properties. Solid symbols represent experimental data**.** ▲Yalkowsky[26], ▼: Amend[11], ◆ : Lundbland[10]. The lines are the respective PC-SAFT modeling using the parameters from Table 2 and the melting properties from Table 3

Please do not adjust margins

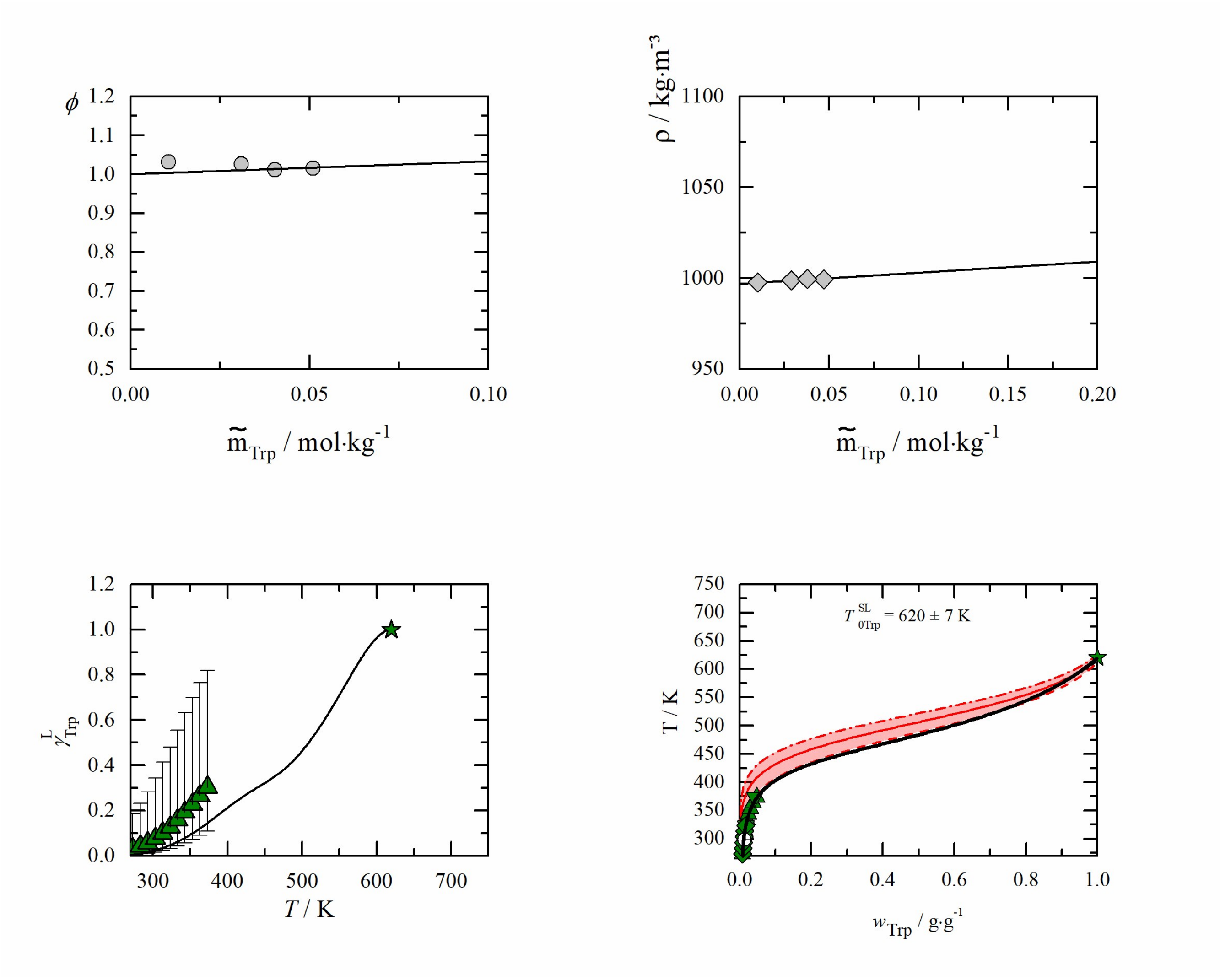

Figure S22 **Left top:** Densities of Trp + water solutions vs. molality at T = 298.15 K and p = 1 atm. Solid symbols represent experimental data from this work. **Right top:** Osmotic coefficients of Trp + water solutions at T = 298.15 K and p = 1 atm. Solid symbols are experimental data from this work. The lines are the respective PC-SAFT modeling results with parameters from Table 2. **Left bottom:** Activity coefficients vs. temperature diagram. Uncertainties are based on the uncertainties of the melting properties. Solid symbols represent experimental data**.** ▲ : Lundbland[10] **Right bottom:** Aqueous solubility as temperature vs. weight fraction diagram. The red area presents the solubility modeling assuming $\gamma_i^L = 1$ (eq. (1)) in the range of the uncertainties of the melting properties. Solid symbols represent experimental data**.** ▲ : Lundbland[10], ▼: Amend[11], ◆: Dalton[16]The lines are the respective PC-SAFT modeling using the parameters from Table 2 and the melting properties from Table 3

This journal is © The Royal Society of Chemistry 20xx

Please do not adjust margins

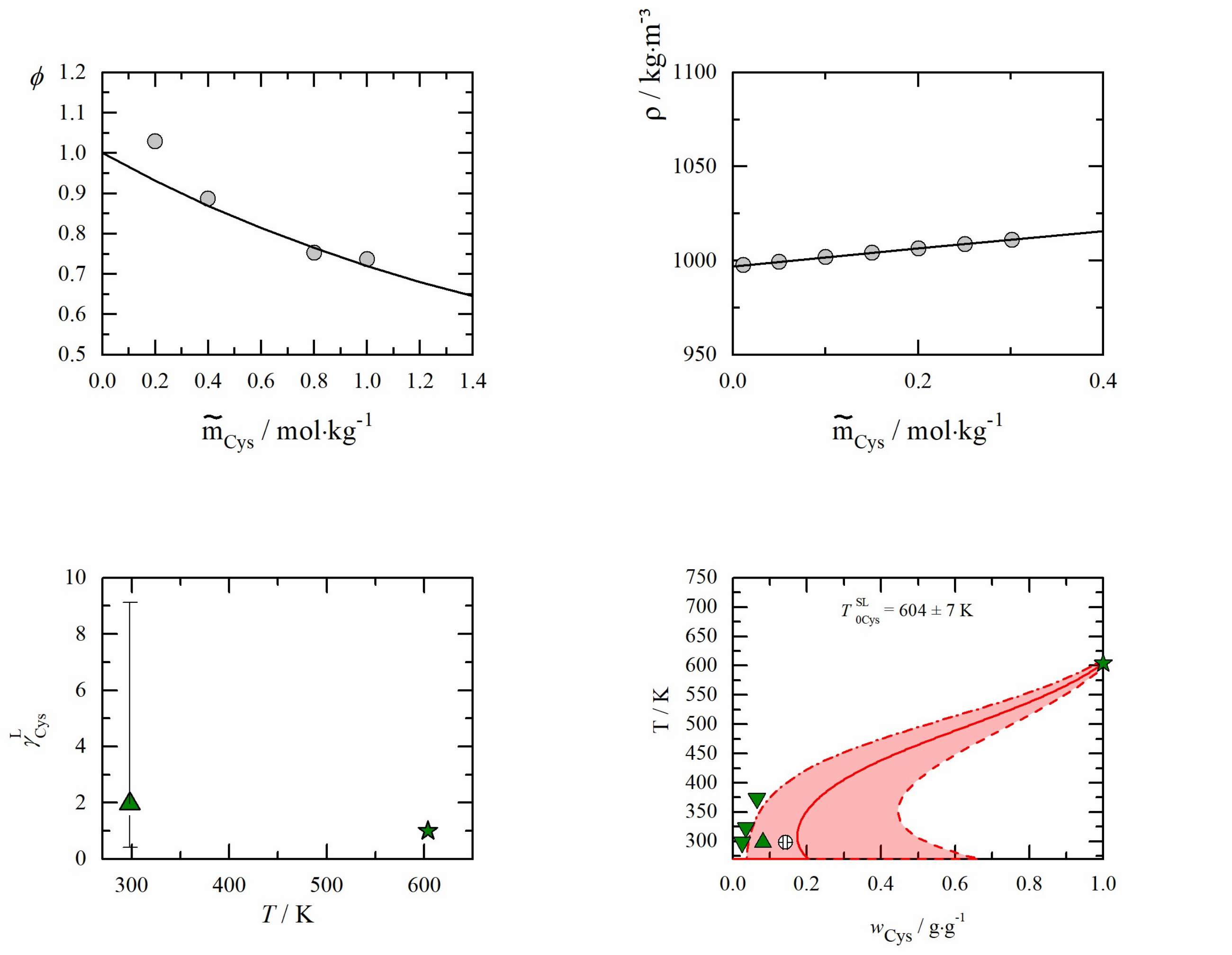

Figure S23 **Left top:** Densities of Cys + water solutions vs. molality at T = 298.15 K and p = 1 atm. Solid symbols represent experimental data Kharakoz, 1989[33]. **Right top:** Osmotic coefficients of Cys + water solutions at T = 298.15 K and p = 1 atm. Solid symbols are experimental data from this work. The lines are the respective PC-SAFT modeling results with parameters from Held, 2011[8] **Left bottom:** Activity coefficients vs. temperature diagram. Uncertainties are based on the uncertainties of the melting properties. Solid symbols represent experimental data. ▲ Bowden, 2018[34], **Right bottom:** Aqueous solubility as temperature vs. weight fraction diagram. The red area presents the solubility modeling assuming $\gamma_i^L = 1$ (eq. (1)) in the range of the uncertainties of the melting properties. Solid symbols represent experimental data. ▲ Bowden, 2018[34],, ▼: Amend[11], The lines are the respective PC-SAFT modeling using the parameters from Table 2 and the melting properties from Table 3

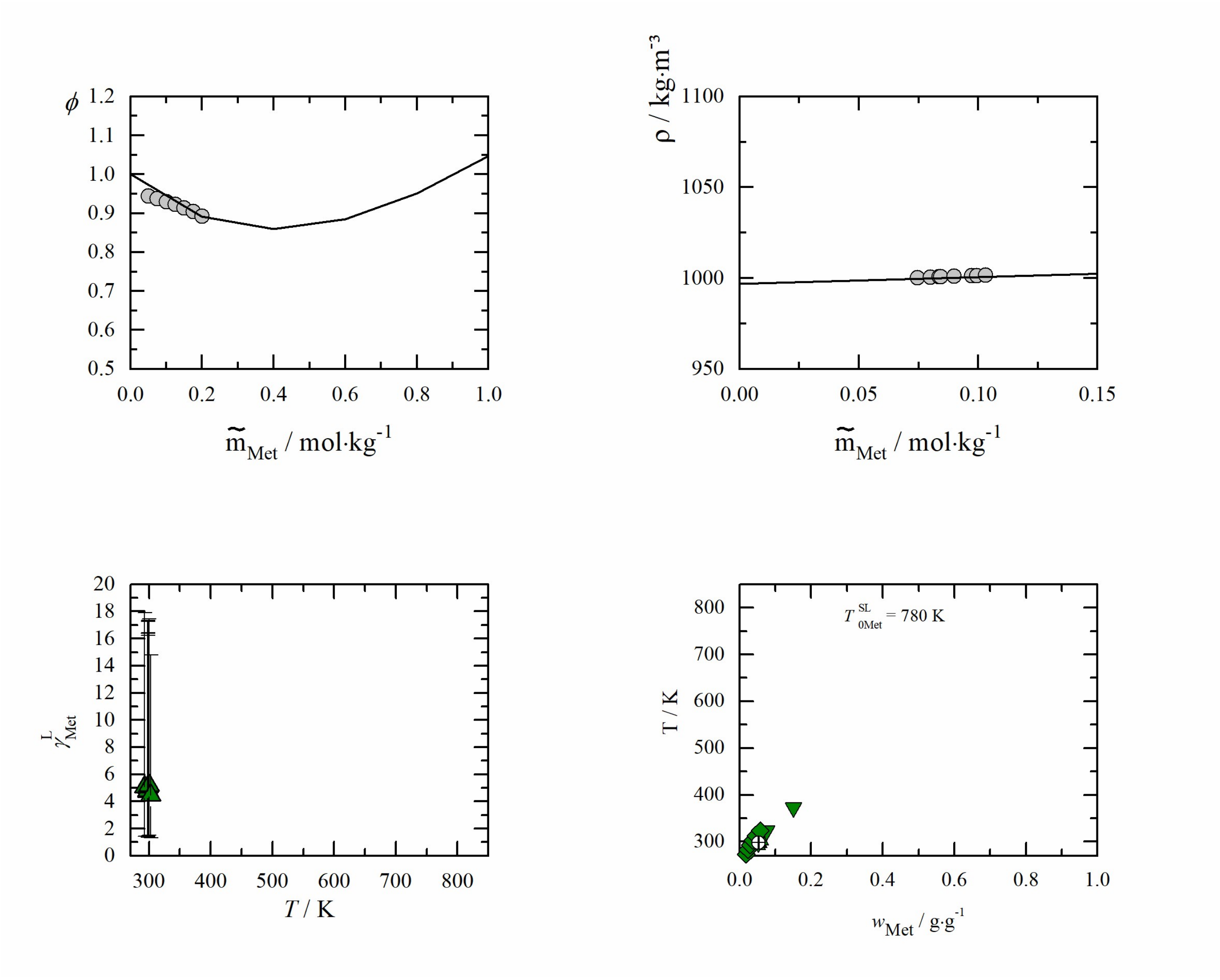

Figure S24 **Left top:** Densities of Met + water solutions vs. molality at T = 298.15 K and p = 1 atm. Solid symbols represent experimental data Kikuchi, 1995[14], **Right top:** Osmotic coefficients of Met + water solutions at T = 298.15 K and p = 1 atm. Solid symbols are experimental data from this work. The lines are the respective PC-SAFT modeling results with parameters from Held, 2011[8] **Left bottom:** Activity coefficients vs. temperature diagram. Uncertainties are based on the uncertainties of the melting properties. Solid symbols represent experimental data**.** ▲ Yalkowsky[26] **Right bottom:** Aqueous solubility as temperature vs. weight fraction diagram. The red area presents the solubility modeling assuming $\gamma_i^L = 1$ (eq. (1)) in the range of the uncertainties of the melting properties. Solid symbols represent experimental data**.** ▲: Yalkowsky[26] ▼: Amend[11], ◆: Fuchs[35] The lines are the respective PC-SAFT modeling using the parameters from Table 2 and the melting properties from Table 3

 This journal is © The Royal Society of Chemistry 20xx

Please do not adjust margins

PXRD measurements of pure amino acids and the amino acids in solid phase equilibrated with saturated solution

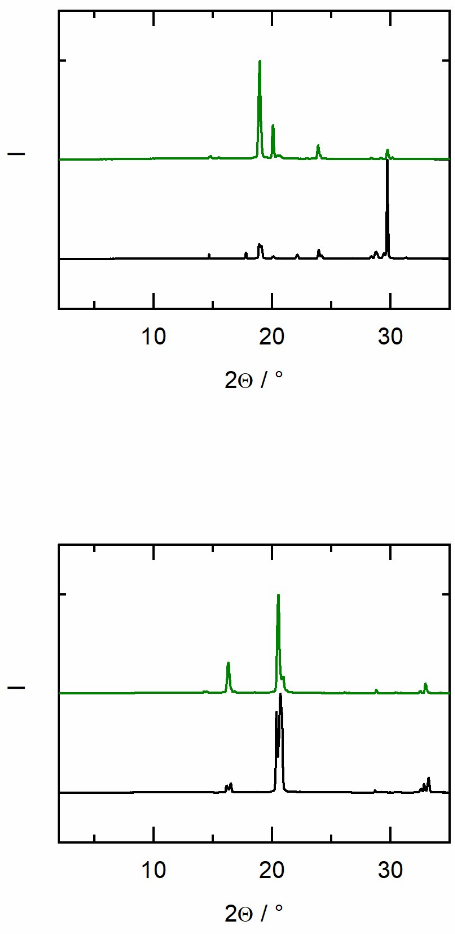

Figure S25 PXRD diffractograms of the amino acids Gly (top) and Ala (bottom). Green line: pure component; black line: solid phase of the supersaturated solution at T = 298.15 K and p = 1 atm.

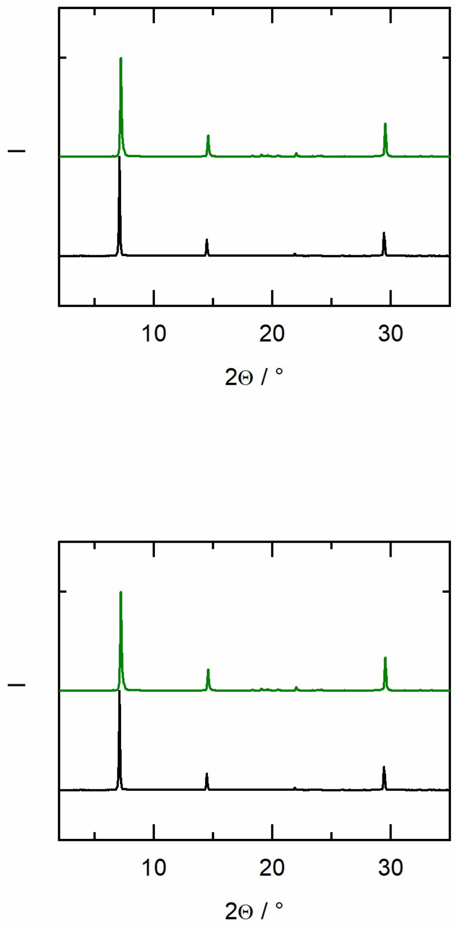

Figure S26 PXRD diffractograms of the amino acids Val (Top) and Leu (bottom). Green line: pure component; black line: solid phase of the supersaturated solution at T = 298.15 K and p = 1 atm.

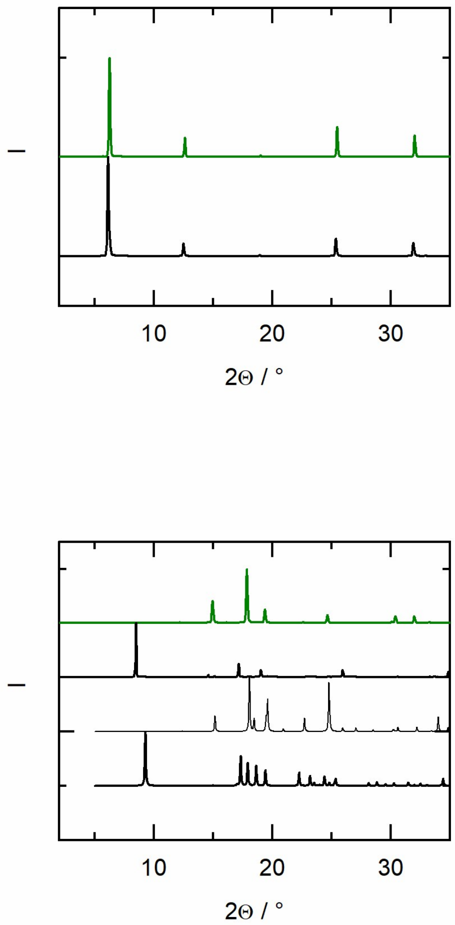

Figure S27 PXRD diffractograms of the amino acids Ile (Top) and Pro (bottom). Green line: pure component; black line: solid phase of the supersaturated solution at T = 298.15 K and p = 1 atm. Additionally for Pro two diffractograms from the Cambridge Crystallographic data center (CCDC) is shown.

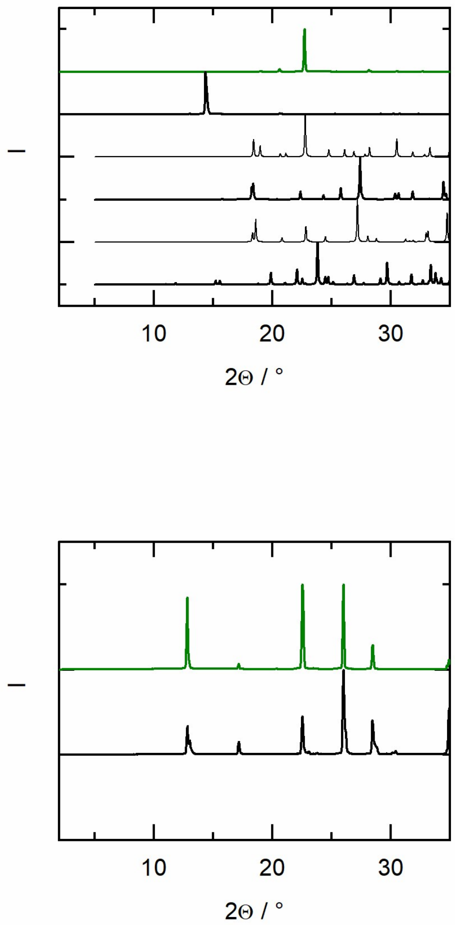

Figure S28 PXRD diffractograms of the amino acids Ser (Top) and Thr (bottom). Green line: pure component; black line: solid phase of the supersaturated solution at T = 298.15 K and p = 1 atm. Additionally for Ser four diffractograms from the Cambridge Crystallographic data center (CCDC) is shown.

This journal is © The Royal Society of Chemistry 20xx

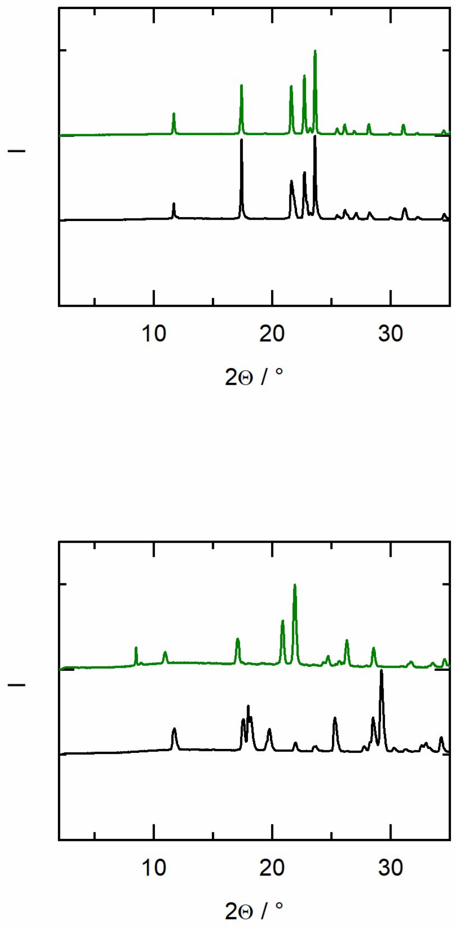

Figure S29 PXRD diffractograms of the amino acids Asp (Top) and Asn (bottom). Green line: pure component; black line: solid phase of the supersaturated solution at T = 298.15 K and p = 1 atm.

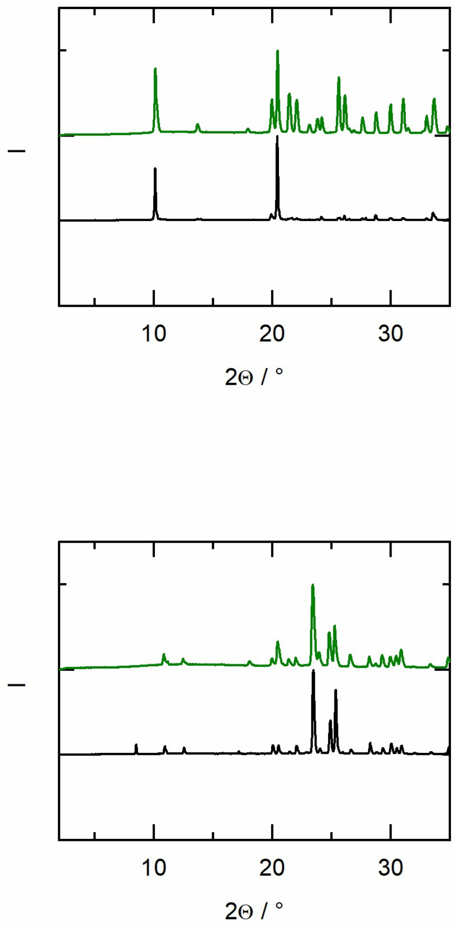

Figure S30 PXRD diffractograms of the amino acids Glu (Top) and Gln (bottom). Green line: pure component; black line: solid phase of the supersaturated solution at T = 298.15 K and p = 1 atm.

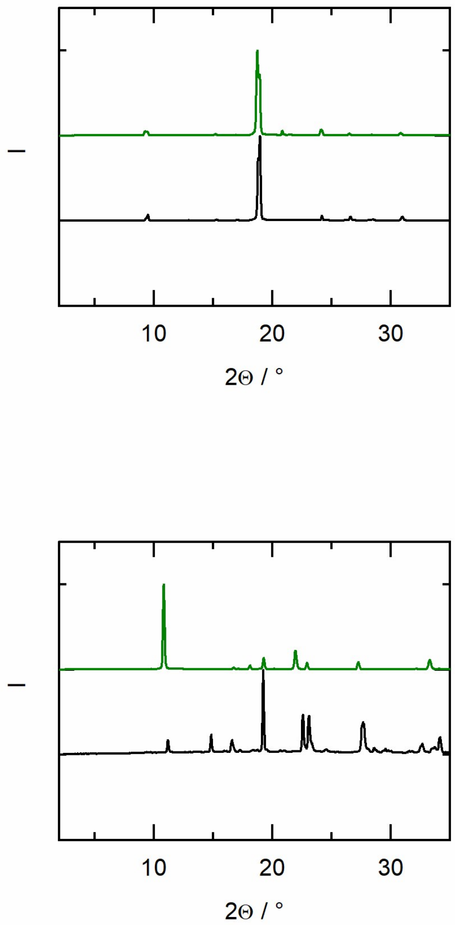

Figure S31 PXRD diffractograms of the amino acids His (Top) and Arg (bottom). Green line: pure component; black line: solid phase of the supersaturated solution at T = 298.15 K and p = 1 atm.

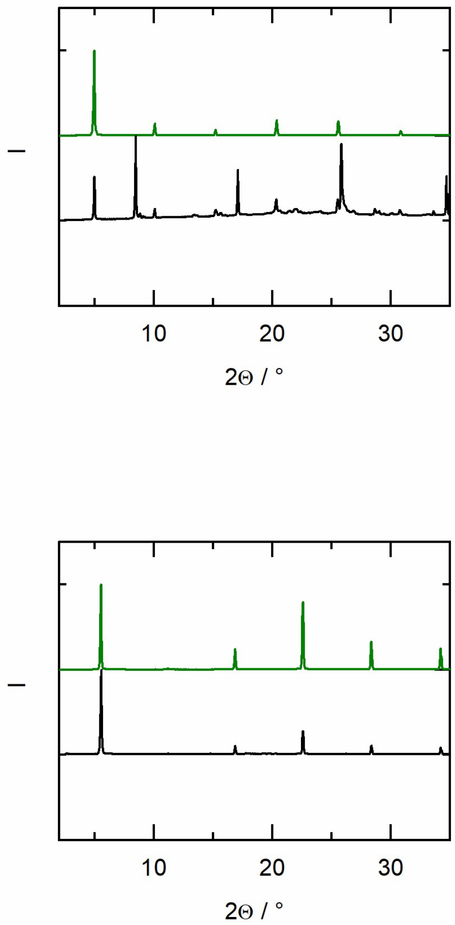

Figure S32 PXRD diffractograms of the amino acids Lys (Top) and Phe (bottom). Green line: pure component; black line: solid phase of the supersaturated solution at T = 298.15 K and p = 1 atm.

This journal is © The Royal Society of Chemistry 20xx
Please do not adjust margins

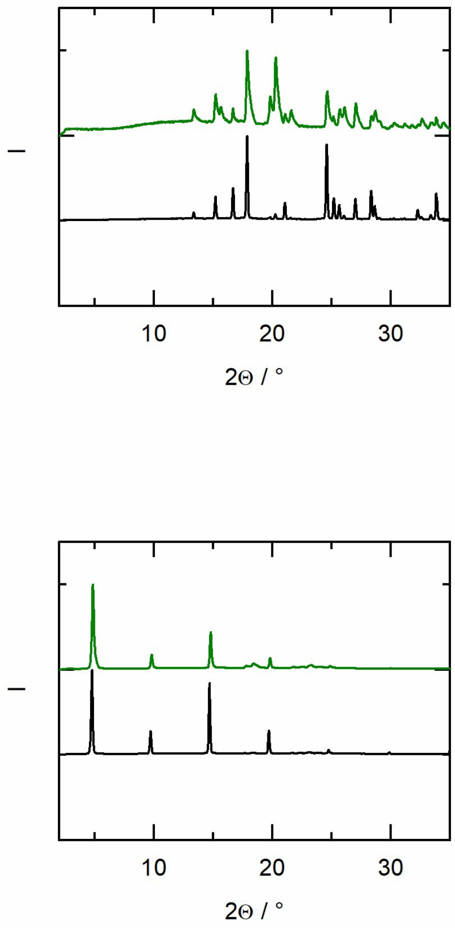

Figure S33 PXRD diffractograms of the amino acids Tyr (Top) and Trp (bottom). Green line: pure component; black line: solid phase of the supersaturated solution at T = 298.15 K and p = 1 atm.

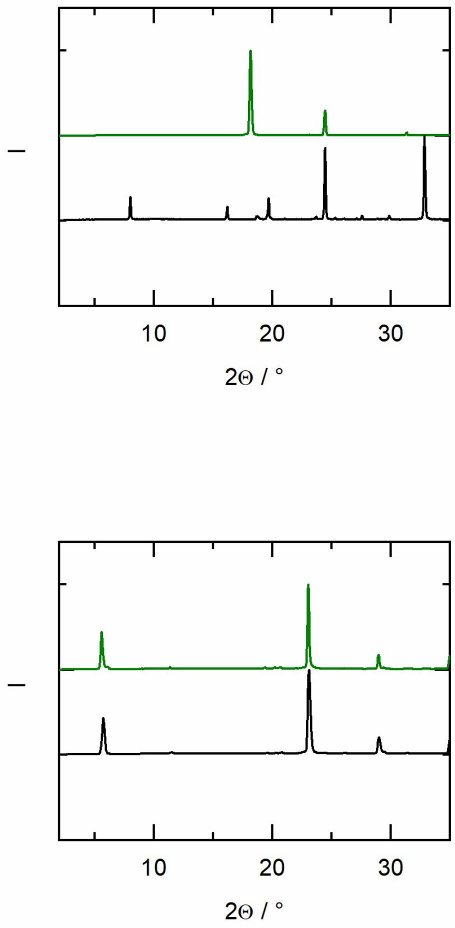

Figure S34 PXRD diffractograms of the amino acids Cys (Top) and Met (bottom). Green line: pure component; black line: solid phase of the supersaturated solution at T = 298.15 K and p = 1 atm.

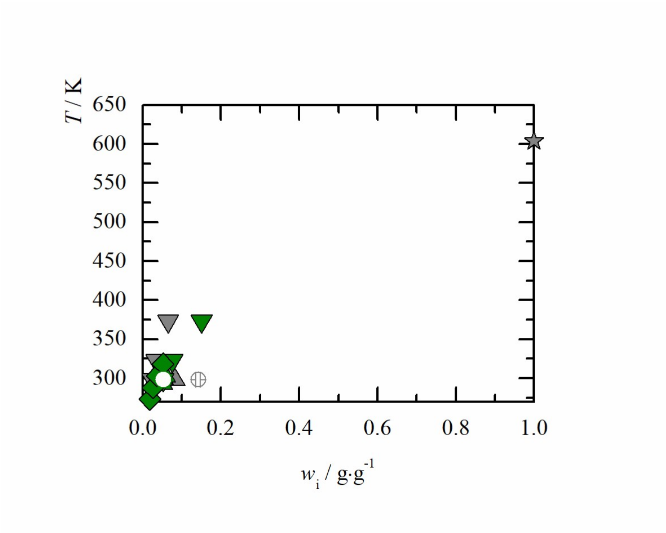

Figure S35 Temperature vs. weight fraction diagram. Solid symbols represent literature data. Empty circles represent the solubility measurements determined in this work. Solid circles represent melting temperatures measured in this work. **Cys.** ▲: Bowden[34], ▼: Amend, 1997[11]. **Met.** ▲ : Yalkowsky[26] ▼: Amend[11], ◆: Fuchs[35]

Table S1 Substances, abbreviations, suppliers, CAS numbers and mass-specific purities of the reagents used in this work.

Amino acids	*Abbrev.*	*Supplier*	*CAS no.*	*Purity [%]*
AA with non-polar substituents				
Glycine	*Gly*	*ROTH*	*56-40-6*	*≥ 99.0*
L-alanine	*Ala*	*Sigma*	*56-41-7*	*≥ 98.0*
L-valine	*Val*	*Alfa*	*72-18-4*	*> 99.0*
L-leucine	*Leu*	*TCI*	*61-90-5*	*> 99.0*
L-isoleucine	*Ile*	*Sigma*	*73-32-5*	*≥ 99.5*
L-proline	*Pro*	*Bachem*	*147-85-3*	*≥ 99.0*
AA with polar substituents				
L-serine	*Ser*	*Bachem*	*56-45-1*	*> 99.0*
L-threonine	*Thr*	*Sigma*	*72-19-5*	*≥ 98.0*
AA with acidic substituents				
L-aspartic acid	*Asp*	*Sigma*	*56-84-8*	*≥ 99.0*
L-asparagine	*Asn*	*Sigma*	*70-47-3*	*≥ 98.0*
L-glutamic acid	*Glu*	*Sigma*	*56-86-0*	*≥ 99.5*
L-glutamine	*Gln*	*Merck*	*56-85-9*	*> 99.0*
AA with basic substituents				
L-arginine	*Arg*	*Merck*	*74-79-3*	*≥ 98.0*
L-histidine	*His*	*Sigma*	*71-00-1*	*≥ 99.0*
L-lysine	*Lys*	*Sigma*	*56-87-1*	*≥ 98.0*
AA with aromatic substituents				
L-phenylalanine	*Phe*	*Merck*	*63-91-2*	*≥ 98.0*
L-tyrosine	*Tyr*	*Bachem*	*60-18-4*	*≥ 98.0*
L-tryptophan	*Trp*	*Merck*	*73-22-3*	*≥ 98.0*
Sulfur containing AA				
L-cysteine	*Cys*	*Merck*	*52-90-4*	*≥ 98.0*
L-methionine	*Met*	*Merck*	*63-68-3*	*≥ 99.0*

References

1. J.O. Hutchens, A.G. Cole and J.W. Stout, *J. Am. Chem. Soc.*, 1960, **82**(18), 4813.

2. J.O. Hutchens, A.G. Cole and J.W. Stout, *J. Phys. Chem.*, 1963, **67**, 1128.

3. A.G. Cole, J.O. Hutchens and J.W. Stout, *J. Phys. Chem.*, 1963, **67**(9), 1852.

4. J.O. Hutchens, A.G. Cole and J.W. Stout, *J. Biol. Chem.*, 1964, **239**(12), 4194.

5. J.O. Hutchens, A.G. Cole, R.A. Robie and J.W. Stout, 1963, **238**(7), 2407.

6. H.M. Huffman and E.L. Ellis, *J. Am. Chem. Soc.*, 1937, **59**(11), 2150.

7. J.O. Hutchens, A.G. Cole and J.W. Stout, *J. Biol. Chem.*, 1964, **239**(2), 591.

8. C. Held, L.F. Cameretti and G. Sadowski, *Ind. Eng. Chem. Res.*, 2011, **50**(1), 131.

9. Z. Yan, J. Wang, Liu W. and Lu Jinsuo, 1999.

10. R.L. Lundblad and F.M. Macdonald, *Handbook of biochemistry and molecular biology,* CRC Press, Boca Raton, FL 33487-2742, 2010.

11. J.P. Amend and H.C. Helgeson, *Pure & Appl. Chem.*, 1997(Vol. 69), 935.

12. C.M. Romero and M.E. González, *Fluid Phase Equilibria*, 2006, **250**(1-2), 99.

13. J.-B. Grosse Daldrup, C. Held, F. Ruether, G. Schembecker and G. Sadowski, *Ind. Eng. Chem. Res.*, 2010, **49**(3), 1395.

14. M. Kikuchi, M. Sakurai and K. Nitta, *J. Chem. Eng. Data*, 1995(40), 935.

15. P.K. Smith and E.R.B. Smith, 1937.

16. J.B. Dalton and C.L.A. Schmidt, *J. Biol. Chem.*, 1933, 549.

17. Z. Ronald C. and R. Ronald W., *Ind. Eng. Chem. Res.*, 1989(28), 1226.

18. L. Ninni and A.J. Meirelles, *Biotechnol Prog*, 2001, **17**(4), 703.

19. G.C. Barrett and D.T. Elmore, *Amino acids and peptides,* Cambridge University Press, Cambridge, 1998.

 This journal is © The Royal Society of Chemistry 20xx

Please do not adjust margins

20. C.-w.J. Luk and R.W. Rousseau, *Crystal Growth & Design*, 2006, **6**(8), 1808.

21. P.K. Smith and E.R.B. Smith, 1940.

22. L.A. Ferreira, E.A. Macedo and S.P. Pinho, *Fluid Phase Equilibria*, 2007, **255**(2), 131.

23. A. Apelblat and E. Manzurola, *J. Chem. Thermodynamics*, 1997(29), 1527.

24. Andrew W. Hakin, Michelle M. Duke, Lori L. Groft, Jocelyn L. Marty and Matthew L. Rushfeldt.

25. H. Matsuo, Y. Suzuki and S. Sawamura, *Fluid Phase Equilibria*, 2002(200), 227.

26. S.H. Yalkowsky, He, Yan and P. Jain, *Handbook of Aqueous Solubility Data,* CRC Press, Boca Raton, FL 33487-2742, 2010.

27. Q. Yu, X. Ma and L. Xu, *Thermochim. Acta*, 2013, **558**, 6.

28. O.D. Bonner, 1982.

29. E.N. Tsurko, R. Neueder and W. Kunz, *J Solution Chem*, 2007, **36**(5), 651.

30. A.V. Kustov and V.P. Korolev, *Thermochim. Acta*, 2006, **447**(2), 212.

31. C. Jolicoeur, B. Riedl, D. Desrochers, L.L. Lemelin, R. Zamojska and O. Enea, *Journal of Solution Chemistry*, 1986(15).

32. R. Carta and G. Tola, *J. Chem. Eng.*, 1996(41), 414.

33. D.P. Kharakoz, *Biophys Chem*, 1989(34), 115.

34. N.A. Bowden, J.P.M. Sanders and M.E. Bruins, *J Chem Eng Data*, 2018, **63**(3), 488.

35. D. Fuchs, J. Fischer, F. Tumakaka and G. Sadowski, *Ind. Eng. Chem. Res.*, 2006, **45**(19), 6578.

This journal is © The Royal Society of Chemistry 20xx

Please do not adjust margins
